# Children’s Oxygen Administration Strategies Trial (COAST):  A randomised controlled trial of high flow versus oxygen versus control in African children with severe pneumonia

**DOI:** 10.12688/wellcomeopenres.12747.2

**Published:** 2018-01-09

**Authors:** Kathryn Maitland, Sarah Kiguli, Robert O. Opoka, Peter Olupot-Olupot, Charles Engoru, Patricia Njuguna, Victor Bandika, Ayub Mpoya, Andrew Bush, Thomas N. Williams, Richard Grieve, Zia Sadique, John Fraser, David Harrison, Kathy Rowan

**Affiliations:** 1 Department of Paediatrics, Faculty of Medicine, Imperial College London, London, W2 1PG, UK; 2 Kilifi Clinical Trials Facility, KEMRI-Wellcome Trust Research Programme, Kilifi, UK; 3 Department of Paediatrics, Mulago Hospital, Makerere College of Health Sciences, Kampala, Uganda; 4 Mbale Clinical Research Institute, Mbale, Uganda; 5 Department of Paediatrics, Mbale Regional Referral Hospital, Mbale, Uganda; 6 Department of Paediatrics, Soroti Regional Referral Hospital, Soroti, Uganda; 7 Department of Paediatrics, Coast Provincial General Hospital, Mombasa, Kenya; 8 Department of Paediatric Respirology, National Heart and Lung Institute, Royal Brompton & Harefield NHS Foundation Trust, Imperial College, London, SW3 6NP, UK; 9 Department of Health Services Research and Policy, London School of Hygiene & Tropical Medicine, London, WC1H 9SH, UK; 10 The Critical Care Research Group, University of Queensland The Prince Charles Hospital and St Andrews Hospital, Clinical Science Building Rode Road, Chermside, QLD, 4032, Australia; 11 Intensive Care National Audit & Research Centre (ICNARC), London, WC1V 6AZ, UK

**Keywords:** Children, Africa, Emergency Care Hypoxia Pneumonia Randomised controlled trial, AirVO2 High flow oxygen, Permissive hypoxia

## Abstract

**Background:** In Africa, the clinical syndrome of pneumonia remains the leading cause of morbidity and mortality in children in the post-neonatal period. This represents a significant burden on in-patient services. The targeted use of oxygen and simple, non-invasive methods of respiratory support may be a highly cost-effective means of improving outcome, but the optimal oxygen saturation threshold that results in benefit and the best strategy for delivery are yet to be tested in adequately powered randomised controlled trials. There is, however, an accumulating literature about the harms of oxygen therapy across a range of acute and emergency situations that have stimulated a number of trials investigating permissive hypoxia.

**Methods:** In 4200 African children, aged 2 months to 12 years, presenting to 5 hospitals in East Africa with respiratory distress and hypoxia (oxygen saturation < 92%), the COAST trial will simultaneously evaluate two related interventions (targeted use of oxygen with respect to the optimal oxygen saturation threshold for treatment and mode of delivery) to reduce shorter-term mortality at 48-hours (primary endpoint), and longer-term morbidity and mortality to 28 days in a fractional factorial design, that compares:

1. Liberal oxygenation (recommended care) compared with a strategy that permits hypoxia to SpO _2 _> or = 80% (permissive hypoxia); and
2. High flow using AIrVO _2_^TM^ compared with low flow delivery (routine care).

**Discussion: **The overarching objective is to address the key research gaps in the therapeutic use of oxygen in resource-limited setting in order to provide a better evidence base for future management guidelines. The trial has been designed to address the poor outcomes of children in sub-Saharan Africa, which are associated with high rates of in-hospital mortality, 9-10% (for those with oxygen saturations of 80-92%) and 26-30% case fatality for those with oxygen saturations <80%.

**Clinical trial registration:** ISRCTN15622505

**Trial status: **Recruiting

## 1. Introduction

Worldwide, the clinical syndrome of pneumonia remains the leading cause of death in children in the post-neonatal period and therefore presents a significant burden on health services ^1, 2^. The greatest burden of the mortality is in South East Asia and sub-Saharan Africa where over 70% of these deaths occur annually ^3^. The World Health Organization (WHO) recommends presumptive antibiotic treatment based on clinical syndromic definitions of pneumonia plus oxygen for those with clinically very severe pneumonia (VSP) or when available to those with hypoxaemia – defined as oxygen saturation (SpO _2_) <90% measured by a pulse oximeter ^4^. Nevertheless, there is substantial evidence that current syndromic management guidelines are not working in practice, resulting in high in-hospital mortality (9–16%) ^5, 6^. Furthermore, severe pneumonia contributes to a ‘hidden burden’ of childhood mortality in the months following hospital admission, with those who survive severe pneumonia having a 2.5-fold increased risk of mortality post-hospital discharge compared to children admitted without severe pneumonia ^7^. The key risk factor for poor outcome has been identified as undernutrition ^8^. For those admitted to hospital, hypoxaemia (SpO _2_ <90%) is present on admission in 9.4% and 13.3% of children hospitalised with severe pneumonia (SP) and VSP, respectively, and is an important indicator of disease severity ^9^. The targeted use of oxygen and simple, non-invasive methods of respiratory support may be a highly cost-effective means of improving outcome, but both the optimal oxygen saturation threshold that results in benefit and the best strategy for delivery are yet to be tested in adequately powered randomised controlled trials (RCT). Oxygen as a potentially life-saving treatment, though advocated by the WHO technologies group, yet it has not been afforded a high enough priority in Africa or globally ^10, 11^. For example, in order to assess the health services ‘preparedness’ for pandemic influenza, 231 health facilities were surveyed across 12 African countries. They found that only 44% reported having uninterrupted oxygen supplies, with 24% possessing a functioning oxygen concentrator (the WHO preferred method for oxygen delivery) and reliable electricity power supplies were reported in 35% ^11^.

Systematic and policy reviews indicate the need for a formal evaluation of the hypoxaemia threshold at which oxygen should be targeted and of how oxygen is best administered ^9, 12, 13^. The COAST trial aims to address these key research gaps to provide a better evidence base for future guidelines, with a view to improving poor outcomes.

### 1.1 Clinical burden of pneumonia

The clinical syndrome of pneumonia remains the leading cause of childhood death in sub-Saharan Africa ^1^, despite public health interventions including immunization. Substantial investment in international multi-country research and vaccine implementation will undoubtedly reduce the burden of disease and lead to refinements of current guidelines. The current WHO clinical criteria for identification of pneumonia aim at prioritising sensitivity over specificity. Thus, emerging results from prospective studies of hospitalised children with WHO criteria for pneumonia indicate that it encompasses a much broader range of aetiologies, particularly in sub-Saharan Africa, than those likely to be averted by current and planned public health measures, such as immunisation programmes or early identification and pre-hospital antimicrobial treatment ^5, 14^. Adverse outcomes are not uncommon, despite early diagnosis and treatment ^5, 14, 15^. As the clinical syndrome of pneumonia is very common amongst hospitalised children, the high early mortality remains a significant barrier to improving survival statistics ^14– 16^, important for attainment of Sustainable Developmental Goals’ targets on health.

### 1.2 Hypoxaemia in ‘pneumonia’ syndromes

A systematic review examining the prevalence of hypoxaemia, including 24 published and unpublished data sets from Africa, Asia/Oceania and Latin America, reported a median prevalence of hypoxaemia for SP was 9.4% (interquartile range (IQR) 7.5–18.5%) and 13.3% (IQR 9.3–37.5%) for those with VSP ^9^. Mortality rates were not reported. Conservative estimates, based on these data, indicated that the annual worldwide burden of hypoxaemia in hospitalised pneumonia alone was between 1.5 and 2.7 million cases.

**Figure 1.**
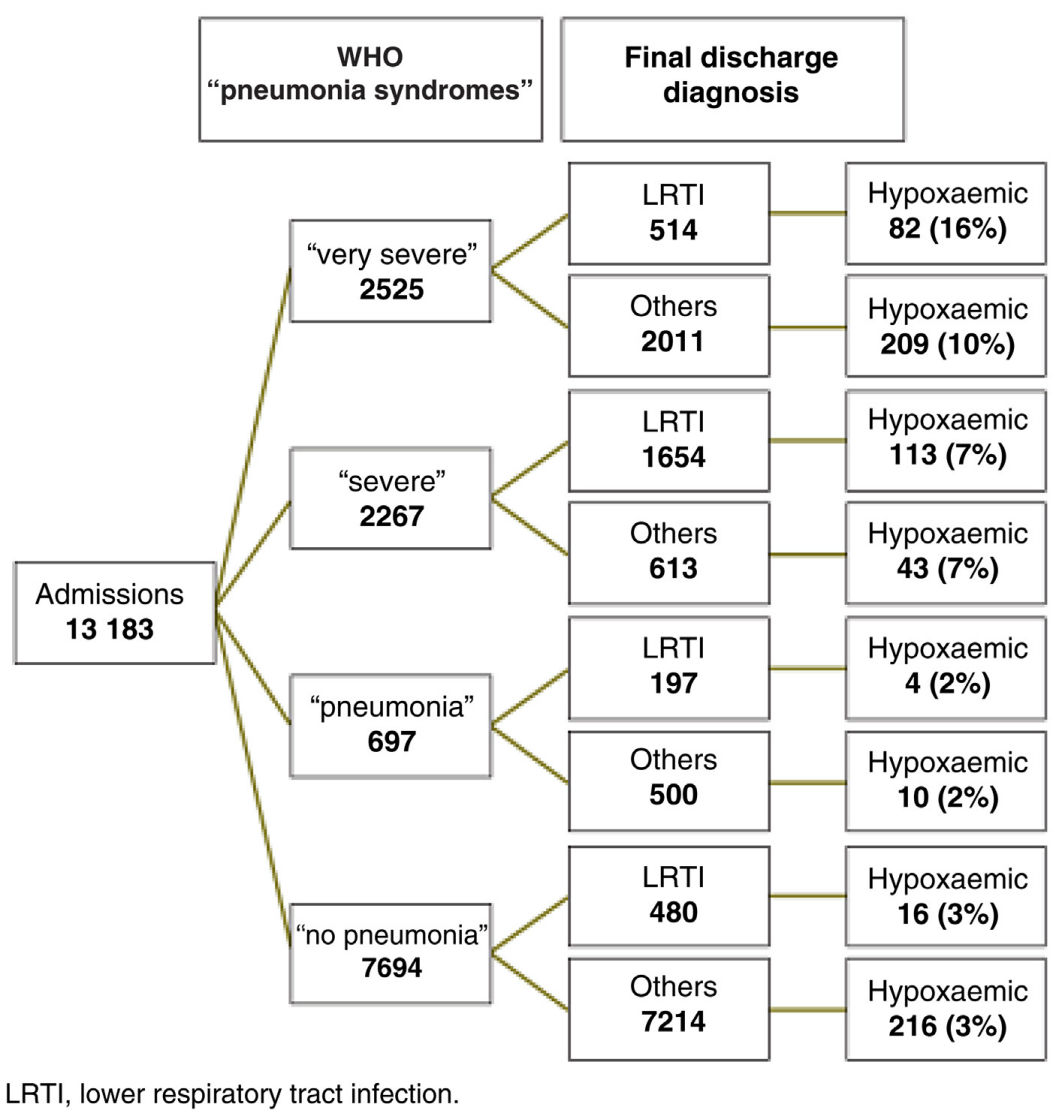
Lower respiratory tract infections and other non-respiratory diagnoses in 13,183 Kenyan children admitted to hospital (from Mwaniki *et al.* 2009; 5).

In an unselected paediatric (excluding neonates) admission population of 13,183 Kenyan children admitted to Kilifi District Hospital and where all admissions had a standard admission proforma, including pulse oximetry, if the WHO clinical criteria for severe and very severe criteria were applied then this would mean that 4792/13,183 or **36% of all admissions** would be classified as having VSP or SP; when in reality only 2168 (16%) had a final diagnosis of SP or VSP. Relevant to clinical practice (in hospitals where plus oximetry in not available) this would mean that if these were applied then 36% of all paediatric cases would fulfil criteria to receive oxygen. Yet in the Kilifi District Hospital studies only 291/2525 (11.5%) of children classified as VSP and 156/2267 (6.9%) of children classified as SP had hypoxaemia (oxygen saturation <90%) ( Figure 1) ^5^. Moreover, most of the children classified as VSP on admission, clinical criteria (2011; 80%) had another final diagnoses rather than pneumonia. **Most common causes of hypoxaemic were malaria (244, 35%), lower respiratory tract infection (LRTI) (221, 32%), malnutrition (68, 10%) and gastroenteritis (49, 7%).** Severe anaemia was found in 30 children (<1%).

### 1.3 Adoption of pulse oximetry in clinical practice

English *et al.* demonstrates that the use of pulse oximetry has been very poorly implemented across hospitals in Kenya despite it being recommended for triage of sick children ^17^. Between 2002 and 2009, none of 17 hospitals had a pulse oximeter, by 2012 only 3 of 22 (14%) of hospitals surveyed had a pulse oximeter. As a result, in most hospitals in Kenya, clinicians therefore prescribe oxygen ‘blind’ and since clinical signs poorly predict those with true hypoxaemia ^5^ ( Figure 1) a large number of children without hypoxaemia receive oxygen, which raises questions over safety ^18, 19^, cost and demand on scare health resources ^20^. In addition, in the absence of pulse oximetry, there is an even larger burden of children with hypoxaemia that are not identified for oxygen therapy.

### 1.4 Mortality associated with hypoxaemia

The importance of hypoxaemia, as a key predictor of mortality, is underlined in prospective studies. In the above study by Mwaniki *et al.,* overall 753 (6%) children died, including 150 (22%) of those with hypoxaemia. In Indonesia, children aged <2 years admitted with SP (overall 12% hospital mortality) demonstrated that hypoxic thresholds associated with increased mortality differed by age ^21^. For children aged <4 months, this was SpO _2_<88%, whereas for children aged >4 months it was <80%. Another study conducted in Kenya, found that children with hypoxaemia had a 5-fold increase of mortality compared to those without hypoxaemia ^22^.

Predicting mortality in sick African children (PET score) was developed from the FEAST trial ^23^. The FEAST trial was a controlled trial of fluid bolus therapy, which took place between 2009 and 2011 in six centres (including large regional referral hospitals and small district hospitals-none of which had access to mechanical ventilation) in three countries (Kenya, Uganda and Tanzania), in East Africa. In these three countries national vaccination programmes at the time of the trial included *Haemophilus influenzae* type B (HiB) vaccine, but did not include pneumococcal vaccination. Children, aged between 2 months and 12 years, with severe febrile illness were included in the trial (n= 3170) if they had additional features of severity (impaired consciousness or respiratory distress children) and clinical evidence of impaired perfusion ^24^, ISCRTN69856593). The primary outcome was 48-hour mortality. Prior to during the trial the clinical trial teams (clinicians and nurses) received Emergency Triage Assessment and Treatment (ETAT) training, with relevant guidance to ensure equipoise for the essential question the FEAST trial was addressing. Reliable supplies of oxygen therapy were only available in two of the six hospitals, and thus oxygen concentrators were provided for those lacking such equipment.

A secondary analysis of the FEAST trial data developed a clinical bedside risk score (the PET prognostic score) that examined clinical and laboratory prognostic factors for mortality using multivariable Cox regression to build a model in the no-bolus control arm of the FEAST trial. The aim was to develop a score, which was also externally validated on two admissions datasets from Kilifi District Hospital, Kenya, and compared to other published risk scores. The final model included 8 clinical variables (temperature, heart rate, capillary refill time, conscious level, severe pallor, respiratory distress, lung crepitations and a weak pulse volume), but notably did not include hypoxaemia. The strongest prognostic factors for mortality were coma, bradycardia (<80 beats per min) or severe tachycardia (>220 beats per min). Included in the PETAL score were additional laboratory markers lactate, urea and pH which added further information to the score. Oxygen saturation, although considered important in other studies, did not significantly improve the discriminative ability of the score in PETAL dataset and therefore not included in the final model.

### 1.5 Aetiology of pneumonia

The admission cohort of Kenyan children, reported above, demonstrated that 80% (n=2011) of children fulfilling VSP criteria had a non-pneumonic cause of respiratory distress ^5^. At the same centre, this was further explored in a case-control study examining pathogenic aetiology of children aged <5 years hospitalised with SP, in the era prior to the introduction of pneumococcal vaccine. Out of the 984 cases, 810 (84%) were eligible to be enrolled in the study. Of these 232 (29%) fulfilled WHO clinical signs of VSP. Out of the 749 with blood cultures result, 52 (7%) were positive in cases, of which 30/52 (58%) were *Streptococcus pneumoniae*. Bacterial aetiology was established in 23% (SPN=viral in 13%, mixed in 2% and 63% were of unknown aetiology) ^14^. There was no association found between most causes of viral infection of the nasopharynx and pneumonia, except for respiratory syncytial virus. No identifiable pathogen was found in any of the deaths. In a study conducted in Fijian children with a discharge diagnosis of LRTI, only 34% met WHO standardised criteria for chest x-ray confirmed pneumonia ^25^. Thus, after the introduction of the PCV vaccine, which has now been implemented across all the proposed sites for the COAST trial, the proportion of children with actual bacterial pneumonia will be substantially lower than the estimates approximately by 50%, generated from previous studies preceding the era of pneumococcal vaccination role out.

### 1.6 Current treatment recommendations for pneumonia

The WHO guidelines for management of children in hospital in resource-limited settings recommend presumptive antibiotic treatment based on clinical syndromes involving a history of cough or difficulty breathing with lower chest wall in drawing, or signs indicating VSP; it also recommends oxygen for those with VSP or hypoxaemia ( Table 1) ^4^. The normal range of acceptable SpO _2_ values differs according to age and altitude. At sea level, normal values are generally considered to be a SpO _2_ >94%. Thresholds for administering oxygen differ among international guidelines – some target <92%, whilst others target <94% (reviewed in Cunningham ^26^). The lack of evidence that maintaining ‘normal’ oxygen values in critically ill patients is beneficial, possibly explains the wide variation in practice surrounding the management of hypoxaemia. For children in resource-poor countries, the WHO recommended threshold for giving oxygen therapy is SpO _2_ <90%, or SpO _2_ <87% if living at altitude >2500m ^12^. In the 2013 WHO guidelines, the revision of the definitions of the categories of pneumonia mean that VSP and SP were collapsed into one category (‘Severe pneumonia’) and the definition included children with a history of cough and one or more of signs of putative pneumonia, including with respiratory distress, evidence of hypoxaemia (central cyanosis or oxygen saturations of <90%) or danger signs (lethargy or greater, convulsions). Oxygen is recommended for those with oxygen saturations of <90%, yet most hospitals in developing countries lack the facility to measure this ^17^.

**Table 1.** World Health Organization (WHO) guidelines recommended treatment for pneumonia.

Type	Where to treat	Oxygen
WHO 2005 recommendations ^3^		
**Very severe** **pneumonia**	Inpatient	• Give to all children with very severe pneumonia if pulse oximetry available, give to children with oxygen saturation <90% • Use nasal prongs (NP), nasal catheter, or nasopharyngeal catheter
**Severe pneumonia**	Inpatient	• If readily available, give to any child with severe lower chest wall in drawing or a respiratory rate of >70/minute
**Pneumonia**	Outpatient	• Not required
WHO 2013 recommendations ^27^		
**Severe pneumonia**	Inpatient	• Give if oxygen saturation <90% • Use NPs as the preferred method of oxygen delivery to young infants
**Pneumonia**	Outpatient	• Not required

### 1.7 Availability of therapeutic oxygen: Technical and clinical challenges

There is substantial evidence that even after substantial investment for influenza epidemic preparedness, there are significant gaps between supply and demand for oxygen in most hospitals in developing countries ^11^. Technical reports on the operational quality, availability and reliability of cylinders or oxygen concentrators indicate that, even when available, these are often faulty (substantial leakage from cylinders and lack of maintenance for both) and/or unsustainable due to high cost, erratic supply chain due to logistics and technical challenges (termed the ‘oxygen supply chain’) ^28^ or dependence upon unreliable electricity supply ^11, 29– 31^. However, in practice, this results in severe shortages of oxygen and intermittent supply of oxygen (where electrical power supplies permit).

Few hospitals have access to pulse oximeters ^32^ and the sustainable provision of bottled oxygen or oxygen concentrators are both expensive and logistically challenging ^33^. As a consequence of this and the high, often seasonal, burden of children fulfilling severe and very severe pneumonia definitions ^33^, many children who fulfil the current criteria for oxygen do not receive it (standard ‘usual care’). Instead, the current reliance on non-specific clinical signs to guide oxygen therapy results in the poorly targeted use of a costly intervention ^5^ – as a result few children who may benefit from oxygen therapy actually receive it.

The 2012 WHO Recommendations for management of common childhood conditions identified a number of key research questions as **‘Research Gaps’**, which included: large-scale effectiveness trials of improved oxygen systems on outcomes from pneumonia; and clinical studies comparing outcomes when oxygen is given at different thresholds ^12^.

### 1.8 Evidence from clinical trials and systematic reviews

Both inadequate tissue oxygenation and excessive oxygen administration (causing hyperoxaemia) are likely to be detrimental to outcome. These are two considerations that should inform evidence review for oxygen treatment guidelines: what is the evidence supporting beneficial use of oxygen and at what threshold (oxygen saturation); and what is the best method for its delivery? For the latter consideration, for resource-poor setting without access to intensive care (and mechanical ventilation), this will mainly consider non-invasive methods.

***1.8.1 Who needs oxygen?*** A Cochrane review conducted in 2005 ^13^ and updated in 2014 ^34^ examined evidence in the literature for: (i) indications for oxygen therapy, including both observational studies and RCTs comparing oxygen versus no oxygen therapy; and (ii) RCTs comparing methods of oxygen delivery for hypoxic LRTI in children aged three months to 15 years ^34^. Of the 551 articles assessed, there was no study that compared delivering oxygen with not delivering oxygen. Of the eligible studies, four RCTs compared delivery methods (see *section 1.12*) and 14 observational studies assessed the accuracy of clinical signs for indicating hypoxaemia. The review found no single clinical sign or symptom accurately identified hypoxaemia, although pneumonia complicated by hypoxaemia increased the risk of death ^35^. Wider implementation of pulse oximeters was recommended to identify those at greatest risk and for studies examining the most effective and safe method for oxygen delivery.

Thus, irrespective of the availability of mechanical ventilation, there have been no controlled trials of the use of oxygen supplementation that have incorporated a randomisation strategy of no oxygen. In resource-poor settings, there are limited relevant data from epidemiological studies for children receiving oxygen, or not, However, a review of available literature indicated little difference in mortality in children with pneumonia and SpO _2_ >80% receiving or not receiving supplementary oxygen ^36^. The only prospective observational study conducted to date examined mortality before and after improving systems for oxygen delivery in five hospitals in Papua New Guinea (that had no access to mechanical ventilation) ^10^. The study found a lowering mortality in children with pneumonia from 5.0% (95% confidence interval (CI) 4.5–5.5) to 3.2% (95% CI 2.7–3.8) for the 27 months after the improved system was introduced. Estimated costs of this system were US$51 per patient treated, US$1673 per life saved, and US$50 per disability-adjusted life-year averted ^16^. Nevertheless, there are a number of alternative explanations for improved outcomes, including possible benefits from training, improvements in the delivery of emergency care and/or other components of treatment, patient monitoring or temporal changes, which limit generalisability since the study was not designed to address direct benefits to the individual.

***1.8.2 Oxygen toxicity: The need for equipoise.*** Hypoxaemia is common amongst critically ill patients (with and without pneumonia) and usually is treated with oxygen and, where available and indicated, ventilatory strategies with the aim to restore arterial oxygenation to normal values and thereby reduce morbidity and mortality associated with hypoxaemia. This said, strategies used to treat hypoxaemic critically ill patients are also associated with harm, and this harm may outweigh the benefit of an increase in arterial oxygenation. Oxygen has been used in the treatment of pneumonia for a large part of the last century. However, the recognition of pulmonary oxygen toxicity as a problem has been relatively recent. Toxicity is related to the concentration of oxygen and length of exposure. Oxygen causes tracheobronchial irritation, reduced mucociliary function (even in healthy volunteers exposed to 90–95% oxygen for three hours ^37^) and, eventually, adsorption atelectasis, decreased vital capacity and changes similar to adult-type respiratory distress syndrome ^38^. The biochemical basis is considered to be due to damage to tissues through the production of reactive oxygen species. For these reasons it is recommended that inspired oxygen concentration (FiO _2_) should be carefully titrated against SpO _2_. However, oxygen toxicity has wider consequences, beyond the pulmonary system with animal models showing reduction coronary blood flow. Moreover, as haemoglobin is fully saturated with oxygen, supra-physiological oxygen tensions have little additional oxygen content compared to blood with physiological oxygen tensions.

***1.8.3 Conditions in which oxygen therapy are no longer recommended.*** In several areas of emergency care and resuscitation, emerging evidence from clinical trials and systematic reviews is now challenging the use of oxygen. In neonates, use of 100% oxygen during resuscitation increases mortality, myocardial injury and renal injury ^39^; even following an asphyxiating perinatal event, it is thought to increase the risk of cerebral damage ^40^. Current resuscitation guidelines in neonates now advise that the initial gas administered for ventilation should be room air ^41^.

In patients with an acute myocardial infarct, systematic reviews have found that, compared with room air, there is no evidence that oxygen therapy is of benefit ^42^; this is similarly the case in patients with an acute stroke ^43^. A recent trial in adults with asthma has shown that high concentration oxygen therapy results in a clinically significant increase in carbon dioxide (CO _2_), recommending careful titration of oxygen and only to those who are hypoxaemic ^44^. Much of the concern for optimising oxygenation has centred on neuroprotection or prevention of cognitive deficits in the long term, but the evidence supporting this is weak. In a follow-up study of mechanically ventilated adult patients with acute lung injury, a lower partial pressure of arterial oxygen (but not oxygen saturation) was associated with cognitive impairment ^45^. No equivalent studies are available for paediatric cohorts. A systematic review in children with chronic or recurrent hypoxaemia indicated adverse effects on development, behaviour, and academic achievement; however generalising these findings are complicated since most studies had small sample sizes and did not stratify by SaO _2_^46^.

With accumulating evidence that normoxaemia may not be a prerequisite to survival or successful recovery, opens up the possibility of permitting selected patients to remain hypoxic consequent upon their disease. Permissive hypoxaemia describes a concept in which a lower level of arterial oxygenation than usual is accepted to avoid the detrimental effects of inspired oxygen ^47, 48^. No specific threshold is known that defines permissive hypoxaemia, and its use remains formally untested.

### 1.9 Evidence for a threshold for delivery of oxygen

We were unable to find any data that prospectively determined a threshold for delivery of oxygen; in any setting, only observational studies reported mortality for those with and without hypoxaemia. One review from 1993 examined mortality in historic retrospective studies (1920’s) of children with acute respiratory tract infection with or without oxygen therapy ^36^. Whilst mortality rates were higher in those receiving and not receiving oxygen, these data are not informative since the *a priori* risk in the two groups are likely to be very different. In 2015, a parallel-group, randomised, controlled, equivalence trial of oxygen threshold strategies in infants aged 6 weeks to 12 months with clinician-diagnosed bronchiolitis was reported. The trial compared two strategies targeting an oxygen saturation threshold of 90% (n=308) and 94% (n=307). The primary outcome was time to resolution of cough (considered to reflect the degree of airway inflammation and thus influence the degree of hypoxaemia). In both arms, median time to cough resolution was 15 days (95% CI for difference –1 to 2), thus oxygen thresholds were equivalent. Adverse events were similar in both arms ^26^. The study concluded that management of infants with bronchiolitis to an oxygen saturation target of 90% or higher is as safe and clinically effective; this suggested further studies in older children examining oxygen thresholds are needed, particularly in developing nations where resources are scarce.

The WHO review of evidence, recommendation and key questions for future research is more comprehensive for the management of hypoxemia and oxygen delivery than any other of the WHO recommendations covering eight pages of the ‘Technical update of pocket book: evidence review of WHO recommendations’ ^12^. Pertinent to the questions being addressed by the COAST trial are those examining the use of oxygen therapy and delivery systems. These are given under the actual number section headings contained within the review (pages 69–76):

**Section 10.3 Oxygen therapy in treatment of hypoxaemia** (page 72)

a) Children with hypoxaemia should receive appropriate oxygen therapy. (Strong recommendation, low quality evidence)
b) Effective oxygen delivery systems should be a universal standard of care, and should be made more widely available. (Strong recommendation, expert opinion)

**Section 10.4 Thresholds for administering oxygen therapy** (page 73)

a) Administering oxygen therapy should be guided by pulse oximetry where available and thresholds for giving oxygen may vary depending on the altitude. *(Strong recommendation, very low quality evidence)*
b) Children living at ≤2500 m above sea level should receive oxygen therapy if their oxygen saturation is <90%. *(Strong recommendation, very low quality evidence)*
c) In children living at high altitude (>2500m above sea level), the normal oxygen saturation is lower than those living at sea level. At these altitudes, a lower level of saturation, such as SpO _2_ ≤87%, could be used as a threshold for giving oxygen. *(Strong Recommendation, very low quality evidence)*

In summary, most of the evidence for current recommendations is of very low quality; the use of oxygen is rated as expert opinion rather than based on evidence.

### 1.10 Evidence from other sources

**Figure 2.**
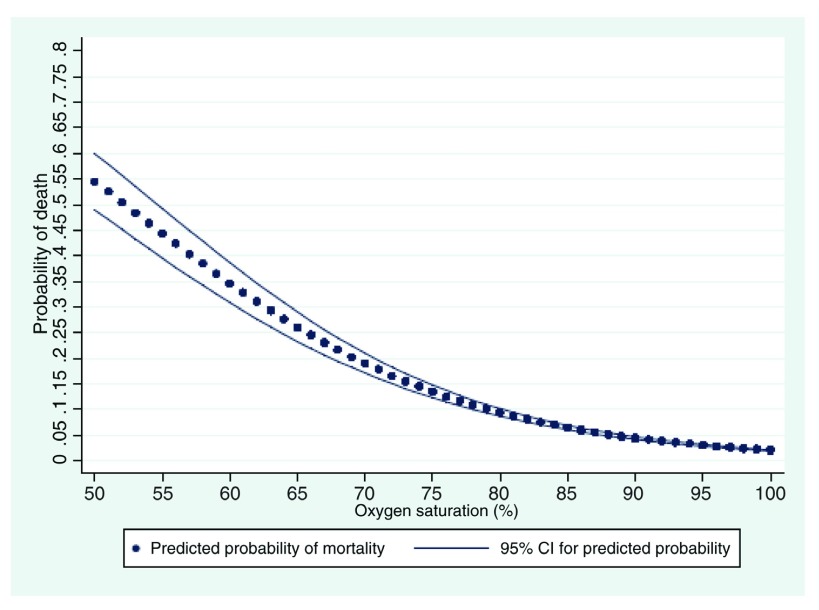
Relationship between baseline oxygen saturation and 48-hour mortality in 36,036 Kilifi paediatric general admissions.

**Figure 3.**
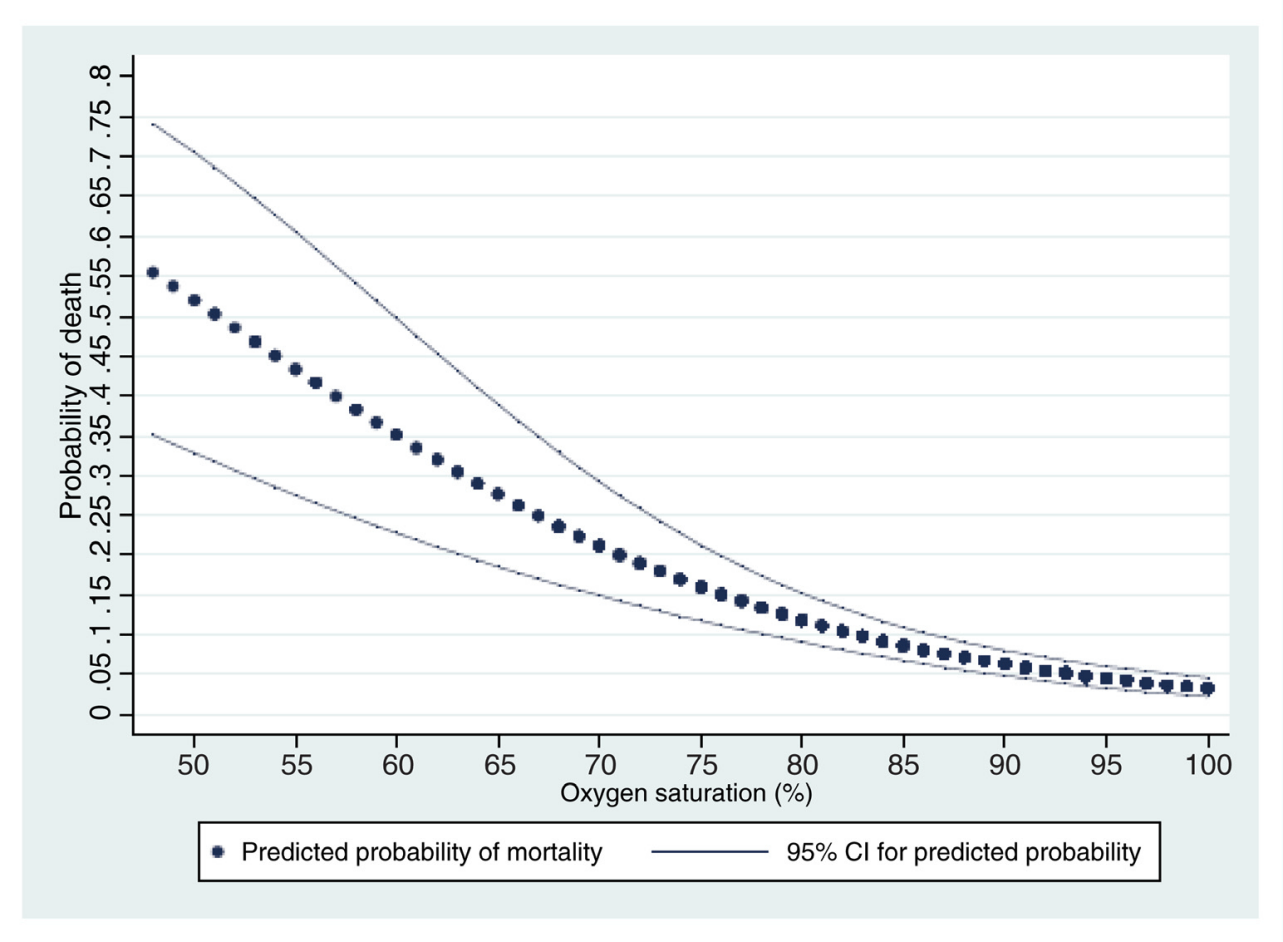
Relationship between baseline oxygen saturation and 48-hour mortality in the FEAST control arm (n=1007).

***1.10.1 Risk of mortality modelled against oxygen saturation.*** We examined the relationship between oxygen saturation at admission (or baseline) and mortality by 48 hours using a fractional polynomial logistic model restricted to the baseline measure, in the Kilifi (KEMRI Wellcome Trust Programme) dataset of paediatric general admissions (n=36,036; unselected admissions) ( Figure 2) (Maitland *et al*., unpublished data). The same relationship was also modelled and examined in more critically ill children using the control arm group of the FEAST Trial (n=1007) ( Figure 3) (Maitland *et al*., unpublished data). The trial eligibility criteria included children with severe illness (and shock), thus is equivalent to the inclusive definition of VSP (respiratory distress (83%) and/or impaired consciousness (72%)) ^24^. Both datasets indicated a steady increase of risk of mortality as SpO _2_ decreases from 100% to 80%, with a much higher risk of mortality at around 80%. The wider 95% CIs at low values in the FEAST dataset are due to low numbers, but is less pronounced for SpO _2_ ≥80%. Across the range 80–89% there were no statistical difference in the mortality (Day 2 mortality rates (Kilifi admission data) in children with COAST inclusion criteria: SpO _2_ of 80–82%(13%); 83–86%(10%); 87–89%(12%) (chi squared for trend P=0.5)).

Mortality in children with oxygen saturations 80–92% with severe febrile illness in the FEAST trial (control arm where not all children received oxygen) was 10% compared to 9% a KDH general admission population. For children with oxygen saturations < 80% in hospital mortality in the respective cohorts was 30% and 26%, respectively. Relevant to the design of the COAST trial is within the range 80–89% (<90% as per WHO guidelines where the oxygen-attributable preventable mortality (assuming that oxygen does prevent mortality) is very low because the mortality across this range of saturations is relatively flat with no statistical difference across the range.

### 1.11 Oxygen administration: Evidence for delivery methods

There are many ways of giving oxygen ^49, 50^; however, the WHO recommend NPs or NPCs for ease of delivery, patient acceptability and safety ^12^. Facemask delivery of oxygen requires much higher oxygen flow rates (to wash out the CO _2_), and feeding can interfere with its supply, thus is more costly. NPC requires the lowest oxygen flow rates, thus providing more reliable FiO _2_, but need careful insertion as, if misplaced, it could lead to gastric dilation or accidental dislodgement into the trachea. With NPs, a flow rate of 1–2 L/min in a child aged >4 months should provide a FiO _2_ of 30–35%; however, this varies with inspiratory flow, weight and degree to which the child breathes through their mouth ^51^. These are important logistical issues for optimising oxygen delivery. The most widely used method of non-invasive oxygen support is nasal continuous positive airway pressure (CPAP) which has been investigated as an alternative strategy for endotracheal respiratory support in order to reduce to complications of mechanical ventilation in infants and preterm infants ^52^. It has also been investigated as a method for respiratory support in low resource settings ^53^.

***1.11.1 High flow oxygen therapy.*** An alternative strategy than CPAP is high flow nasal cannula (HFNC) oxygen delivery. HFNC is a relatively newer form of non-invasive respiratory support that delivers a mixture of air and oxygen (blended gas) via dedicated bi-nasal cannula that fit snugly with the nares to minimise leakage. Heated, humidified, high-flow (such as AirVO _2_^TM^), deliver blended oxygen air at flow rates over 1 L/min through bi-nasal cannula which smaller than those of NCPAP and with prongs positioned to sit just inside the nares and do not provide a seal. It is more comfortable for the infant, results in less nasal trauma than other forms of respiratory support ^54^, it is easy to implement and as a result increasingly used globally. HFNC results in incidental delivery of positive end expiratory pressure (PEEP) ^55– 57^. Whilst not fully understood it has been suggested that the high flow ‘flushes out’ pharyngeal dead space, resulting in a positive expiratory pressure effect, improved alveolar recruitment and, greater humidification, and a better control of inspired oxygen fraction ^55, 58^. HFNC therapy has also been reported to be better tolerated by the patient than other forms of non-invasive ventilation, partly due to the use of HFNC oxygen therapy with heated and humidified gases ^59^.

Some studies, but not all, have shown AirVO _2_^TM^ oxygen devices reduce the requirement for positive pressure ventilation in the paediatric population with respiratory failure secondary to infection ^60, 61^. The AirVO _2_^TM^ device ( Fisher & Paykel HealthCare) is adaptable for children over a wide weight and age range and is capable of providing sustainable, non-invasive respiratory support generating high flows through a self-contained turbine-driven device ( ACTRN12613000028707), without the need for specialized masks or endotracheal intubation – all of which are significant financial and technical barriers for use in resource-poor settings.

### 1.12 Trials and systematic reviews of oxygen delivery systems

A Cochrane review (update) conducted in 2014 identified four studies (479 participants) assessing the efficacy of non-invasive delivery methods for the treatment of pneumonia in children ^34^. Three RCTs (399 participants) compared the effectiveness of NPs or nasal cannula with NPCs and one non-RCT (80 participants) compared head box, face mask, NPCs (control) and nasal cannula. The use of a face mask showed a statistically significant lower risk of failure to achieve arterial oxygen > 60 mmHg than the nasopharyngeal catheter (one non-RCT; 80 participants; odds ratio (OR) 0.20, 95% CI 0.05 to 0.88). The use of a head box showed a non-statistically significant trend towards a reduced risk of treatment failure compared to the NPC (one non-RCT; OR 0.40, 95% CI 0.13 to 1.12). NPs are associated with fewer nasal obstruction problems. The quality of the evidence was judged to be very low.

### 1.13 Recent randomised controlled trials

The recent eight centre, 618 patient clinical trial in the UK (the Bronchiolitis of Infancy Discharge Study; BIDS ^26^) showed that management of infants with bronchiolitis to an oxygen saturation target of 90% or higher is as safe and clinically effective as one of 94% or higher. The authors suggested that future research should assess the benefits and risks of different oxygen saturation targets in acute respiratory infection in older children, particularly in developing nations where resources are scarce.

An open, randomised, controlled trial in a referral hospital in Dhaka in 225 children < 5 years with severe pneumonia and hypoxaemia randomized children to receive oxygen therapy by either bubble CPAP (5 L/min starting at a CPAP level of 5 cm H2O), standard low-flow nasal cannula (2 L/min), or high-flow nasal cannula (2 L/kg per min up to the maximum of 12 L/min) ^53^. The primary outcome was treatment failure (i.e.clinical failure, intubation and mechanical ventilation, death, or termination of hospital stay against medical advice) after more than 30 minutes of treatment. In total, 79 (35%) children were randomized to receive oxygen therapy by bubble CPAP, 67 (30%) to low-flow oxygen therapy, and 79 (35%) to high-flow oxygen therapy. The trial was stopped twice once after the first interim analysis and finally after the second interim analysis after only 25% of the predicted sample size were enrolled, on the recommendation of the data monitoring committee. Treatment was declared to have failed for 31 (14%) children, of whom five (6%) had received bubble CPAP, 16 (24%) had received low-flow oxygen therapy, and ten (13%) had received high-flow oxygen therapy. Treatment failure was lower in the CPAP group than in the low-flow oxygen therapy group (relative risk [RR] 0.27, 99.7% CI 0.07-0.99; p=0.0026). No difference in treatment failure was noted between patients in the bubble CPAP and those in the high-flow oxygen therapy group (RR 0.50, 99.7% 0.11-2.29; p=0.175). 23 (10%) children died. Three (4%) children died in the bubble CPAP group, ten (15%) children died in the low-flow oxygen therapy group, and ten (13%) children died in the high-flow oxygen therapy group, indicating that bubble CPAP group had significantly lower rates of death than the children who received oxygen by low-flow oxygen therapy (RR 0.25, 95% CI 0.07-0.89; p=0.022). The authors concluded that bubble CPAP improved outcomes in Bangladeshi children with VSP and hypoxaemia compared with standard low-flow oxygen therapy, but the early cessation of the trial reduces the certainty of the findings and suggested that further research was required before guidelines could be changed.

An accompanying editorial was highly critical of the early stopping of the trial flagging the perils of early stopping for apparent efficacy ^62, 63^, which may provide misleading results and that small sample sizes can lead to random variation often causing large treatment effects (randomly either spuriously too high or too low) that diminish as the trial progresses (widely recognised as ‘regression to the truth’) ^64^. Quoting from an authority on clinical trial methodology, the editorial recommended to “Avoid any analysis (or even brief inspection) of the data until some dozens of deaths have accumulated, for it is trials first looked at when the number of events are very small that are most likely to be misleading’’ ^65^. This author had developed the Haybittle-Peto rule for early stopping of a trial (indicating a p value of <0.001) ^65^. An independent statistician had recommended, after the trial was initially stopped after the first interim analysis, in future to use a stopping rule with p value <0.003; this advice was not taken. Furthermore, there are concerns that the rescue strategy in the protocol for children for whom low-flow oxygen therapy failed would have a trial of bubble CPAP or high-flow oxygen therapy before intubation and mechanical ventilation, which was the standard rescue therapy for CPAP and high-flow arms.

### 1.14 Rationale for a controlled trial

The high incidence and mortality associated with hypoxaemia in children with pneumonia and severe illness at hospitals constitutes a major public health challenge. Options for supportive care in hospitals without access to mechanical ventilation are limited. At present, there is uncertainty as to what the best cut-off threshold for cost-effective oxygen delivery is and whether the addition of high flow is safe and results in improved outcome. Since hypoxaemia is so common and treatment costs and practicalities for optimal delivery of oxygen are complex, the COAST trial will contribute substantial new knowledge in this area. The COAST trial was proposed and discussed at the launch meeting of ISARIC in Annecy, July 2012. The meeting brought together many leading researchers and clinical trialists in severe respiratory illnesses with representation from international agencies, such as the WHO. The proposal had wide support as it was anticipated that the trial would result in refinements to the current WHO recommendations and provide robust evidence to support the targeting of oxygen to those with the greatest need and averting unnecessary consumption in financially constrained hospitals. The pragmatic nature of COAST and locations chosen will mean that the results of the trial will be immediately generalisable. The successful conduct of the FEAST Trial acts as a pilot for the conduct of COAST. The proposed high flow oxygen delivery device is in current use in South Africa, and feasibility testing and staff training in the COAST sites will occur before trial commencement. The COAST trial was preceded by the Paediatric Oxygenation Strategies Treatment Study (POST), a feasibility study, which assessed feasibility of both the intervention and the RCT processes (including testing whether high flow oxygen via the AirVO _2_^TM^ device can be used effectively in a hospital in Africa). The POST study helped inform the trial protocol for COAST (Maitland *et al*., manuscript in preparation).

***1.14.1 Research gap: Timeliness for a clinical trial.*** The international charity Lifebox launched a campaign to close the global ‘pulse oximetry gap’ (Make It Zero). As more hospitals acquire pulse oximeters, the hidden ‘hypoxaemia burden’ will be recognised and demands for an equitable supply of oxygen will increase. A recent commission from a collaboration between The Lancet and Imperial College, examining how medical technology could be used to improve health in low- and middle-income countries, identified technologies, such as oxygen delivery, as future priorities ^66^. Nevertheless, important lessons were learnt in the FEAST Trial where fluid resuscitation, part of emergency care for decades in well-resourced countries, resulted in worse outcome in the fluid bolus arms ^24^. Simple translation of established medical practice, largely based on a low quality of evidence, to resource-poor countries should be preceded by definitive trials to generate the evidence for best practice and cost-effectiveness. The COAST trial will provide the relevant evidence supporting such an initiative.

## 2. Trial protocol

**Table:** 

PICO: Population – Intervention – Comparison – Outcome			
Population	Intervention	Comparison	Outcome
- Pregnancy - Patient Currently Pregnant - Pregnancy Nos - Newborn - Twin Pregnancy	- Cesarean Section - Vaginal Delivery Of Fetus - Providing Care According To Standard		- Cesarean Section - Vaginal Delivery Of Fetus - Providing Care According To Standard

To learn more about PICO – please visit https://en.wikipedia.org/wiki/PICO_process

### 2.1 Trial aim and objectives

The COAST trial will investigate what the best oxygen delivery strategies are to reduce in-hospital mortality and morbidity in African children with respiratory distress complicated by hypoxaemia (defined as SpO _2_ <92%). This will be done by evaluating two linked components of oxygen delivery:

1. whether liberal oxygenation is superior to permissive hypoxaemia (current routine standard of care in hospitals not screening all admissions for hypoxaemia); and
2. whether high flow oxygen delivery is better than low flow oxygen delivery (current routine standard of care in hospitals with no access to mechanical ventilation).

Both the primary and secondary objectives will address what threshold to deliver oxygen (above a threshold of SpO _2_ ≥80%).

### 2.2 Primary objectives

To establish whether:

- liberal oxygenation will decrease mortality compared with permissive hypoxaemia (usual care); and
- high flow oxygen delivery will decrease mortality compared with low flow oxygen delivery (usual care).

### 2.3 Secondary objectives

The secondary objectives of COAST are:

- to assess the effectiveness in the speed and success of recovery from the initial respiratory failure;
- to assess safety and quality of life;
- to determine long-term effects of the delivery strategies; and
- to identify whether the additional costs of each of the interventions are proportionate to the health benefits and to inform future widespread implementation in terms of value for money.

## 3. Trial outcome measures

### 3.1 Primary

The primary outcome measure is mortality at 48 hours post-randomisation.

### 3.2 Secondary

The secondary outcome measures are:

- Treatment failure at 48 hours (i.e. still receiving oxygen/respiratory support)
- Survival to 28 days
- Neurocognitive sequelae ( *de novo* or worsening of existing neurological impairment) at 28 days
- Disability-free survival to 28 days
- Time to hypoxaemia (≥92%) resolution during initial hospital stay
- Length of initial hospital stay
- Re-admission to hospital by 28 days
- Anthropometric status by 28 days
- Resolution of neurocognitive sequelae at 90 days (for those with neurocognitive sequelae at 28 days *(see above)*)

### 3.3 Trial design

COAST is an open, multicentre, fractional factorial RCT of 4,200 participants, aged from 28 days to 12 years, admitted to hospital with respiratory distress complicated by hypoxaemia. Participants will be enrolled over a 30-month period and followed up to 28 days post-randomisation (and at 90 days for those with neurocognitive sequelae at 28 days).

### 3.4 Benefits and risks

***3.4.1 Benefits.*** In conducting COAST, we will identify the hidden burden of hypoxaemia; therefore, more children are likely to benefit than if the trial was not conducted in the first place. In FEAST, we witnessed substantial improvements in outcomes in the hospitals where the trial was taking place. Outcomes of participants in trials are often substantially improved. Extra clinical personnel, regular patient clinical assessment and the basic equipment for continuous patient monitoring are available during the trial so that if the complications above were to arise they could be detected and treated. Pre-trial training to include sign recognition for these complications and training on treatment. Both these are covered in detail in the Manual of Operations, which will be available on the ward.

**3.4.1.1 For the child**

The direct benefits to the child and/or family (outlined in the Patient Information Sheet, Supplementary File 1) include:

- The consent process, as in the FEAST Trial ^67^, will allow parents/guardians time to weigh up benefits and risks before giving deferred consent.
- Closer observation during the first 48 hours of admission, which, as a result, allows doctors and nurses to make important changes to the child’s treatment during in-hospital admission.
- All routine non-trial medications required by the hospital to treat the child will be made available (when unavailable, parents/guardians will have to resort to sourcing these privately).
- The parents/guardians of participating children will be asked to return for follow-up at 28 and 90 days after admission (only for those with suspected neurological sequelae at Day 28). Reimbursement for transport cost after discharge and for follow-up visits, plus any treatment costs required during the visits, will be made.

**3.4.1.2 For the hospital**

The direct benefits to the hospital include:

- Substantial investment in training and provision of equipment and consumables to provide oxygen will improve the treatment of all critically sick children.
- Pulse oximeters will routinely be used in triage, thus improving the specificity of the clinical criteria for oxygen-use – this can then be directed to those with hypoxaemia and reduce potential wastage of this resource to children who do not require oxygen therapy.
- Additional staff, employed directly for the trial, who are likely to manage the sickest patient (those at high risk of hypoxaemia and thus eligibility for the trial), will help relieve the treatment burden on acute paediatric services.

**3.4.1.3 For health personnel**

The direct benefits to health personnel are mainly professional development of the members of the trial teams and clinical teams for the purposes of running the trial, including basic life support courses, clinical trials training and research training.

***3.4.2 Risks***

**3.4.2.1 Oxygen saturation threshold**

Children are carefully monitored, including active reporting of solicited adverse events (AEs). Training of trial staff, as in FEAST, to ensure that trial participants receive recommended standard of care and careful monitoring. Expected inherent hazards of the intervention strategies, solicited and serious adverse events (SAEs), as defined by ICH-GCP guidelines, are secondary outcomes of the trial; these will be reported in real time and assessed by an independent Endpoint Review Committee (ERC). We have included within the protocol oxygen to be given (by low flow) for all children whose saturations are ever <80% to minimise harm. The ICH-GCP compliant SAE data collection and reporting procedures will be adopted. Clinical staff at sites will be trained by the Chief Investigator (ChI) and training team at the initiation visit to recognise expected side effects.

**3.4.2.2 High flow versus low flow**

High flow is an accepted strategy in the management of children with respiratory failure in a multitude of countries, with few reports that it is unacceptable, so the risks of harm from this strategy are known and are extremely low.

**3.4.2.3 Deficiencies in oxygen supply**

We will use bottled oxygen, whenever this is available. When supplies are low, we will use oxygen concentrators (at least three per site) and a reliable ‘power back’ system of electricity. The backup system will use a bank of batteries and charge during periods of electricity supply. If there are power outages, it will automatically switch on to provide backup power for up the 12 hours. If there are long periods of power outages (though unusual), then the trial teams will stop screening patients and not enroll into the trial until power is restored. A maximum of four patients per day will be recruited to alleviate potential burden on the available oxygen supplies so that this can be used in children outside of the trial (including cases where consent is declined).

## 4. Selection of sites

Five hospitals in two countries will participate:

- Kenya: KEMRI Wellcome Trust Programme, Kilifi County Hospital (KCH). The clinical programme at KCH is a well-established research centre on the coast of Kenya. It has been the site of several clinical studies on severe malaria, including two large phase III trials (AQUAMAT and FEAST). Coast Provincial General Hospital (CPGH) is based in the second largest city in Kenya (Mombasa) and has conducted in collaboration with KWTRP a number of research studies and Phase III clinical trials in children admitted to CPGH.
- Uganda: Mulago National Referral Hospital, Mbale Regional Referral Hospital and Soroti Regional Referral Hospital. Mulago, Mbale and Soroti hospitals have annual admissions to the paediatric ward of 18,000, 20,000 and 8000, respectively. There has been considerable research capacity development in the last five years, with these three sites involved in the FEAST trial and currently conducting a large multicentre trial of transfusion trial (TRACT).

The five centres in Africa represent a spectrum of intensity of malaria transmission, from perennial and high (in Mbale and Soroti) to seasonal and meso-endemic (in Kampala, Mombasa and Kilifi). Mulago and CPGH are located within large urban areas, whereas the populations that utilise Mbale, Soroti and Kilifi are more typically rural; however Mbale has a few slum developments within the town.

Central grant funding will be available for participating sites. To participate in the trial, selected hospitals and principal investigators (PI) must fulfil the criteria defined below.

### 4.1 Site/investigator inclusion criteria

The site/PI should:

1. Be qualified by education, training, and experience to assume responsibility for the proper conduct of the trial at their site and should provide evidence of qualifications through an up-to-date curriculum vitae (CV) and/or other relevant documentation requested by the Sponsor, the Research Ethics Committee (REC) or Institutional Review Boards (IRB) and/or the regulatory authority(ies).
2. Be aware of, and should comply with, the principles of ICH-GCP and the applicable regulatory requirements. A record of GCP training should be accessible for all trial staff at the site.
3. Have available or appoint an adequate number of qualified staff for the duration of the trial to conduct the trial properly and safely. Trial staff should be adequately informed about the protocol, trial equipment and their trial-related duties. The site should maintain a Delegation Log of appropriately qualified persons to whom the PI has delegated trial-related duties.
4. Be able to demonstrate a potential for recruiting the required number of participants and have sufficient time to conduct the trial as per protocol and complete the trial within the agreed trial period.
5. Permit monitoring and auditing by the Sponsor, and inspection by the appropriate regulatory authority(ies).

### 4.2 Documentation

The following documentation must be in place prior to a trial site being opened to recruitment:

- Investigator Statement – this verifies that the site is willing and able to comply with the requirements of the trial and will be signed by the site PI;
- Signature Form – this will be signed by site staff delegated trial responsibilities by the PI; and
- Delegation Log and staff contact details.

All completed documents should be sent to the Kilifi Clinical Trial Facility (KCTF). Up-to-date copies of the Delegation Log and contact details (with any changes to trial personnel and/or their responsibilities) should be sent to the KCTF and a copy should be stored in the Investigator Site File (ISF) at the site.

### 4.3 Activation

On receipt of all the documents, written confirmation will be sent to the PI, at which point the site may start to screen for eligible patients.

Once the site is activated, they should ensure:

- adherence to the most recent version of the protocol;
- appropriate recruitment and care for participants in the trial;
- timely data entry; and
- prompt notification of all AEs and protocol deviations to the trial team at the KCTF.

## 5. Selection of participants

### 5.1 Screening

The trial will run 24 hours/seven days per week. Patients will be assessed for eligibility at the point of hospital admission by the dedicated and trained trial nurse or clinician (on trial delegation log). Established triage systems will identify potential patients who will be screened against the inclusion/exclusion criteria; a member of the trial team will carry out a rapid structured assessment of heart rate, oxygen saturation (pulse oximetry), respiratory rate, axillary temperature, blood pressure, markers of shock (capillary refill time, pulse volume and assessment of lower limb temperature) and severity (conscious level and respiratory distress). Entry criteria will be based on clinical assessment alone; it is anticipated that this process will take five minutes.

Infants and children aged between 28 days to 12 years who are potentially eligible for inclusion will be fast tracked to the trial team for full eligibility screening and assent/consent for enrolment. Children who do not meet the eligibility criteria at initial assessment will be re-screened for eligibility up to 24 hours post-hospital admission.

Sites will maintain a record of all patients who are screened for eligibility for COAST:

- Eligibility Screening Log – will record all potentially eligible patients (who fulfil the inclusion criteria, but meet one or more of the exclusion criteria); and
- Screening Form – will record all patients who meet the full eligibility criteria (with reasons for non-randomisation).

### 5.2 Inclusion criteria

- Aged between 28 days to 12 years
- History of respiratory illness (cough, upper respiratory tract symptom or any respiratory symptoms, e.g. rapid breathing or increase work of breathing)
- Hypoxaemia (pulse oximetry reading of SpO _2_ <92% recorded in room air over 5 minutes)

a. Plus suspected severe pneumonia informed by WHO guidelines, as below - Sign of respiratory distress (any one of):
  ○ severe lower chest wall in-drawing
  ○ use of auxiliary muscles
  ○ head nodding
  ○ inability to feed because of respiratory problems

b. Suspected pneumonia
  ○ fast breathing:
    ▪ age 2–11 months: ≥ 50/minute
    ▪ age 1–5 years: ≥ 40/minute
    ▪ age 5–12 years ≥ 30/minute
  ○ chest auscultation signs:
    ▪ decreased breath sounds
    ▪ bronchial breath sounds
    ▪ crackles
    ▪ abnormal vocal resonance (decreased over a pleural effusion or empyema, increased over lobar consolidation)
    ▪ pleural rub

c. Signs of pneumonia with a general danger sign:
  ○ inability to breastfeed or drink
  ○ lethargy or unconscious
  ○ convulsions

### 5.3 Exclusion criteria

- Known uncorrected cyanotic heart disease
- Assent/consent refusal by parent/carer
- Previously recruited to COAST
- Already received oxygen for this episode of illness (at another health facility)
- Known chronic lung disease (not including asthma)

## 6. Enrolment

### 6.1 Consent

Once eligibility has been confirmed, authorised trial staff will approach parents/guardians to invite their child to take part in the trial. An information sheet will be provided to the parent/guardian in their usual language containing details of the COAST trial. The sheet will be read aloud to those who are unable to read. The doctor/nurse will check that the information has been fully understood and parents/guardians will be encouraged to ask questions they may have about their child’s participation in COAST.

Where possible, prospective written informed consent will be sought from parents/guardians who will then be asked to sign the Consent Form. If parents/guardians are unable to sign, a thumbprint will be taken in lieu of a signature.

If it is considered that the full consent process would significantly delay treatment allocation, and consequently be detrimental to the child’s health, then emergency verbal assent, used in the FEAST and TRACT trials ^67^, will be sought from parents/guardians by the admitting medical team (see *section 7.2*).

### 6.2 Emergency verbal assent followed by deferred consent

It is likely that, due to the emergency nature of the patients’ condition, the full consent process may delay treatment. If this is the case, then parents/guardians will be provided with a brief verbal description of the trial and will be given the opportunity to “opt out” of clinical research. Full consent will be sought once the child’s clinical condition has been stabilised. The clinician will later sign the Verbal Assent Form, ( Supplementary File 2) which will be filed with the Consent Form. If consent is withdrawn later, trial data collected up to the time of withdrawal will only be used and no further trial specific procedures will be conducted (see *section 7.5*).

A copy of the Consent Form will be given to the parent/guardian, the original placed in the patient’s medical notes, and a copy kept in the Investigator Site File.

### 6.3 Randomisation

The trial has two strata: **COAST A**: severe hypoxaemia, SpO _2_ <80%); and **COAST B**: hypoxaemia (SpO _2_ ≥80% and <92%).

Children in COAST A (2-arm, 1:1 ratio) will all receive oxygen and randomisation will allocate participants to one of two methods of oxygen delivery:

i . high flow oxygen delivery
ii. low flow (usual practice) oxygen delivery

Children in COAST B (3-arm, 2:1:1 ratio) will be allocated to

i. permissive hypoxaemia (no immediate oxygen); control
ii. high flow oxygen delivery
iii. low flow (usual practice) oxygen delivery

Participants will be allocated to treatment arms by a computer-generated list using random permuted blocks. The blocks will be stratified by trial site and baseline SpO _2_ <80% or ≥80%. The Trial Statistician at the Intensive Care National Audit & Research Centre (ICNARC) Clinical Trials Unit (CTU) will prepare the randomisation lists before the trial commences and kept at the ICNARC CTU, London. Opaque and sealed randomisation envelopes will be prepared and provided to each site, with one set for COAST A (SpO _2_ <80%) and one set for COAST B (SpO _2_ ≥80% and <90%). The envelopes for each site will be numbered consecutively and opened in numerical order. These will contain details of the treatment arms once opened.

This system has worked well in the emergency care trials, i.e. FEAST and TRACT (transfusion trial). To facilitate protocol adherence, a maximum per site will be agreed upon (for example up to 4 children per day) will be enrolled per site. This approach was very successful with respect to protocol adherence and the quality of data generated in the FEAST trial.

### 6.4 Co-enrolment

Participants will not be permitted to co-enroll in any other interventional studies while on COAST. Participation in studies that do not involve an intervention (e.g. observational studies) are acceptable, but should be discussed with the COAST Trial Meeting Group (TMG). The TMG will consider co-enrolment of COAST participants onto other observational studies where the management does not conflict with the COAST objectives on a case-by-case basis.

### 6.5 Withdrawal

In consenting to the trial, parents/guardians and their children are consenting to trial treatment, assessments, data collection and follow-up. However, children or their parents/guardians can withdraw from COAST at any time during the trial.

If a child, or their parents/guardians, chooses to withdraw any part of their trial treatment, they should always be followed-up and encouraged not to leave the full trial. The treating clinician remains free to give alternative treatment to that specified in the protocol at any stage if he/she feels it is in the participant’s best interest, but the reasons for doing so should be recorded. In these cases, the participants will remain within the trial, and all data collected up to the point of withdrawal will be retained for the purposes of follow-up and primary outcome data collected and included in the trial analysis.

If a child, or their parents/guardians, no longer wishes to take part or contribute further data to the trial (e.g. follow-up), their decision must be respected. A withdrawal form should be completed and sent to KCTF. Withdrawal of the participant should be recorded in their medical notes and no further data will be collected.

All identifiers will be removed at the end of the trial to ensure anonymity for all participants recruited into COAST.

## 7. Trial treatment

### 7.1 Randomisation allocation and treatment arms

**Figure 4.**
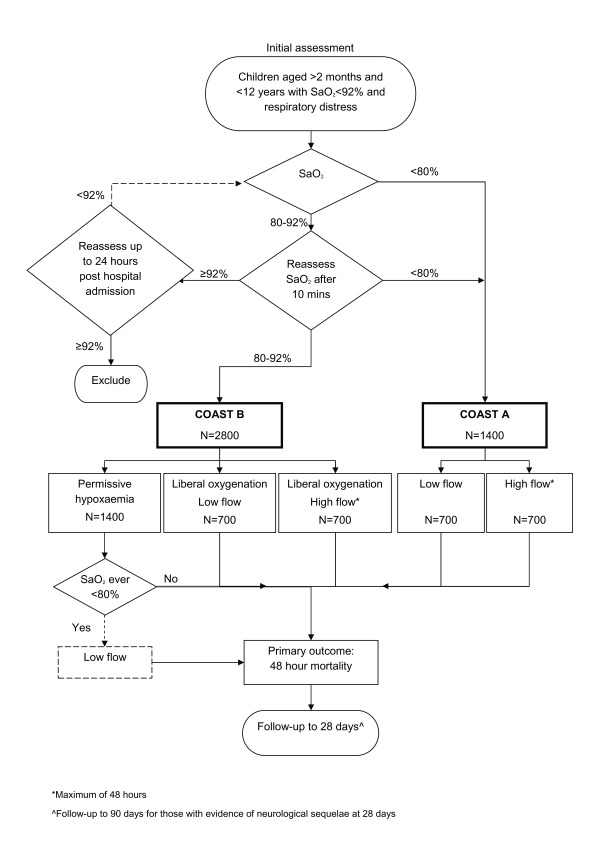
Trial flow chart.

The trial has two strata: **COAST A**: severe hypoxaemia, SpO _2_ <80%); and **COAST B**: hypoxaemia (SpO _2_ ≥80% and <92%). Patients will be assessed on severity of their hypoxaemia (SpO _2_ <92% initially recorded over 5 minutes in children breathing room air) and assigned to their severity stratum ( Figure 4).

**Figure 5.**
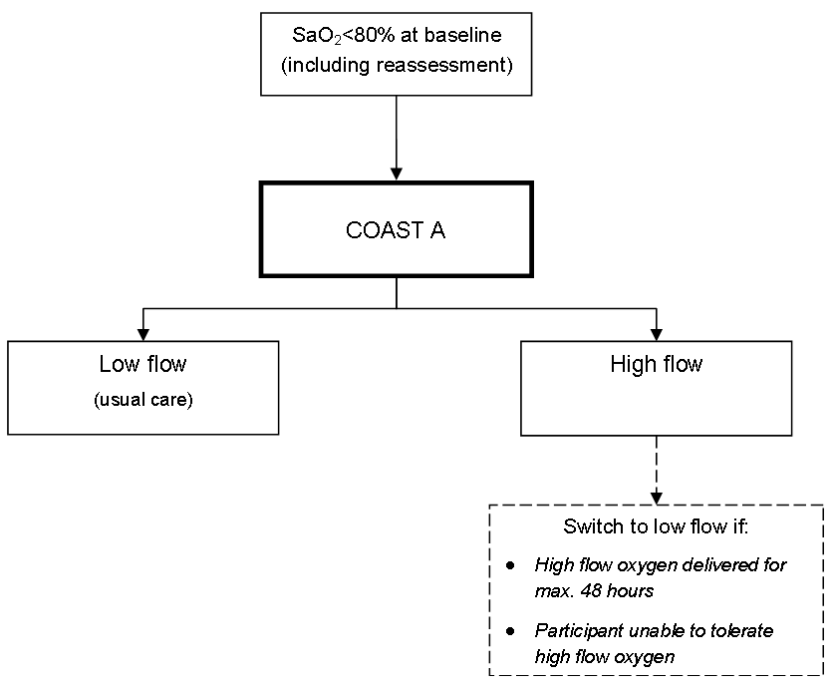
COAST A: Treatment arms.

**If baseline SpO _2_ is below 80% (severe hypoxaemia)**, patients will be enrolled into COAST A ( Figure 5).

**Figure 6.**
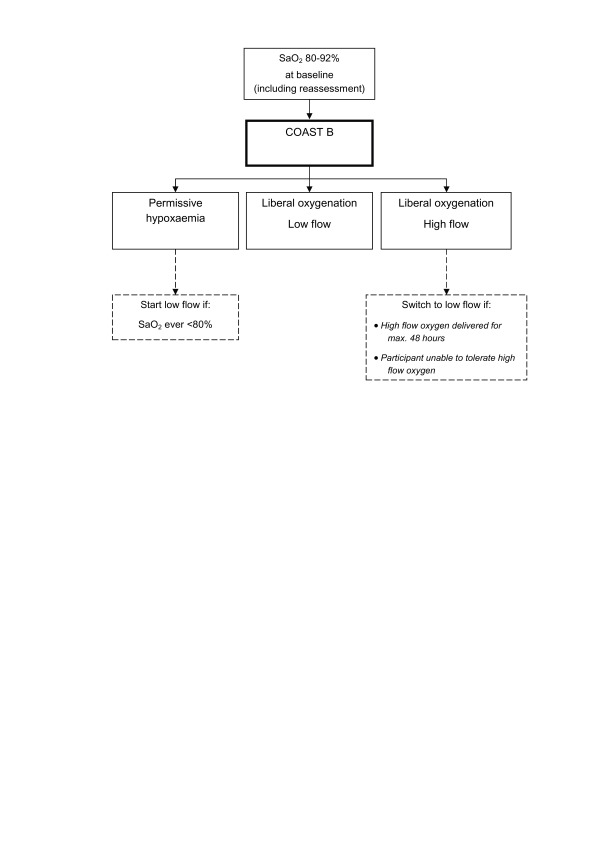
COAST B: Treatment arms.

**If baseline SpO _2_ is between 80–91%, which remains for 10 minutes** (i.e. an additional 5 minutes to initial screening) in children breathing room air (in order to mitigate against the possibility of a transient hypoxaemia or development of severe hypoxaemia), patients will be enrolled into COAST B ( Figure 6).

For children after 10 minutes reassessment, SpO _2_:

- drops below 80%, patients will be eligible for COAST A and randomised **accordingly**;
- if ≥92% at admission patients will remain eligible for up to 24 hours post hospital admission (so long as the patient has not received oxygen). After 24 hours patients are no longer eligible, irrespective of SpO _2_.

The reassessments conducted within the 24-hour time window will prevent recruiting patients with only transient episodes of hypoxaemia (e.g. accompanying hypoglycaemia or convulsions which resolved on appropriate treatment) and allow for recruitment of children who subsequently develop hypoxaemia early into their hospital admission.

Usual emergency care will be provided as part of standard clinical management.

**Trial treatments:**

i. Low flow oxygen until oxygen saturation ≥92% in room air
ii. High flow oxygen by AirVo _2_^TM^ until oxygen saturation ≥92% in room air, or *the participant will switch to low flow oxygen (usual care) if:*
  • *High flow oxygen delivered for a maximum of 48 hours; if oxygen is still required at this point then the child will be switched to oxygen delivery by low flow (i.e. standard of care)*
  • *Unable to tolerate high flow oxygen provided indices of ventilation are optimal*

Extra clinical personnel, regular patient clinical assessment and the basic equipment for continuous patient monitoring will be available during the trial so that if complications were to arise they could be detected early and treated. Pre-trial training will include sign recognition for these complications and training delivery of treatment.

All trial participants will receive standard of care, including antibiotics (intravenous or oral) and anti-malarial drugs following national guidelines, based on WHO syndromic patient management ^27^.

### 7.2 Duration of treatment

The trial treatment period will last for a maximum of 48 hours post-randomisation. After this time point, the participant will switch to usual care (standard clinical management).

For children receiving oxygen delivered by high flow oxygen, at 48 hours, if oxygen is still required (i.e. failure to wean into room air) at this point, then the child will be switched to oxygen delivery by low flow (i.e. standard of care).

During the 0–48 hour period, if a child is unable to tolerate high flow oxygen and if oxygen is still required (i.e. failure to wean into room air), then the child will be switched to oxygen delivery by low flow (i.e. standard of care).

If oxygen is discontinued before 48 hours e.g. hypoxaemia is resolved (SpO _2_≥92% measured continuously over 30 minutes ^27^), then they will switch to usual care (standard clinical management) following a successful trial of oxygen weaning.

Once weaning has occurred further clinical and bedside monitoring will occur twice daily until hospital discharge.

***7.2.1 Standard clinical management.*** All other care will be determined by the clinical team primarily responsible for the participant’s care.

### 7.3 Trial equipment

Reliable sources of oxygen and bedside equipment for patient monitoring will be provided for the duration of the trial.

High flow oxygen will be delivered by the AirVO _2_^TM^ device; AirVO _2_^TM^ is manufactured by industry leaders Fisher & Paykel Healthcare. The device, complete with consumables, will be provided to each participating site. The AirVO _2_^TM^ device can be used from bottled oxygen and has an inflow for concentrator-delivered supplemental oxygen. The device is already adaptable for children over a wide weight and age range and is capable of providing sustainable, non-invasive respiratory support generating high flows through a self-contained turbine-driven device. Medical and nursing staff will be able to operate the machine with minimal training. Fisher & Paykel will also provide technical support during the trial. The AirVO _2_^TM^ device will only be used for patients randomised to receive high flow oxygen delivery.

In addition, oxygen saturation measurement will be undertaken by pulse oximeters. Up to 10 pulse oximeters will be provided per site. The pulse oximeter is a handheld device for use in the operating room, recovery unit and ward areas. It is both battery powered (3 AA alkaline batteries) or mains powered (nickel-metal hydride rechargeable battery or a lithium ion rechargeable battery). The standard battery use is approximately 14 hours.

## 8. Data collection

### 8.1 Data collection – participants ( Table 2)

***8.1.1 Baseline – characteristics.*** Following consent and randomisation(s), data will be collected on:

- date and time of hospital admission
- eligibility criteria
- date and time of consent
- patient identifiers
- patient sociodemographics
- oxygen saturation level
- pre-existing neurocognitive deficits
- hospital admission diagnosis and category
- suspected cause of respiratory distress
- severity of dyspnoea (assessed using an age-adjusted integrated respiratory distress and wheeze score ^68^)
- co-morbidities
- severity of illness score
- child’s ability to tolerate oxygen using an analogue scale ^69^

**Table 2.** Participant data collection schedule.

	TRIAL PERIOD											
	Enrolment and Allocation	Post-allocation										
TIMEPOINT	*Day of* *admission*	Hour 0	*Hour 1*	*Hour 2*	*Hour 4*	*Hour 8*	*Hour 12*	*Hour 24*	*Hour 36*	*Hour 48*	*Hospital* *discharge*	*Day 28*
ENROLMENT:												
Eligibility screening	X											
Informed consent	X *											
Allocation		X										
ASSESSMENTS:												
*Baseline data*	X	X										
*Diagnostic* *investigations*	X											X
*Laboratory/point of* *care diagnostics*	X											
*Hospital stay data*		X	X	X	X	X	X	X		X	X	
*Primary outcome*										X		
*Secondary* *outcomes*										X	X	X
*Cost-effectiveness* *outcomes*											X	X

*as soon as feasible

***8.1.2 Baseline – laboratory tests.*** Admission blood samples will be taken for the following investigations:

- full blood count, urea and electrolytes
- lactate
- glucose
- malaria blood slide and/or malaria RDT
- white cell and red cell pellet (for subsequent DNA extraction)
- blood culture (where culture facilities are available)
- nasal and oropharyngeal swabs and saliva specimen (for pathogen diagnosis)
- plasma storage (acute serology; markers of immune markers)
- EDTA x 2: for haemoglobinopathies and molecular pathogen diagnostics

Chest x-rays will also be taken when the patient is stable. In accordance with national guidelines, HIV testing will be performed after admission procedures are complete and assent given by parents or guardians. Pre- and post-test counselling will be done in accordance to routine practice

***8.1.3 Hospital stay.*** All participants will be reassessed clinically at 1, 2, 4, 8, 12, 24, 36 and 48 hours post-randomisation, and twice daily thereafter until discharged from hospital. At each review, the following will be recorded:

- conscious level
- vital signs (heart rate, oxygen saturation and percentage, respiratory rate, axillary temperature, blood pressure)
- severity of dyspnoea (assessed using an age-adjusted integrated respiratory distress and wheeze score ^68^)
- oxygen received and delivery method
- prescribed drugs received
- volume and type of intravenous fluid (including blood transfusion) per 24 hours
- AEs

***8.1.4 Hospital stay – additional laboratory tests.*** Additional blood samples will be taken at the discretion of the clinical team at other times during the hospital stay if deemed necessary for a clinical deterioration for the following investigations:

- haemoglobin
- blood glucose
- lactate

***8.1.5 Hospital discharge***

- final diagnosis
- discharge status
- discharge date and time

All clinical staff members must follow all national and international guidance on hospital discharge tests and procedures ^27^.

Children, or their parents/guardians, will receive a follow-up clinic visit invitation on a card; the date will be 28 days after the day of randomisation. Transport costs after discharge and for the follow-up visit will be reimbursed to parents/guardians by the trial site coordinator. Children who are still an in-patient at 28 days will be followed up in hospital.

***8.1.6 Follow-up.*** A symptom checklist and targeted physical examination will be performed at the clinic visit scheduled for 28 days after the day of randomisation.

- Medical history since last visit, including hospital re-admissions, transfusions and SAEs
- Convalescent sampling (plasma)
- Neurocognitive function assessment (using the Developmental Milestones Checklist and an adaptation of the Kilifi Developmental Inventory ^70, 71^)

Any participant lost to follow-up before 28 days will be traced for vital status (using locator data and mobile telephone contacts taken prior to discharge).

Participants with evidence of neurocognitive sequelae at 28 days will also be followed up again at 90 days.

***8.1.7 Health economics.*** The trial will measure healthcare-related costs for the trial participants, starting at randomisation and continuing for the duration of follow-up.

- Costs incurred by the participants and their families (e.g. transport, indirect and companion person’s costs)
- Information on hospitalisation (e.g. number, reason, and duration of stay)
- Data on other healthcare resource utilisation (e.g. outpatient visits, medications, and *procedures*)

***8.1.8 Outcomes***

**Primary outcome**

Survival status will be recorded at 48 hours post-randomisation. Any participants lost to follow-up before 48 hours, without withdrawing consent, will be traced for vital status.

**Secondary outcomes**

- Treatment failure will be defined as continued SpO _2_ <92% in the presence of respiratory distress (indrawing or intercostal retractions) at 48 hours post-randomisation.
- Survival status will be recorded at 28 days post-randomisation. Any participants lost to follow-up before 28 days, without withdrawing consent, will be traced for vital status.
- Neurocognitive sequelae will be defined as the development of a new neurocognitive deficit, assessed using the modified Kilifi Developmental Index, between the day of admission and 28 days following randomisation.
- Composite disability-free survival will be defined as a change to disability-free status, between the day of admission and 28 days following randomisation.
- Time to hypoxaemia resolution will be defined as the duration in hours from randomisation to hypoxaemia resolution, defined as SpO _2_ ≥92% measured continuously over a period of 30 minutes during initial hospital stay.
- Duration of respiratory support will be defined as the number of days alive and free from receipt of administered oxygen during the first 28 days following randomisation.
- Length of initial hospital stay will be defined as the duration in days from randomisation to initial hospital discharge.
- Re-admission to hospital will be defined as hospitalisation (at the trial centre or other health facility) for acute illness (i.e. not due to an existing illness or an elective admission) between discharge and 28 days following randomisation.
- Anthropometric status will be defined by Z-scores of height, weight and mid-upper arm circumference and any evidence of visible severe wasting or signs of kwashiorkor.
- Resolution of neurocognitive sequelae will be defined as the full resolution by Day 90 of any neurocognitive deficit reported at Day 28.
- Resource use and costs will be assessed using the methods previously described (see *section 8.1.7*) at 28 days following randomisation.

### 8.2 Data collection – site staff

***8.2.1 Nurses.*** Nurses will be responsible for collecting data collected at each clinical review during the hospital stay.

***8.2.2 Doctors.*** During the initial hospital stay, a doctor will review the participant routinely (at the time points listed above) using a symptom checklist and targeted physical examination (to evaluate any reported symptoms). Additional reviews will be done where clinically indicated and recorded in the case notes.

***8.2.3 Trial site coordinator.*** The trial site coordinator will be responsible for completing the Case Report Forms (CRF), following up any data queries and tracing the results of pending laboratory tests.

## 9. Data management

Each site will be responsible for its own data entry using the COAST CRF ( Supplementary File 3). After 48 hours, the hospital patient’s notes will be used in place of the CRF; a COAST trial number sticker will be added on the front page of the patient’s hospital notes and a caption “COAST trial participant: Notes to be reviewed at discharge” written below the sticker. Additional pages in the COAST CRF will be available to report key clinical, laboratory or AEs and bi-daily observations during the period prior to discharge.

Following on-site monitoring, data will be entered onto a web-enabled trial database directly at the site. The site will retain the original paper CRFs. All clinical and laboratory data will be recorded in the CRF and stored with a unique trial number identifier. All data will be regularly backed up and backup copies will be stored securely both on and off site.

Data will be checked and undergo validation checks for completeness, accuracy and consistency of data. Data queries that arise from these checks will be sent from KCTF to the trial site coordinators. The trial site coordinator is required to ensure that queries are resolved as soon as possible, including updating the relevant paper CRFs and the trial database as required. The KCTF will send reminders for any overdue data or outstanding queries. Following validation, data will be remotely extracted by ICNARC CTU once a month to generate progress reports for each site.

Ongoing data entry and validation and adherence to the trial protocol at sites will be closely monitored by KCTF; any concerns will be raised with the site PI.

### 9.1 Sample storage

All samples for storage (including those taken during hospital stay and those taken for research purposes, if consented to) will require no more than 10 ml of venous blood (varies by age). Any blood taken for research purposes under emergency deferred consent from children whose parents subsequently refuse consent will be confidentially destroyed.

## 10. Safety monitoring

### 10.1 Definitions

The following definitions have been adapted from Directive 2001/20/EC of the European Parliament (Clinical Trials Directive) ^72^ and ICH-GCP guidelines (E6(R1), 1996).

Adverse event (AE) – Any untoward medical occurrence or effect in a patient participating in a trial, which does not necessarily have a causal relationship with the trial treatment. An AE can therefore be any unfavourable symptom or disease temporally associated with the use of the trial treatment, whether or not it is related to the allocated trial treatment.

Serious adverse event (SAE) – An AE is defined as serious if it:

- results in death;
- is life-threatening;
- requires inpatient hospitalisation or prolongation of existing hospitalisation;
- results in persistent or significant disability/incapacity; or
- is a congenital anomaly/birth defect.

Medical judgement should be exercised in deciding whether an SAE is serious in other situations. Important SAEs that are not immediately life-threatening, do not result in death or hospitalisation, but may jeopardise the subject or require intervention to prevent one or any of the other outcomes listed in the definition above should also be considered as serious.

Life threatening in the definition of an SAE refers to an event in which the subject was at risk of death at the time of the event. It does not refer to an event that hypothetically might have caused death if it were more severe.

Unexpected and related serious adverse event - A suspected AE related to the treatment that is both unexpected (i.e. not consistent with the expected outcomes of the treatment being offered) and serious.

*N.B. Suspected Unexpected Serious Adverse Reaction will not be assessed in this trial as it falls outside the scope of the European Union Clinical Trial Directive.*

### 10.2 Severity

The assessment of severity will be graded based on the PI’s or a delegated local investigator’s clinical judgement using the following definitions:

- None: indicates no event or complication.
- Mild: complication results in only temporary harm and does not require clinical treatment.
- Moderate: complication requires clinical treatment but does not result in significant prolongation of hospital stay. Does not usually result in permanent harm and where this does occur the harm does not cause functional limitation to the participant.
- Severe: complication requires clinical treatment and results in significant prolongation of hospital stay and/or permanent functional limitation.
- Life threatening: complication may lead to death.
- Fatal: indicates that the participant died as a direct result of the complication/AE.

### 10.3 Relatedness

The PI or designee should use clinical judgement to determine the relationship between the trial treatment and the occurrence of each AE using the following definitions:

- None: there is no evidence of any relationship to the trial treatment.
- Unlikely: there is little evidence to suggest a relationship to the trial treatment, and there is another reasonable explanation of the event.
- Possibly: there is some evidence to suggest a relationship to the trial treatment, although the influence of other factors may have contributed to the event.
- Probably: there is probable evidence to suggest a relationship to the trial treatment, and the influence of other factors is unlikely.
- Definitely: there is clear evidence to suggest a relationship to the trial treatment, and other possible contributing factors can be ruled out.

### 10.4 Expectedness

The PI or designee must assess the expectedness for each SAE regardless of its relationship to the trial procedures.

- Expected: the event is listed as an expected AE (see below)
- Unexpected: the event is not listed as an expected AE below.

Expected AEs that could be observed in participants up to 28 days following randomisation:

- Nasal trauma
- Facial trauma
- Pneumothorax
- Subcutaneous emphysema
- Aspiration

Note that this list is not extensive, other AE should be reported as described in Section 10.6.

### 10.5 Exempt adverse events

All patients eligible for COAST are critically ill and due to the complexity of their condition are at increased risk of experiencing AEs. Many of these events are expected as a result of the patient’s medical condition and standard treatment received in hospital, but may not be related to participation in the trial. Consequently, any unexpected AEs occurring as a result of the patient’s medical condition or standard hospital treatment will not be reported. Pre-existing conditions do not qualify as AEs unless they worsen, but should be documented in the patient’s medical notes.

### 10.6 Recording and reporting

All AEs that occur between randomisation and 28 days post-randomisation must be recorded in the patient medical notes, on the COAST paper CRFs and on the web-enabled trial database. Information regarding date and time of event onset, severity and relatedness of the AE to trial treatment must be recorded.

At each clinical review the clinician or nurse will check for potential SAEs, grade 3 or 4 AEs and solicited AEs. SAEs will be reported to KCTF using an SAE form; the form should be completed, scanned and sent electronically within 24 hours of becoming aware of the event. The clinician or nurse should record the nature of event, date of onset, severity, corrective therapies given, outcome, relatedness and expectedness on the SAE form, and the PI or designee should sign it off prior to sending. Sites should also report all SAEs as required by their local REC and/or local policies. Any questions concerning AE recording and reporting should be directed to KCTF in the first instance via email or by telephone.

All SAEs must be followed-up until resolution. The PI or designee must provide a follow-up SAE report(s) within five days of the initial report if the SAE was not resolved at the time the initial report was submitted.

### 10.7 Central processing

SAEs will be reviewed immediately by a designated physician (SAE reviewer) in the KCTF and periodically by the ERC. If the event is evaluated by either the PI or the SAE reviewer as an unexpected and related SAE, the KCTF will submit a report to the appropriate ethics committees within 15 calendar days.

The KCTF will provide safety information to the ChI, TMG, Trial Steering Committee (TSC) and Data Monitoring Committee (DMC) for review on a regular basis (as deemed necessary).

### 10.8 Additional monitoring and notification process

The KCTF will also monitor data for documented AEs that are not considered to be related to the trial treatment. In the event that any trial procedure does appear to be resulting in AEs, the ChI and/or TMG will be contacted for their opinion. If it is declared necessary to review the conduct of the trial, the KCTF will inform the appropriate ethics committees as necessary.

## 11. Trial monitoring and oversight

### 11.1 Site

This trial will be monitored according to a Monitoring and Quality Management Plan, which will set out the frequency of visits, the degree of source document verification against the CRFs and the requirements for triggered on-site monitoring visits. Monitoring will start with 100% source document verification, then will be reviewed for each site once a satisfactory and sustained performance in quality assurance is established. All monitoring will adhere to the ICH-GCP guidelines (E6(R1), 1996).

A detailed site initiation visit (SIV) will be performed at each site by staff from KCTF. The SIV will include training in the trial procedures, such as delivering the trial treatments, reporting guidelines for AEs and data collection and management. All staff at sites involved in the trial will receive formal training in GCP through a dedicated training programme during the SIV and through an on-line course.

Local monitoring teams, responsible to KCTF, will oversee the standards and quality of the trial at each site. All monitors will be appropriately qualified and trained.

At each monitoring visit, monitors will:

- verify completeness of Investigator Site File;
- assess for any non-adherence to protocol;
- review eligibility verification and consent procedures;
- look for missed AE recording/reporting;
- verify completeness, consistency and accuracy of data being entered on CRFs; and
- provide additional training as needed.

The monitors will require access to all patient medical records, including, but not limited to, laboratory test results and prescriptions. The PI or delegated local investigator should work with the monitor to ensure that any problems detected are resolved.

### 11.2 Site trial management team

Each site will have a PI, who will have overall responsibility for the day-to-day management of the trial, and will employ a clinical team to coordinate triage and identification of eligible participants and administration of trial interventions. Additional clinical nurses will be responsible for clinical reviews and follow-up. A clinical nurse coordinator will manage the trial team and integrate the trial within routine clinical services and patient care.

Trial site coordinators are responsible for ensuring completeness of trial documents, SAE reporting and interactions with monitors and KCTF. Support will be provided for laboratory staff and ward assistants to facilitate blood tests, sample preparation and storage. At each site, dedicated COAST administrators will manage trial operations and human resource issues.

Overall trial management, training and monitoring and data management will be coordinated from the KCTF, based in Kenya. The ICNARC CTU, based in London, will oversee trial management by teleconferences and face-to-face meetings with the TMG.

### 11.3 Trial management group

The TMG will be comprised of the ChI, site PIs and co-investigators. The TMG will meet approximately once a year in-person and will hold regular monthly teleconferences to discuss general trial matters. In addition, the site PIs will summarise their progress and discuss any challenges and difficulties at their site. All decisions regarding the overall running of the trial will be made in this forum, with the exception of matters of fundamental importance to the viability of the trial or that require major changes to the protocol – these will be referred to the TSC.

### 11.4 Trial steering committee

The progress of the trial will be monitored and supervised by the TSC. It will meet approximately once a year. The TSC will compose of an independent chair (Professor Elizabeth Molyneux, OBE former Head of Paediatrics Queen Elizabeth Hospital, Blantyre; Founder of ETAT; and member of a number of WHO guideline committees) and a majority (60%) of independent members (composed in experts in international child health and intensive care). Non-independent members will include the ChI and representatives from the co-investigators and from each participating country. Representatives from the Funder and Sponsor will be invited to the meetings as observers.

### 11.5 Data monitoring committee

An independent DMC will be set up to review data on enrolment, safety, adherence to the trial protocol and efficacy at regular intervals and in strict confidence. It will meet approximately once a year. The DMC will report to the TSC and to the Ethics Committee in each country, if, in their view, the data provide proof beyond reasonable doubt that one of the allocated strategies is better than its comparator in terms of the primary outcome. The TSC will then decide whether to amend (which may include removing one of the intervention arms) or stop the trial before the end of the planned follow-up. The DMC will be comprised of a chair, Professor Timothy Peto, a statistician and three other independent members (see Supplementary File 4: DMC Charter).

### 11.6 Endpoint review committee

An ERC will be set up to review clinical data and will determine the validity of the endpoints. The ERC will adjudicate endpoints blinded to randomised allocations; relationship to all possible trial treatments (liberal oxygenation, permissive hypoxaemia and high and low flow oxygen delivery) will be solicited to avoid unblinding. The ERC will be made up of an independent chair (Dr Jennifer Evans) and will include the PIs from each site as well as other independent clinicians.

## 12. Trial closure

### 12.1 End of trial

The end of the trial will be when the final participant has completed their 28-day follow-up (or 90-day follow-up if applicable). The Declaration of End of Trial Form will then be submitted to the relevant ethics committee(s), as required.

### 12.2 Archiving

At the end of the trial, the KCTF will securely archive all central and site essential trial-related documentation and samples taken for a minimum of 10 years, in line with Medical Research Council (MRC) guidance on Good Research Practice ^73^, and thereafter confidentially destroyed. All archived documents must be available for inspection and monitoring by appropriate authorities upon request.

### 12.3 Early discontinuation

Should a site choose to close to participant recruitment before the end of the trial, the PI must inform the KCTF in writing. Follow-up, as per the trial protocol, must continue for all participants already recruited into the trial at that site. Sites that contravene the trial protocol and the Site Agreement will be subject to review by the TMG and Sponsor.

The trial may be stopped early by the TSC. In which case, sites will be informed in writing by the KCTF of the reasons for early closure and the actions to be taken as regards the treatment of participants. All randomised participants will continue to be followed up as per the trial protocol.

## 13. Statistics

A Trial Statistician will be based at ICNARC CTU, under the supervision of Dr David Harrison (Senior Statistician, ICNARC CTU), and will provide statistical support for DMC analyses.

### 13.1 Sample size calculation

Sample size calculations were informed by identifying patients meeting the proposed COAST inclusion criteria within two datasets: the FEAST trial (n=873/3170; 28%); and the Kilifi Hospital, Kenya admission cohort (n=2609/36,621; 7%). Based on these data, it was estimated that two thirds of eligible children would present with SpO _2_ ≥80% (FEAST 63%; Kilifi 69%). Baseline 48-hour mortality for children receiving low flow oxygen was assumed to be 9% for children with SpO _2_ ≥80% (FEAST 10%; Kilifi 9%) and 26% with SpO _2_<80% (FEAST 30%; Kilifi 26%). Due to the complex nature of the design, power calculations were undertaken by simulating datasets under the assumed alternative hypotheses and calculating the proportion of simulated datasets in which a significant effect (P<0.05) was detected for each of the two comparisons ^74^. Based on these simulations, a total sample size of 4,200 children would give 90% power to detect a clinically relevant difference of a 33% RR reduction associated with liberal oxygenation compared with permissive hypoxaemia, and a clinically relevant difference of a 25% RR reduction for high flow compared with low flow oxygen delivery.

The sample size calculation is based on the primary outcome of mortality at 48 hours with no losses to follow-up expected. Losses to follow-up are anticipated to increase to 2% at 28 days post-randomisation, based on data from the FEAST trial ^24^.

### 13.2 Analysis plan

The analyses will be described in detail in a full Statistical Analysis Plan. This section summarises the main issues.

All analyses will be undertaken using the intention-to-treat principle with a two-sided P-value of P<0.05 taken to indicate statistical significance.

The primary outcome (48-hour mortality) will be analysed as a binary outcome using logistic regression including both treatment allocation variables simultaneously and adjusted for the stratifying factors (baseline SpO _2_ and trial site).

Secondary outcomes will be analysed using generalised linear models, with the same model structure as for the primary outcome above, as follows:

- Treatment failure at 48 hours, re-admission to hospital by 28 days, neurocognitive sequelae at 28 days and disability-free survival to 28 days will be analysed as binary outcomes using logistic regression, with all deaths included in the failure group.
- Time to hypoxaemia resolution will be analysed as a time-to-event outcome using a Fine and Gray competing risks model to account for the competing risk of mortality.
- Length of initial hospital stay, days alive and free from receipt of oxygen and anthropometric status will be analysed as continuous outcomes using linear regression.
- Survival to 28 days will be analysed as a time-to-event outcome using a Cox proportional hazards model.
- Resolution of neurocognitive sequelae at 90 days will be analysed as a binary outcome using logistic regression, only among those with neurocognitive sequelae at 28 days.

Pre-specified subgroup analyses will assess whether the effects of the two interventions vary according to categories of baseline SpO _2_ (<80%, 80–84.9%, 85–89.9%, 90–91.9%) and by trial site. Additional hypothesis-generating analyses will investigate whether there is any evidence for a different impact of the interventions according to the following categorical variables: fever; malaria; microbiological evidence of sepsis (blood culture or retrospective molecular diagnosis); radiographic evidence of pneumonia; HIV; severe anaemia (haemoglobin <5g/dl); and undiagnosed sickle cell disease. Subgroup analyses will be conducted by testing the significance of interaction terms in the regression models as specified above.

### 13.3 Interim analysis

The DMC will meet to review unblinded data for at least 525, 1575 and 2625 participants (corresponding to 3 months, 9 months and 15 months at the anticipated recruitment rate). Guidelines to recommend early termination will be based on a Peto-Haybittle stopping rule (P<0.001). A recommendation to discontinue recruitment, in all participants or in selected subgroups, will be made only if the results are likely to convince the general clinical community and participants in COAST.

## 14. Ancillary studies

### 14.1 Economic and cost-effectiveness evaluation

The cost-effectiveness of oxygen delivery strategies will be estimated by comparing clinical outcomes and costs for participants receiving each of the trial treatments. Resource utilisation data and unit cost data (e.g. basic costs, literature and other health-economic data) will be collected as part of the trial dataset and also collated based on datasets collected in other trials of acutely sick children.

### 14.2 Molecular diagnostics

Two molecular methods are being used increasingly in research and clinical practice to identify bacteria. 16S ribosomal deoxyribonucleic acid (16S rDNA), common to all species of bacteria, can be detected with a broad-range polymerase chain reaction (PCR); specific quantitative PCR (qPCR) can also be used to quantify the 16S rDNA subunit to measure directly the number of bacteria. However broad range 16S rDNA PCR is subject to artefact from endogenous and exogenous bacterial products ^75– 77^ and therefore without either sequencing the PCR product, or carrying out more sensitive qPCR, there is concern that changes in the qPCR may not be due to circulating organisms. Unfortunately, sequencing the 16S rDNA has so far yielded results compatible with environmental contamination rather than recognised gut commensals. The use of specific primers renders qPCR less vulnerable to background contaminants than broad-range 16S rDNA PCR, and therefore more sensitive ^78, 79^. The disadvantage of qPCR is the need to predict which bacterial species are likely to be relevant.

The goal of this study would be to identify the role of bacteria in the aetiology of lethal pneumonia in African children. Previous studies have shown comparable results between frozen EDTA plasma and whole blood, and so we would assay standard 16S rDNA PCR, and a panel of 10 qPCR reactions (including Enterobacteriaceae, a panel of anaerobes, Streptococcus pneumoniae, Staphylococcus aureus, group A streptococcus).

White and red cell pellets collected into a 2ml EDTA bottle taken at enrolment, (stored at -80°C) and plasma will be stored and shipped at the conclusion of the study to Kilifi, Kenya and molecular diagnostics will compare the range of pathogens identified in cases (deaths) and controls (survivors) frequency matched by age group, study site and season.

### 14.3 Investigation of respiratory viruses

Respiratory viruses (RVs) are a major cause of acute lower respiratory tract infections worldwide ^80^. However, in developing countries the potential role of RVs in life-threatening disease and mortality remains uncertain, e.g. influenza ^81^. The present trial, in which samples will be collected from children with a high mortality risk, provides an important opportunity to elucidate this role. Furthermore, data are scarce on the occurrence of RVs in paediatric pneumonia cases in Uganda or Kenya, and almost nothing is known for Central and Eastern Africa on the origins and spread of these viruses. Nasal pharyngeal/oro-pharyngeal swabs samples will be stored and batched transferred to Professor Nokes viral pathogen laboratory in Kilifi, Kenya. Ribonucleic acid (RNA) extracted from NP/OP swabs will be screened for a broad range of RVs, including influenza viruses, Respiratory Syncytial Virus (RSV), coronaviruses and rhinoviruses using a multiplex real time PCR assay system ^82^. Partial and/or whole genome sequencing of the virus positive samples, for example RSV ^83^, and influenza ^84^, amongst other respiratory targets will be undertaken.

The outcomes of this study will be:

(i) the prevalence and seasonal patterns of a range of respiratory viruses in children with life threatening features of respiratory disease;
(ii) estimates of the risk of death in RV positive relative to negative children; and
(ii) information on the sources of viruses and patterns of spread in East Africa.

### 14.4 Short period incidence study of severe acute respiratory infection (sprint-sari)

SPRINT-SARI is a global multi-network, multicentre, prospective, short period incidence observational study of all severe SARI patients admitted to the in-patient unit of interest (ICU or hospital ward), during a defined 5-or 7-day study period once annually. The Chief investigator is Professor Steven Webb, Clinical Professor, University of Western Australia, Royal Perth Hospital. The primary aim of this study is to establish a research response capability for future epidemics / pandemics through a global SARI observational study. The secondary aim of this study is to describe the clinical epidemiology and microbiology profiles of patients with SARI. Since children with severe pneumonia will be enrolled across 5 sites (2 countries) in COAST, this will act as an opportunity to provide data to this global platform. Only basic clinical and some diagnostic data will be provided from each site together with outcome data. There will be no linking of the data to the trial intervention and only fully anonymised and de-identified data will be submitted to the centrally coordinated database for SPRINT-SARI.

### 14.5 Sickle cell disease status

The mortality rate for children with sickle cell disease (SCD) admitted to hospital with triage features, fulfilling severe and very severe pneumonia, is three-fold high compared to children without SCD (Professor Thomas N. Williams, unpublished). The reasons for high mortality in this group remains unclear and whether this may indicate a diagnosis of ‘chest syndrome’ or a much higher risk of bacterial infection including gram negative organisms. As sickle cell status is likely to influence the outcome in this study, we aim to test all children for this common condition in Africa (with SCD and sickle cell trait being present in 2% and 7% of children with SP and VSP).

White and red cell pellets collected into a 2ml EDTA bottle (see *section 14.2*) will be stored at -80°C and shipped at the conclusion of the study to Professor Williams haemoglobinopathy laboratory (Kilifi, Kenya). We will extract a sample of DNA from this sample from study participants to describe the distribution of HbSS using PCR. Patients found to be positive for HbSS will be recalled for counselling and for confirmatory testing and if confirmed to be suffering from HbSS they will be encouraged to attend the outpatient clinic for regular treatment.

## 15. Ethical compliance

### 15.1 Trial registration

This trial has been registered with the ISRCTN Registry ( **ISRCTN15622505**).

### 15.2 Central ethical compliance

Favourable ethics opinion has been obtained from the Imperial College REC. This trial has been submitted and received approval by the relevant RECs/Institutional Review Boards and by required regulatory authorities in all participating countries.

The trial will be conducted in accordance with the recommendations for research on human subjects in the Declaration of Helsinki ^85^, the ICH-GCP guidelines (E6(R1), 1996) and the applicable national regulations.

### 15.3 Local ethical compliance

It is the responsibility of the PI to obtain the necessary local approvals and provide them to the KCTF prior to site activation.

COAST will only be conducted at sites where all necessary local approvals for the trial have been obtained and a Site Agreement between the Hospital (site) and the Imperial College London (Sponsor) has been signed.

### 15.4 Confidentiality and data protection

Participants’ identification data will be collected as part of the trial follow-up procedures. The KCTF and the ICNARC CTU will preserve the confidentiality of participants taking part, which will comply with requirements for data protection in the countries where the research is being conducted. All data will be anonymised prior to presentation or publication of any results.

### 15.5 Patient, carer and public involvement and engagement

Each site would either use their existing Community Advisory Board (CAB) or form a specific patient liaison group to feedback concerns and questions from the community and hear about the latest developments in the trial and the wider scientific community, where possible.

Each of the country PIs have discussed the trial with their local hospital CABs (if in existence) and informed Ministry of Health about the trial. Dialogue with these groups will be maintained through regular briefing meetings during the course of the trial and will be a standing item at each TSC meeting in order to rapidly facilitate dissemination and implementation of results. Policy briefs and presentations at national meetings will be used to disseminate results to policy makers.

### 15.6 Declaration of interests

All trial investigators have confirmed that they do not have any financial or other conflicts of interest to declare in relation to this trial.

## 16. Sponsorship and funding

### 16.1 Sponsorship

Imperial College London will act as Sponsor for this trial and delegate the responsibility of overseeing the implementation of the trial to KCTF and the ICNARC CTU. Imperial College London holds negligent harm and non-negligent harm insurance policies, which apply to this trial.

### 16.2 Funding

The trial is supported by grant funding from the UK MRC, the UK Department for International Development (DFID) and the Wellcome Trust through the Joint Global Health Trials scheme. The trial is also supported by Fisher & Paykel Healthcare, who is providing equipment and technical support.

A written agreement with the site PI and/or the PI’s institution and Imperial College London will outline the funding arrangements to sites. The TSC will meet and review the financial aspects of the trial at least annually and report to the Sponsor.

## 17. Dissemination policy

All publications and presentations relating to the trial will be authorised by the TMG. The first publication of the trial results will be in the name of the TMG, if this does not conflict with the journal’s policy. If there are named authors, these will include the ChI, Trial Statistician and Trial Manager. Members of the TMG, TSC and DMC and other contributors will be cited by name, if this does not conflict with the journal’s policy. Authorship of sub studies initiated outside of the TMG will be according to the individuals involved in the project but must acknowledge the contribution of the TMG and KCTF.

The TSC is the custodian of the data and specimens generated from the trial; trial data are not the property of individual participating investigators or health care facilities where the data were generated.

During the course and following completion of the trial there will be publications, including manuscripts and abstracts for presentations at national and international meetings, as well as the preparation of manuscripts for peer-reviewed publication. In order to avoid disputes regarding authorship, a consensus approach will be established that will provide a framework for all publications derived in full or in part from this trial. Authorship criteria will be determined using the guidelines provided by The International Committee of Medical Journal Editors. The following approach is derived from the Lancet and from the publication policies used in other MRC clinical trials:

- All publications are to be approved by the TMG and TSC before submission for publication. Any publication arising before the end of the trial will also be approved by the DMC in order to ensure that the primary objective of the trial is not compromised. In particular, no analyses by randomised group of any outcome (primary, secondary or other) in either the main trial or associated sub studies will be conducted or presented before the end of the trial, other than those for interim review by the DMC. The TMG and TSC will resolve problems of authorship and maintain the quality of publications.
- Manuscripts arising from the trial will, wherever possible, be submitted to peer-reviewed journals that enable Open Access via UK PubMed Central within six months of the official date of final publication. All conference presentations will be made available as soon as possible after the event via the COAST website. All publications will acknowledge the trial's funding sources.
- For all publications, the TMG will nominate a chairperson or approve an individual’s request to chair a manuscript writing committee. The chair will usually be the primary or senior author. The chairperson is responsible for identifying fellow authors and for determining the order of authorship that will appear on the manuscript. The TSC will resolve any problems of authorship and maintain the quality of publications.
- The TMG will maintain a list of investigators to be presented in the acknowledgements at the end of the paper. This list will include investigators who contributed to the investigation being reported but who are not members of the writing committee. In principle, sub study reports should include all investigators for the main trial, although in some instances, where a smaller number of investigators have made any form of contribution, it may be appropriate to abbreviate the listing.
- All headline authors in any publication arising from the main trial or sub studies must have a made a significant academic or project management contribution to the work that is being presented. “Significant” must be defined by a written declaration of exactly what the contribution of any individual is believed to have been. In addition to fulfilling the criteria based on contribution, additional features that will be considered in selecting an authorship group will include the recruitment of participants who contributed data to any set of analyses contained in the manuscript, the conduct of analyses (laboratory and statistical) and leadership and coordination of the project in the absence of a clear academic contribution.
- The data derived from this trial are considered the property of the TSC. The presentation or publication of any data collected by the participating investigators on participants recruited into this trial is under the direct control of the TMG and TSC (and the DMC before the end of the trial). This is true whether the publication or presentation is concerned directly with the results of the trial or is associated with the trial in some other way. However, although individual participating investigators will not have any inherent right to perform analyses or interpretations, to make public presentations or seek publication of any of the data other than under the auspices of and with the approval of the TMG and TSC (and the DMC before the end of the trial), they will be encouraged to develop sub studies or propose analyses subject to the approval by the TMG and TSC (and the DMC before the end of the trial). Any requests for access to raw data will be welcomed as long as they are scientifically valid and do not conflict with the integrity of the trial or ongoing analyses by the trial team.

Outcome data by randomised group will not be revealed to the participating investigators until the data collection phase and primary full analysis of the trial has been completed. This policy safeguards against possible bias affecting the data collection. The DMC will monitor the outcome results and may recommend that the trial be stopped for safety reasons or if a definitive answer is reached earlier than the scheduled end of the trial.

## 18. Discussion

Systematic and policy reviews have indicated the need for formal evaluation of the hypoxaemia threshold for which oxygen should be targeted and how best to administer oxygen; thus COAST was designed in response. The trial is pragmatic, enrolling children presenting with respiratory symptoms and hypoxaemia, and thus will include large subgroups of children with pneumonia, malaria and other critical illness. This will ensure that the results are applicable to health services in Africa that typically have little or no access to specialist respiratory support facilities. The trial was designed to address the poor outcomes of children in sub-Saharan Africa, which are associated with high rates of in-hospital mortality, 9–10% (for those with oxygen saturations between 80% and 92%) and 26–30% case fatality for those with oxygen saturations <80%, indicating that the current recommendations and/or management strategies are not working in practice. COAST trial will target children, but not newborns, as this group require a separate trial designed to address a different set of objectives and interventions. The COAST trial aims to provide the relevant evidence for policy makers and health services by evaluating key elements of an integrated management strategy in African hospitals on the basis of clinical effectiveness and costs. The health economics component is vital to ensuring that we provide evidence not just on effectiveness, but also cost-effectiveness, to allow policymakers to make decisions on how best to allocate scarce health resources.

## 19. Trial status

The trial started enrolment in February 2017 and to date (17 ^th^ December 2017) has enrolled 907 participants, with one interim analysis by the DMC. The trial will contribute new knowledge to the relevant academic disciplines, including opportunities for additional sub-studies to increase our knowledge and understanding of host response to oxygenation strategies.

**Table:** 

Protocol versions			
Date	Amendment no.	Protocol Title	Changes
25 ^th^ January 2016		1.0	
7 ^th^ July 2016	1st	2.0	Relocation of trial site from Kinshasa to Mombasa
11 ^th^ January 2017	2nd	2.1	Minor amendments and clarifications

## Ethics approvals

ICREC (Imperial College): 15IC3100; SOMREC (Uganda): 2016-030; SERU (Kenya): C/0053/3300

## Supplementary material

Supplementary File 1: Patient information sheet and consent.

Supplementary File 2: Verbal assent form.

Supplementary File 3: Case Report Forms.

Supplementary File 4: Data Monitoring Committee (DMC) Charter.

## References

[1] Black RE Cousens S Johnson HL: Global, regional, and national causes of child mortality in 2008: a systematic analysis. *Lancet.* 2010;375(9730):1969–87. 10.1016/S0140-6736(10)60549-1 20466419

[2] Rudan I Boschi-Pinto C Biloglav Z: Epidemiology and etiology of childhood pneumonia. *Bull World Health Organ.* 2008;86(5):408–16. 10.2471/BLT.07.048769 18545744 PMC2647437

[3] Rajaratnam JK Marcus JR Flaxman AD: Neonatal, postneonatal, childhood, and under-5 mortality for 187 countries, 1970–2010: a systematic analysis of progress towards Millennium Development Goal 4. *Lancet.* 2010;375(9730):1988–2008. 10.1016/S0140-6736(10)60703-9 20546887

[4] Hospital Care for Children: Guidelines for the management of common childhood illnesses with limited resources. Geneva: World Health Organization;2005 Reference Source 24006557

[5] Mwaniki MK Nokes DJ Ignas J: Emergency triage assessment for hypoxaemia in neonates and young children in a Kenyan hospital: an observational study. *Bull World Health Organ.* 2009;87(4):263–70. 10.2471/BLT.07.049148 19551234 PMC2672576

[6] Banajeh SM: Outcome for children under 5 years hospitalized with severe acute lower respiratory tract infections in Yemen: a 5 year experience. *J Trop Pediatr.* 1998;44(6):343–6. 10.1093/tropej/44.6.343 9972077

[7] Moïsi JC Gatakaa H Berkley JA: Excess child mortality after discharge from hospital in Kilifi, Kenya: a retrospective cohort analysis. *Bull World Health Organ.* 2011;89(10):725–32, 32A. 10.2471/BLT.11.089235 22084510 PMC3209982

[8] Ngari MM Fegan G Mwangome MK: Mortality after Inpatient Treatment for Severe Pneumonia in Children: a Cohort Study. *Paediatr Perinat Epidemiol.* 2017;31(3):233–242, in press. 10.1111/ppe.12348 28317139 PMC5434848

[9] Subhi R Adamson M Campbell H: The prevalence of hypoxaemia among ill children in developing countries: a systematic review. *Lancet Infect Dis.* 2009;9(4):219–27. 10.1016/S1473-3099(09)70071-4 19324294

[10] Duke T Peel D Wandi F: Oxygen supplies for hospitals in Papua New Guinea: a comparison of the feasibility and cost-effectiveness of methods for different settings. *P N G Med J.* 2010;53(3–4):126–38. 23163183

[11] Belle J Cohen H Shindo N: Influenza preparedness in low-resource settings: a look at oxygen delivery in 12 African countries. *J Infect Dev Ctries.* 2010;4(7):419–24. 10.3855/jidc.859 20818088

[12] Recommendations for management of common childhood conditions: evidence for technical update of pocket book recommendations. Geneva: World Health Organization;2012 Reference Source 23720866

[13] Rojas-Reyes MX Granados Rugeles C Charry-Anzola LP: Oxygen therapy for lower respiratory tract infections in children between 3 months and 15 years of age. *Cochrane Database Syst Rev.* 2009; (1):CD005975. 10.1002/14651858.CD005975.pub2 25493690 PMC6464960

[14] Hammitt LL Kazungu S Morpeth SC: A preliminary study of pneumonia etiology among hospitalized children in Kenya. *Clin Infect Dis.* 2012;54(Suppl 2):S190–9. 10.1093/cid/cir1071 22403235 PMC3297554

[15] Graham SM English M Hazir T: Challenges to improving case management of childhood pneumonia at health facilities in resource-limited settings. *Bull World Health Organ.* 2008;86(5):349–55. 10.2471/BLT.07.048512 18545737 PMC2647436

[16] Duke T Wandi F Jonathan M: Improved oxygen systems for childhood pneumonia: a multihospital effectiveness study in Papua New Guinea. *Lancet.* 2008;372(9646):1328–33. 10.1016/S0140-6736(08)61164-2 18708248

[17] English M Gathara D Mwinga S: Adoption of recommended practices and basic technologies in a low-income setting. *Arch Dis Child.* 2014;99(5):452–6. 10.1136/archdischild-2013-305561 24482351 PMC3995214

[18] Gilbert-Kawai ET Mitchell K Martin D: Permissive hypoxaemia versus normoxaemia for mechanically ventilated critically ill patients. *Cochrane Database Syst Rev.* 2014; (5):CD009931. 10.1002/14651858.CD009931.pub2 24801519 PMC6465096

[19] Martin DS Grocott MP: Oxygen therapy in critical illness: precise control of arterial oxygenation and permissive hypoxemia. *Crit Care Med.* 2013;41(2):423–32. 10.1097/CCM.0b013e31826a44f6 23263574

[20] Bradley BD Howie SR Chan TC: Estimating oxygen needs for childhood pneumonia in developing country health systems: a new model for expecting the unexpected. *PLoS One.* 2014;9(2):e89872. 10.1371/journal.pone.0089872 24587089 PMC3930752

[21] Djelantik IG Gessner BD Sutanto A: Case fatality proportions and predictive factors for mortality among children hospitalized with severe pneumonia in a rural developing country setting. *J Trop Pediatr.* 2003;49(6):327–32. 10.1093/tropej/49.6.327 14725409

[22] Onyango FE Steinhoff MC Wafula EM: Hypoxaemia in young Kenyan children with acute lower respiratory infection. *BMJ.* 1993;306(6878):612–5. 10.1136/bmj.306.6878.612 8369033 PMC1676956

[23] George EC Walker AS Kiguli S: Predicting mortality in sick African children: the FEAST Paediatric Emergency Triage (PET) Score. *BMC Med.* 2015;13(1):174. 10.1186/s12916-015-0407-3 26228245 PMC4521500

[24] Maitland K Kiguli S Opoka RO: Mortality after fluid bolus in African children with severe infection. *N Engl J Med.* 2011;364(26):2483–95. 10.1056/NEJMoa1101549 21615299

[25] Magree HC Russell FM Sa'aga R: Chest X-ray-confirmed pneumonia in children in Fiji. *Bull World Health Organ.* 2005;83(6):427–33. 15976893 PMC2626254

[26] Cunningham S Rodriguez A Adams T: Oxygen saturation targets in infants with bronchiolitis (BIDS): a double-blind, randomised, equivalence trial. *Lancet.* 2015;386(9998):1041–8. 10.1016/S0140-6736(15)00163-4 26382998 PMC4673090

[27] WHO Guidelines Approved by the Guidelines Review Committee: Pocket Book of Hospital Care for Children: Guidelines for the management of common childhood illnesses. Geneva: World Health Organization;2013; Report No.: 978 92 4 154837 3. 24006557

[28] Dobson MB: Oxygen concentrators and cylinders. *Int J Tuberc Lung Dis.* 2001;5(6):520–3. 11409577

[29] Hill SE Njie O Sanneh M: Oxygen for treatment of severe pneumonia in The Gambia, West Africa: a situational analysis. *Int J Tuberc Lung Dis.* 2009;13(5):587–93. 19383191

[30] Matai S Peel D Wandi F: Implementing an oxygen programme in hospitals in Papua New Guinea. *Ann Trop Paediatr.* 2008;28(1):71–8. 10.1179/146532808X270716 18318953

[31] La Vincente SF Peel D Carai S: The functioning of oxygen concentrators in resource-limited settings: a situation assessment in two countries. *Int J Tuberc Lung Dis.* 2011;15(5):693–9. 10.5588/ijtld.10.0544 21756524

[32] English M Esamai F Wasunna A: Delivery of paediatric care at the first-referral level in Kenya. *Lancet.* 2004;364(9445):1622–9. 10.1016/S0140-6736(04)17318-2 15519635

[33] Catto AG Zgaga L Theodoratou E: An evaluation of oxygen systems for treatment of childhood pneumonia. *BMC Public Health.* 2011;11(Suppl 3):S28. 10.1186/1471-2458-11-S3-S28 21501446 PMC3231901

[34] Rojas-Reyes MX Granados Rugeles C Charry-Anzola LP: Oxygen therapy for lower respiratory tract infections in children between 3 months and 15 years of age. *Cochrane Database Syst Rev.* 2014; (12):CD005975. 10.1002/14651858.CD005975.pub3 25493690 PMC6464960

[35] Smyth A Carty H Hart CA: Clinical predictors of hypoxaemia in children with pneumonia. *Ann Trop Paediatr.* 1998;18(1):31–40. 10.1080/02724936.1998.11747923 9691999

[36] Shann F Dobson M Peel D: Oxygen therapy for acute respiratory infections in young children in developing countries. Geneva: World Health Organization;1993 Reference Source

[37] Jackson RM: Pulmonary oxygen toxicity. *Chest.* 1985;88(6):900–5. 10.1378/chest.88.6.900 3905287

[38] Hayes RA Shekar K Fraser JF: Hyperoxic damage and the need for optimised oxygenation practices. *Crit Care.* 2013;17(4):441. 10.1186/cc12802 23890474 PMC4056610

[39] Davis PG Tan A O'Donnell CP: Resuscitation of newborn infants with 100% oxygen or air: a systematic review and meta-analysis. *Lancet.* 2004;364(9442):1329–33. 10.1016/S0140-6736(04)17189-4 15474135

[40] Munkeby BH Børke WB Bjørnland K: Resuscitation with 100% O _2_ increases cerebral injury in hypoxemic piglets. *Pediatr Res.* 2004;56(5):783–90. 10.1203/01.PDR.0000141988.89820.E3 15347772

[41] Tan A Schulze A O'Donnell CP: Air versus oxygen for resuscitation of infants at birth. *Cochrane Database Syst Rev.* 2005; (2):CD002273. 10.1002/14651858.CD002273.pub3 15846632 PMC7017642

[42] Cabello JB Burls A Emparanza JI: Oxygen therapy for acute myocardial infarction. *Cochrane Database Syst Rev.* 2010; (6):CD007160. 10.1002/14651858.CD007160.pub2 20556775

[43] Ronning OM Guldvog B: Should stroke victims routinely receive supplemental oxygen? A quasi-randomized controlled trial. *Stroke.* 1999;30(10):2033–7. 10.1161/01.STR.30.10.2033 10512903

[44] Perrin K Wijesinghe M Healy B: Randomised controlled trial of high concentration versus titrated oxygen therapy in severe exacerbations of asthma. *Thorax.* 2011;66(11):937–41. 10.1136/thx.2010.155259 21597111

[45] Mikkelsen ME Christie JD Lanken PN: The adult respiratory distress syndrome cognitive outcomes study: long-term neuropsychological function in survivors of acute lung injury. *Am J Respir Crit Care Med.* 2012;185(12):1307–15. 10.1164/rccm.201111-2025OC 22492988 PMC3381234

[46] Bass JL Corwin M Gozal D: The effect of chronic or intermittent hypoxaemia on cognition in childhood: a review of the evidence. *Pediatrics.* 2004;114(3):805–16. 10.1542/peds.2004-0227 15342857

[47] Abdelsalam M Cheifetz IM: Goal-directed therapy for severely hypoxic patients with acute respiratory distress syndrome: permissive hypoxemia. *Respir Care.* 2010;55(11):1483–90. 20979676

[48] Cheifetz IM Hamel DS: Is permissive hypoxemia a beneficial strategy for pediatric acute lung injury? *Respir Care Clin N Am.* 2006;12(3):359–69,v–vi. 16952798 10.1016/j.rcc.2006.06.003

[49] Frey B McQuillan PJ Shann F: Nasopharyngeal oxygen therapy produces positive end-expiratory pressure in infants. *Eur J Pediatr.* 2001;160(9):556–60. 10.1007/s004310100798 11585079

[50] Frey B Shann F: Oxygen administration in infants. *Arch Dis Child Fetal Neonatal Ed.* 2003;88(2):F84–8. 10.1136/fn.88.2.F84 12598492 PMC1721518

[51] Benaron DA Benitz WE: Maximizing the stability of oxygen delivered via nasal cannula. *Arch Pediatr Adolesc Med.* 1994;148(3):294–300. 10.1001/archpedi.1994.02170030064015 8130865

[52] SUPPORT Study Group of the Eunice Kennedy Shriver NICHD Neonatal Research Network, Carlo WA Finer NN: Target ranges of oxygen saturation in extremely preterm infants. *N Engl J Med.* 2010;362(21):1959–69. 10.1056/NEJMoa0911781 20472937 PMC2891970

[53] Chisti MJ Salam MA Smith JH: Bubble continuous positive airway pressure for children with severe pneumonia and hypoxaemia in Bangladesh: an open, randomised controlled trial. *Lancet.* 2015;386(9998):1057–65. 10.1016/S0140-6736(15)60249-5 26296950

[54] Collins CL Holberton JR Barfield C: A randomized controlled trial to compare heated humidified high-flow nasal cannulae with nasal continuous positive airway pressure postextubation in premature infants. *J Pediatr.* 2013;162(5):949–54.e1. 10.1016/j.jpeds.2012.11.016 23260098

[55] Dysart K Miller TL Wolfson MR: Research in high flow therapy: mechanisms of action. *Respir Med.* 2009;103(10):1400–5. 10.1016/j.rmed.2009.04.007 19467849

[56] Mayfield S Jauncey-Cooke J Hough JL: High-flow nasal cannula therapy for respiratory support in children. *Cochrane Database Syst Rev.* 2014; (3): CD009850. 10.1002/14651858.CD009850.pub2 24604698 PMC6516984

[57] Spence KL Murphy D Kilian C: High-flow nasal cannula as a device to provide continuous positive airway pressure in infants. *J Perinatol.* 2007;27(12):772–5. 10.1038/sj.jp.7211828 17762844

[58] Corley A Caruana LR Barnett AG: Oxygen delivery through high-flow nasal cannulae increase end-expiratory lung volume and reduce respiratory rate in post-cardiac surgical patients. *Br J Anaesth.* 2011;107(6):998–1004. 10.1093/bja/aer265 21908497

[59] Roca O Riera J Torres F: High-flow oxygen therapy in acute respiratory failure. *Respir Care.* 2010;55(4):408–13. 20406507

[60] Lee JH Rehder KJ Williford L: Use of high flow nasal cannula in critically ill infants, children, and adults: a critical review of the literature. *Intensive Care Med.* 2013;39(2):247–57. 10.1007/s00134-012-2743-5 23143331

[61] Schibler A Pham TM Dunster KR: Reduced intubation rates for infants after introduction of high-flow nasal prong oxygen delivery. *Intensive Care Med.* 2011;37(5):847–52. 10.1007/s00134-011-2177-5 21369809

[62] Bassler D Montori VM Briel M: Early stopping of randomized clinical trials for overt efficacy is problematic. *J Clin Epidemiol.* 2008;61(3):241–6. 10.1016/j.jclinepi.2007.07.016 18226746

[63] Pocock SJ: When (not) to stop a clinical trial for benefit. *JAMA.* 2005;294(17):2228–30. 10.1001/jama.294.17.2228 16264167

[64] Shann F Lange T: Bubble CPAP for pneumonia: perils of stopping trials early. *Lancet.* 2015;386(9998):1020–2. 10.1016/S0140-6736(15)60691-2 26311458

[65] Peto R Pike MC Armitage P: Design and analysis of randomized clinical trials requiring prolonged observation of each patient. I. Introduction and design. *Br J Cancer.* 1976;34(6):585–612. 10.1038/bjc.1976.220 795448 PMC2025229

[66] Howitt P Darzi A Yang GZ: Technologies for global health. *Lancet.* 2012;380(9840):507–35. 10.1016/S0140-6736(12)61127-1 22857974

[67] Maitland K Molyneux S Boga M: Use of deferred consent for severely ill children in a multi-centre phase III trial. *Trials.* 2011;12:90. 10.1186/1745-6215-12-90 21453454 PMC3077324

[68] Liu LL Gallaher MM Davis RL: Use of a respiratory clinical score among different providers. *Pediatr Pulmonol.* 2004;37(3):243–8. 10.1002/ppul.10425 14966818

[69] van Dijk M de Boer JB Koot HM: The reliability and validity of the COMFORT scale as a postoperative pain instrument in 0 to 3-year-old infants. *Pain.* 2000;84(2–3):367–77. 10.1016/S0304-3959(99)00239-0 10666543

[70] Abubakar A Holding P Van de Vijver F: Developmental monitoring using caregiver reports in a resource-limited setting: the case of Kilifi, Kenya. *Acta Paediatr.* 2010;99(2):291–7. 10.1111/j.1651-2227.2009.01561.x 20353499 PMC2814084

[71] Abubakar A Holding P van Baar A: Monitoring psychomotor development in a resource-limited setting: an evaluation of the Kilifi Developmental Inventory. *Ann Trop Paediatr.* 2008;28(3):217–26. 10.1179/146532808X335679 18727851 PMC3908377

[72] Union EPaCoE: Directive 2001/20/EC of European Parliament and of the Council of 4 April 2001 on the approximation of the laws, regulations and administrative provisions of the Member States relating to the implementation of good clinical practice in the conduct of clinical trials on medicinal products for human use. *Official Journal L121.* 2001 Reference Source 16276663

[73] Ranchord AM Perrin K Weatherall M: A randomised controlled trial of the effect of high concentration oxygen on myocardial ischaemia during exercise. *Int J Cardiol.* 2012;160(3):201–5. 10.1016/j.ijcard.2011.04.017 21570139

[74] Landau S Stahl D: Sample size and power calculations for medical studies by simulation when closed form expressions are not available. *Stat Methods Med Res.* 2013;22(3):324–45, in press. 10.1177/0962280212439578 22491174

[75] Harris KA Hartley JC: Development of broad-range 16S rDNA PCR for use in the routine diagnostic clinical microbiology service. *J Med Microbiol.* 2003;52(Pt 8):685–91. 10.1099/jmm.0.05213-0 12867563

[76] Millar BC Xu J Moore JE: Risk assessment models and contamination management: implications for broad-range ribosomal DNA PCR as a diagnostic tool in medical bacteriology. *J Clin Microbiol.* 2002;40(5):1575–80. 10.1128/JCM.40.5.1575-1580.2002 11980924 PMC130933

[77] Ferri E Novati S Casiraghi M: Plasma levels of bacterial DNA in HIV infection: the limits of quantitative polymerase chain reaction. *J Infect Dis.* 2010;202(1):176–7; author reply 178. 10.1086/653215 20518669

[78] Marchetti G Bellistrì GM Borghi E: Microbial translocation is associated with sustained failure in CD4+ T-cell reconstitution in HIV-infected patients on long-term highly active antiretroviral therapy. *AIDS.* 2008;22(15):2035–8. 10.1097/QAD.0b013e3283112d29 18784466

[79] Merlini E Bai F Bellistrì GM: Evidence for polymicrobic flora translocating in peripheral blood of HIV-infected patients with poor immune response to antiretroviral therapy. *PLoS One.* 2011;6(4):e18580. 10.1371/journal.pone.0018580 21494598 PMC3073938

[80] Shi T McLean K Campbell H: Aetiological role of common respiratory viruses in acute lower respiratory infections in children under five years: A systematic review and meta-analysis. *J Glob Health.* 2015;5(1):010408. 10.7189/jogh.05.010408 26445672 PMC4593292

[81] Nair H Brooks WA Katz M: Global burden of respiratory infections due to seasonal influenza in young children: a systematic review and meta-analysis. *Lancet.* 2011;378(9807):1917–30. 10.1016/S0140-6736(11)61051-9 22078723

[82] Hammitt LL Kazungu S Welch S: Added value of an oropharyngeal swab in detection of viruses in children hospitalized with lower respiratory tract infection. *J Clin Microbiol.* 2011;49(6):2318–20. 10.1128/JCM.02605-10 21490188 PMC3122752

[83] Agoti CN Otieno JR Munywoki PK: Local evolutionary patterns of human respiratory syncytial virus derived from whole-genome sequencing. *J Virol.* 2015;89(7):3444–54. 10.1128/JVI.03391-14 25609811 PMC4403408

[84] Baillie GJ Galiano M Agapow PM: Evolutionary dynamics of local pandemic H1N1/2009 influenza virus lineages revealed by whole-genome analysis. *J Virol.* 2012;86(1):11–8. 10.1128/JVI.05347-11 22013031 PMC3255882

[85] Declaration of Helsinki - Ethical Principles for Medical Research Involving Human Subjects. Helsinki;1964 Reference Source 19886379

