# Supplementary material for: Children’s Oxygen Administration Strategies Trial (COAST): A randomised controlled trial of high flow versus oxygen versus control in African children with severe pneumonia
Source: Wellcome Open Res. 2018 Jan 9;2:100. Originally published 2017 Oct 11. [Version 2] doi: 10.12688/wellcomeopenres.12747.2 (PMC5771148; doi:10.12688/wellcomeopenres.12747.2)
Supplement: Supplementary file 1 [file wellcomeopenres-2-14819-s0000.tgz › 91a4a8c1-684d-4f1e-858f-4fd2c3601960.pdf]

<Insert HOSPITAL HEADER>

Children's Oxygen Administration Strategies Trial (COAST)

Information for Parents and Carers

Introduction

We are inviting your child to take part in a research study called COAST. It is being conducted at five hospitals in Africa and will include 4,200 infants and children aged between 28 days to 12 years. Before you decide if you want your child to take part, it is important for you to understand why the research is being done and what it will involve. Please take time to read this information sheet carefully or ask someone to read it to you. Please discuss this with the doctors or nurses and ask questions if there is anything that is not clear or if you would like more information. Joining the COAST study is entirely voluntary. Take time to decide whether or not you wish your child to take part.

B: Study purpose: What is the reason for doing the COAST study?

Your child has been admitted to hospital because they have symptoms of a chest infection which is causing them to breath faster and with extra effort. The small instrument with a band/clip around your child's finger (pulse oximeter: [*show*]) found that the oxygen level in your child's blood is lower than normal– this indicates that the lungs/chest may not be working properly. This would normally be treated, if available, by giving your child oxygen. However, we don't know whether the oxygen treatment we give your child helps them get better and we also don't know how best to give this treatment – either the flow rate should be fast or slow. So, the COAST study is trying to find out the best treatment to help your child breathe more easily and improve the way their lungs are working – this may or may not involve giving your child oxygen.

C: Study Procedures: What will it involve for my child?
What treatments will he/she be given?

Your child will be given all the usual treatment for their illness, according to standard <COUNTRY> health guidelines. If you agree for your child to take part in this study, they will receive treatment based on how low the oxygen level is in their blood:

If their oxygen level is very low, your child will receive oxygen that will be given by one of two different flow rates:

- At a lower flow rate from a tube connected to an oxygen cylinder that will deliver oxygen by mask or through some soft prongs into your child's nose
- At a higher flow rate which delivers oxygen/air (which is warmed and humidified) from a cylinder at a higher flow to soft prongs into your child's nose

If your child oxygen level is moderately low, your child will receive oxygen given in two different ways (*as explained above or can be repeated*) or not receive oxygen immediately.

- At a lower flow rate from a tube connected to an oxygen cylinder that will deliver oxygen by mask or through some soft prongs into your child's nose
- At a higher flow rate which delivers oxygen/air (which is warmed and humidified) from a cylinder at a higher flow to soft prongs into your child's nose

- If your children receives no immediate oxygen we will monitor your child's oxygen levels very closely to see if they get better without the need for oxygen. However, if the oxygen level drops to a very low level we will provide oxygen treatment given at a lower flow rate.

Once they start treatment, the doctors/nurses will study the effect on the child's breathing and oxygen levels. The doctors and nurses will take extra and regular measurements at the bedside to help us find out if the child is getting better or not. All the instruments and devices used in this study are very safe, and have been used in lots of other children in many areas of the world. Even though they are unlikely to cause your child any harm or discomfort we will monitor them closely for any adverse effects.

Study Procedures

1. We will do some initial blood tests to check how sick your child is and some additional ones to see if they are recovering or not. We should be able to discover these during these regular checks and treat them promptly. At the time of hospital admission this will involve 5-8mls (one to two teaspoons depending on the size of your child) plus 2mls extra which will be saved for future tests to help us find out why your child became ill. You will get some of the results back during the study – others will be done much later at the end of the study and you may not get the results of some of these tests. After the initial blood tests, we will only use blood tests which are taken from the child's fingertip, which will help us adjust other treatments that your child may need (i.e. a transfusion or some extra sugar).

2. As explained above your child will be carefully monitored and checked during their time in hospital. If they respond well to the treatment, we may stop the oxygen/air and

observe over a time period to see if they fully recover – they will continue with their other usual treatments. If your child if they do not respond well, for example do not like the high flow oxygen then we will switch the flow rate to low flow oxygen. If your child is not receiving oxygen and their oxygen levels drop then we will then start oxygen by low flow as is usual standard of care.

3. When your child is ready to go home, we will ask you to come back in one month for a health check. We will check where you live and take your contact details, to help us find you if you are not able to come back. You will have our details so that you can contact us if you have any concerns about your child's condition or if they go back to hospital – and our team will call you back if necessary. If they have any illnesses, we will treat these or refer them to another specialist if necessary. Some of the blood taken at this visit will be stored, approximately 2-3ml (equivalent to half a teaspoon) to check your child's haemoglobin level and do a malaria test and to store some blood for research into why your child became sick.
4. Some of the tests to find out what caused your child's illness are needed as part of this research cannot be done in this country at the moment, so part of the samples will be sent to laboratories overseas. This will involve a small portion of the blood that was taken during the study, which we will store. Some of the tests we will do will look at whether your child has a trait or characteristic that they inherited from their parents that will make them more vulnerable to severe illness. This is called genetic research. Individual names will be removed and will be replaced by codes, so that information cannot be linked to participants. Future research done on these samples will be approved by a national independent expert committee, to ensure that participants' safety and rights are respected.

D: Risks of study participation

There are very few risks to your child being in this study. If for any reason the doctor thinks that it is not in your child's best interest to be in the study then they will not be enrolled in the study but will be given their usual treatment. You do not have to pay anything to join the study.

If your child does not respond to the treatment, then we may increase the rate of flow or amount of oxygen they receive until we see signs on the monitors that they are getting better. If they do not respond (i.e. they are still working very hard to breathe), the doctors/nurses will discuss with you whether they may need longer for the treatment to work and/or whether you would prefer that your child switches to the usual standard of care, which may involve oxygen delivery at a lower rate.

All children will have blood taken as part of this study. Many of the tests that will be done in hospital would also be done if you chose not to join the study. However, we will take a small amount of extra blood at the follow-up visit(s) – see above.

E: Benefits of study participation

Your child will get no direct benefits from this study. However, your child will get close observation during the study, and by taking part your child may help us improve the care of children who have breathing difficulties in the future. Regular assessment of your child by doctors/nurses will enable us to make important changes to your child's treatment in hospital, if these are needed. We will help supply routine medical supplies and treatments for your child to the hospital, so that you will not have to buy any treatments. This will mean that there will be no delay to starting treatment for your child. The medical tests we perform during this illness will also be paid for by the study.

You will be asked to bring your child back for follow up visit(s), and we will pay for your transport from hospital to your home and back to the clinic so you can attend this important visit. During the follow up visit(s), we will treat any illnesses we find, or arrange referral to appropriate clinic or hospital.

F: Alternatives to study participation: What will happen if I don't agree to participate?

All participation in research is voluntary. You are free to decide if you want your child to take part or not. Your child will still receive the recommended standard of care treatment if they do not take part. If you do agree to join the study you can change your mind at any time, and can withdraw your child from the research. This will not affect their care now or in the future and not incur any penalties. We hope that if you decide to withdraw later, you would give a reason for your decision.

G: Compensation

You will not incur any costs from participation in this study. All your travel expenses for attending the visits we invite you too will be paid, based on the cost of public transport to and from your home. As well, when you bring your child for follow up, snacks and drinks will be available and for meals, in a situation where you have to wait for a long time before being attended to.

H: Confidentiality. Who will have access to information about me/my child in this research?

All our research records are stored securely in locked cabinets and password protected computers. Only a few people who are working closely on the study will be able to view

information from your child. When we report on the results of the study will not include any private information that will make it possible to identify your child.

I: Study related injury

This research is supported by Imperial College London who holds insurance policies, which apply to this study. If you experience harm or injury as a result of taking part in this study, you will be eligible to claim compensation without having to prove that Imperial College is at fault. Some specific treatment and compensation are not included in our insurance policies and if you want more information about this you should discuss it with your doctor.

J: Contacts and questions

Who has allowed this research to take place?

All research conducted in <COUNTRY> is approved by national independent expert committees to make sure the research is conducted properly and that study participants' safety and rights are respected.

What if I have any questions?

You may ask any of our staff questions at any time. You can also contact those who are responsible for the care of your child and this research:

<NAMED SITE PI AND CONTACT DETAILS >

If you have any questions about your rights as a participant, please contact:

<NAMED SITE PI AND CONTACT DETAILS >

<COUNTRY Ethics Research Board>

<Insert HOSPITAL HEADER>

Children's Oxygenation Administration Strategies Trial (COAST)

Date	DD	MM	YYYY	Patient IP. Number							
Child's Initials				Trial Number							

Please initial (or mark) box if you agree:

I confirm that I have read/been read the Patient Information Sheet (version 1.0) for the COAST trial and that I understand what will be required if my child participates in the trial. The trial has been explained to me and my questions have been answered.	
I understand that my child's participation is voluntary and that I am free to withdraw him or her at any time, without giving any reason, without my medical care or legal rights or my child's medical care or legal rights being affected.	
I understand that sections of any of my child's medical notes may be looked at by responsible individuals involved in the running of the trial or from regulatory authorities where it is relevant to my child's participation in this research. I give permission for these individuals to have access to my child's records, but understand that strict confidentiality will be maintained.	
I understand that my child will be treated by the COAST doctor/nurse, after discharge from hospital, will be followed up at day 28 (and day 90 if needed). After the trial, my child's healthcare will be provided by the national health system.	
I agree to allow blood samples to be taken from my child and for my child's samples to be stored for later testing. I understand that my child and I may not be given the results of tests performed on stored samples.	
I agree to samples being exported overseas for further studies.	
**I agree for my child to participate in the COAST trial.**	

Parent/carer's signature (or thumbprint)	Print name	Date (day/month/year) and Time

Witness's signature (if thumbprint used above)	Print name	Date (day/month/year) and Time

Doctor's signature	Print name	Date (day/month/year) and Time

IMPORTANT: One signed original to be kept in COAST trial file by the researcher, one signed copy to be given to the parent/guardian/carer and one signed copy to be kept in the clinic notes.
