# Supplementary material for: Children’s Oxygen Administration Strategies Trial (COAST): A randomised controlled trial of high flow versus oxygen versus control in African children with severe pneumonia
Source: Wellcome Open Res. 2018 Jan 9;2:100. Originally published 2017 Oct 11. [Version 2] doi: 10.12688/wellcomeopenres.12747.2 (PMC5771148; doi:10.12688/wellcomeopenres.12747.2)
Supplement: Supplementary file 2 [file wellcomeopenres-2-14819-s0001.tgz › 0ddb7819-f391-4ab6-a1d4-92301b61f607.pdf]

Emergency Verbal Assent Form

Children's Oxygenation Administration Strategies Trial (COAST)

Verbal Assent Form

Child's Initials				Male ❍ Female ❍	Date/Year of Birth	D	D	M	M	M	Y	Y	Y	Y	Age (years)		

Date of Form	D	D	M	M	M	2	0	Y	Y	Clinic/Hospital Number								

NOTE: For children who are critically ill and in whom informed consent would lead to significant delay in starting treatment a verbal assent will be obtained by the doctor from the *parent or guardian* after brief discussion with admitting COAST doctor or nurse.

We advise that this should include the following phrases:

- We are going to provide the treatment for your child that is recommended by the government.
- We want to find out if we can improve on these current recommendations by trying new treatments that we think will work better and we do this by research.
- All research is checked by independent committees to make sure that the potential benefits to individuals outweigh the risks. All participation in research is voluntary, and so you can refuse.
- We would like your child to participate in this research for us to learn the best way to treat respiratory distress complicated by hypoxia.
- Do you agree for your child to take part in this research? You can say no and your child will still receive the same level of care with the government's recommended treatment.

Parent/Guardian assents to research?	**Please circle:** Yes / No

Parent or guardian's name	Relationship with child	Time (24 hour clock)			
		H	H	M	M

Doctor or nurse's signature	Print name	Date								
		D	D	M	M	M	2	0	Y	Y

IMPORTANT: One signed original copy to be kept in COAST trial file by the researcher, one signed copy to be given to parent/guardian/carer and one signed copy to be kept in the clinic notes.
