# Supplementary material for: Children’s Oxygen Administration Strategies Trial (COAST): A randomised controlled trial of high flow versus oxygen versus control in African children with severe pneumonia
Source: Wellcome Open Res. 2018 Jan 9;2:100. Originally published 2017 Oct 11. [Version 2] doi: 10.12688/wellcomeopenres.12747.2 (PMC5771148; doi:10.12688/wellcomeopenres.12747.2)
Supplement: Supplementary file 3 [file wellcomeopenres-2-14819-s0002.tgz › 25223385-7653-4066-bdf0-a9b1cf9bf0c6.pdf]

COAST	# Form 1: Screening & eligibility	PLACE STICKER HERE 8-Feb-2017 v. 1.0

Clinic/hospital number: ☐☐☐☐☐☐☐☐☐ **Centre:** ☐ Kilifi ☐ Mombasa ☐ Mulago ☐ Soroti ☐ Mbale

Child's initials: ☐☐☐ **COAST ID:** ☐☐☐☐☐☐ **Child's name:** ____________

A: Screening

i. Date/time of presentation	D D / M M M / 2 0 Y Y H H : M M (24 hour format)
ii. Initial SpO_2 in air (%): ☐☐☐	iii. DoB: D D / M M M / 2 0 Y Y iv. Age: Y Y M M

B: Inclusion criteria

i. Aged 28 days to <12 years:	☐ Yes	☐ No
ii. SpO_2 <92% in air:	☐ Yes	☐ No
iii. Within 24 hours of hospital presentation:	☐ Yes	☐ No
iv. Respiratory illness:	☐ Yes	☐ No
v. Suspected severe pneumonia:	☐ Yes	☐ No

→ **If SpO_2 ≥80% - <92%, take 2nd reading after 5 minutes**

Second SpO_2 in air (%): ☐☐☐

C: Exclusion criteria

i. Received oxygen in the current illness **at another facility:**	☐ Yes	☐ No
ii. Known chronic lung disease (not including asthma):	☐ Yes	☐ No
iii. Known uncorrected cyanotic heart disease:	☐ Yes	☐ No
iv. Previously included in the COAST trial:	☐ Yes	☐ No

Do NOT continue if patient is not eligible

Consent / assent received:	☐ Yes	☐ No
Patient to be randomised to:	☐ **COAST A** (SpO_2 <80%)	☐ **COAST B** (SpO_2 ≥80% - <92%)

D: Physical examination at eligibility

i. Gender: ☐ Male ☐ Female

ii. Weight (kg): ☐☐.☐

iii. Height (cm): ☐☐☐.☐

iv. MUAC (cm): ☐☐.☐

v. Temperature (°C): ☐☐.☐

vi. Temperature gradient: ☐ Yes ☐ No

vii. Central cyanosis: ☐ Yes ☐ No

viii. Responsiveness:

☐ Alert ☐ Pain

☐ Voice ☐ Unresponsive

ix. Fitting: ☐ Yes ☐ No

x. Heart rate (bpm): ☐☐☐

xi. Systolic blood pressure (mmHg): ☐☐☐

xii. Diastolic blood pressure (mmHg): ☐☐☐

xiii. Weak radial pulse: ☐ Yes ☐ No

xiv. Capillary refill time (s): ☐☐

xv. Respiratory rate (bpm): ☐☐☐

xvi. Nasal flaring: ☐ Yes ☐ No

xvii. Audible wheeze (0-3): ☐
0: None, 1: Mild, 2: Moderate (in or out only), 3: Severe (in & out)

xviii. Ability to vocalise (0-3): ☐
0: Normal, 1: Some difficulty, 2: Severe difficulty, 3: Unable

xix. Ability to feed (0-3): ☐
0: Normal, 1: Some difficulty, 2: Severe difficulty, 3: Unable

Completed by: Name	Signature	Date
		D D / M M M / 2 0 Y Y

COAST	Form 2: Consent and randomisation	PLACE STICKER HERE<br>8-Feb-2017 v.1.0

COAST ID: ☐☐☐☐☐☐ **Child's initials:** ☐☐☐ **Clinic/Hospital number:** ☐☐☐☐☐☐☐☐☐

Centre: ☐ Kilifi ☐ Mombasa ☐ Mulago ☐ Soroti ☐ Mbale

A: Consent

i. Date/time of written consent:	D D / M M M / 2 0 Y Y __ __ : __ __ ☐ Not obtained
ii. If not obtained, date/time of verbal assent:	D D / M M M / 2 0 Y Y __ __ : __ __

If only verbal assent obtained, remember to complete written consent as soon as practically possible

B: Randomisation

i. Date/time of randomisation: D D / M M M / 2 0 Y Y   __ __ : __ __

**COAST A**<br>**SpO_2 <80%**	☐ Opti-flow oxygen<br>☐ Low flow oxygen	**COAST B**<br>**SpO_2 ≥80% - <92%**	☐ Opti-flow oxygen<br>☐ Low flow oxygen<br>☐ No oxygen	**COAST ID:** ☐☐☐☐☐

Completed by: Name	Signature	Date
		D D / M M M / 2 0 Y Y

C: Post-randomisation consent

i. If not sought prior to randomisation, written consent obtained:	☐ Yes ☐ No
ii. If yes, date/time of consent:	D D / M M M / 2 0 Y Y __ __ : __ __
iii. If no, reason:	☐ Died before consent obtained ☐ Absconded ☐ Consent declined
iv. Reason consent declined, if provided:	

Completed by: Name	Signature	Date
		D D / M M M / 2 0 Y Y

Form 3: Post-randomisation trial procedures

PLACE STICKER HERE
8-Feb-2017 v.1.0

**COAST ID:**		**Child's initials:**		**Clinic/Hospital number:**	

Centre: ☐ Kilifi ☐ Mombasa ☐ Mulago ☐ Soroti ☐ Mbale

A: Samples

	Sample	Real time test or	Taken at	If delayed, date taken
EDTA (purple top)	2 x 0.5ml	Malaria, FBC, genetics	☐ Yes ☐ No	D D / M M M / 2 0 Y Y
Blood culture (BACTEC)	2-3ml	Microbiology	☐ Yes ☐ No	D D / M M M / 2 0 Y Y
Lithium heparin (green top)	1 x 4ml	Plasma Storage	☐ Yes ☐ No	D D / M M M / 2 0 Y Y
Nasopharyngeal swab	1 tube	Storage	☐ Yes ☐ No	D D / M M M / 2 0 Y Y

B: Point of care tests

Test	Results	Not done	Initials
i. Lactate (mmol/L):	_ _ . _	☐	
ii. Glucose (mmol/L):	_ _ . _	☐	
iii. HIV:	☐ Positive ☐ Negative ☐ Invalid	☐	

C: Chest X-ray

i. Date X-ray requested:	D D / M M M / 2 0 Y Y	☐ Not requested	
ii. Date X-ray completed:	D D / M M M / 2 0 Y Y	☐ Not completed	
iii. Right lung:	☐ Clear	☐ Consolidation (pneumonia)	☐ Other: ______
iv. Left lung:	☐ Clear	☐ Consolidation (pneumonia)	☐ Other: ______

Completed by: Name	Signature	Date
		D D / M M M / 2 0 Y Y

Form 4: Post-randomisation clinical details

8-Feb-2017
v.1.0

COAST ID: ☐☐☐☐☐☐ **Child's initials:** ☐☐☐ **Clinic/Hospital number:** ☐☐☐☐☐☐☐☐☐

Centre: ☐ Kilifi ☐ Mombasa ☐ Mulago ☐ Soroti ☐ Mbale

To be completed within ONE HOUR of randomisation

A. Clinical history of THIS illness	*Please tick ONE box per question*		
	**Yes**	**No**	**Don't Know**
i. History of fever:	☐	☐	☐
ii. If yes, for more than 14 days:	☐	☐	☐
iii. History of cough:	☐	☐	☐
iv. Difficulty breathing:	☐	☐	☐
v. Sore throat:	☐	☐	☐
vi. Earache/ear discharge:	☐	☐	☐
vii. Joint ache:	☐	☐	☐
viii. Vomiting:	☐	☐	☐
ix. Diarrhoea (> 3 loose motions in last 24hours):	☐	☐	☐
x. If yes, bloody:	☐	☐	☐
xi. Haemoglobinuria (red or cola coloured urine):	☐	☐	☐
xii. Fits:	☐	☐	☐
xiii. If yes, lasting more than 30 minutes:	☐	☐	☐

B. Treatment in THIS illness	**Yes**	**No**	**Don't Know**
i. Admitted for over 24 hours in another facility:	☐	☐	☐
ii. Oral antimalarial treatment in the last week:	☐	☐	☐
iii. Injections or infusion of anti-malarials:	☐	☐	☐
iv. Oral antibiotics:	☐	☐	☐
v. Injections of antibiotics:	☐	☐	☐
vi. If yes, which: ______________________			
vii. Inhalers:	☐	☐	☐
viii. Oral steroids:	☐	☐	☐

C. Clinical examination	**Yes**	**No**	**Not Assessed**
i. In-drawing:	☐	☐	☐
ii. Deep breathing:	☐	☐	☐
iii. Grunting:	☐	☐	☐
iv. Crackles /crepitations on auscultation:	☐	☐	☐
v. If yes:	☐ Unilateral		☐ Bilateral
vi. Audible wheeze on auscultation:	☐	☐	☐
vii. Sunken eyes:	☐	☐	☐
viii. Decreased skin turgor:	☐	☐	☐
ix. Cold hands or feet only:	☐	☐	☐
x. Liver size >2cm below costal margin:	☐	☐	☐

COAST ID: | | | | | | **Child's initials:** | | | **Clinic/Hospital number:** | | | | | | | | | |

	Yes	No	Not assessed
xi. Splenomegaly (gross ≥ 5 cm):	☐ Not palpable	☐ Gross	☐ Enlarged
xii. Jaundice:	☐	☐	☐
xiii. Severe pallor	☐	☐	☐
xiv. Very severe wasting/marasmus:	☐	☐	☐
xv. Signs of kwashiorkor:	☐	☐	☐
xvi. Generalised lymphadenopathy:	☐	☐	☐
xvii. Oral candidiasis:	☐	☐	☐
Neurological			
i. Unable to sit unsupported (or breastfeed if ≤6 months):	☐	☐	☐
ii. Coma - unable to localise (or respond to if ≤9 months) painful stimulus	☐	☐	☐
iii. Neck stiffness or bulging fontenelle (infants only):	☐	☐	☐
iv. Evidence of cerebral palsy:	☐	☐	☐
v. Any other major neurological problem: ______________________________			

D. Past history - BEFORE this illness	Yes	No	Don't know
i. Known HIV:	☐	☐	☐
ii. If yes, receiving antiretroviral therapy:	☐	☐	☐
iii. Previous or recent tuberculosis diagnosis:	☐	☐	☐
iv. Known asthma:	☐	☐	☐
v. If yes, regular inhalers:	☐	☐	☐
vi. Two or more hospital admissions in the last year:	☐	☐	☐
vii. Known epilepsy:	☐	☐	☐
viii. Before this illness, child could (circle): Walk unsupported / Sit unsupported / Feed / Suck / None of these			
ix. Parental concerns about child's vision:	☐	☐	☐
x. Parental concerns about child's hearing:	☐	☐	☐

E. About the child's infancy and family	Yes	No	Don't know
i. Gestation at birth:	☐ <37 weeks	☐ ≥37 weeks	☐
ii. Breast fed exclusively for at least 3 months:	☐	☐	☐
iii. Admitted to hospital in first month of life:	☐	☐	☐
iv. Number of siblings:	☐☐		
v. Father's ethnic group and code:	______________________		
vi. Mother's ethnic group and code:	______________________		
vii. Mother attended secondary school:	☐	☐	☐
viii. Parent status:	☐ Both alive	☐ One alive	☐ Both dead
ix. Homestead where child lives:	☐ Urban	☐ Semi-urban	☐ Rural

COAST	# Form 4: Post-randomisation clinical details	8-Feb-2017<br>v.1.0

COAST ID: ☐☐☐☐☐☐ **Child's initials:** ☐☐☐ **Clinic/Hospital number:** ☐☐☐☐☐☐☐☐☐

Centre: ☐ Kilifi ☐ Mombasa ☐ Mulago ☐ Soroti ☐ Mbale

F: Acute diagnosis - *tick all that apply*

☐ LRTI - any	☐ Tuberculosis - any	☐ Urinary tract infection
☐ URTI - any	☐ Hepatitis - any	☐ Recurrent haemoglobinuria
☐ Asthma	☐ Meningitis - any	☐ Dark urine syndrome
☐ Other chest syndrome	☐ HIV / AIDS	☐ Severe anaemia (Hb <6g/dL)
☐ Severe malaria - any	☐ Developmental delay / cerebral palsy	☐ Malnutrition—any
☐ Sepsis/septicaemia	☐ Encephalopathy	☐ Pyrexia of unknown origin
☐ Sickle cell crisis	☐ Osteomylitis / Pyogenic arthritis	☐ Gastroenteritis
☐ Other, please state: ______________________		

G: Presentation

i. First presented at:	☐ This hospital	☐ Level II	☐ Level III
	☐ Level IV	☐ Other district / referral hospital	☐ Private hospital
If **not** this hospital:	ii. Date first presented:	D D / M M M / 2 0 Y Y	H H : M M
	iii. Date referred:	D D / M M M / 2 0 Y Y	H H : M M
	iv. Distance from initial facility to this hospital (km):	☐☐☐	

Completed by: Name	Signature	Date

COAST	Form 5: Oxygen therapy administration	PLACE STICKER HERE<br>23-March-2017 v.1.2
**Centre:** ☐ Kilifi ☐ Mombasa ☐ Mulago ☐ Soroti ☐ Mbale	**COAST ID:** ☐☐☐☐☐☐ **Child's initials:** ☐☐☐	**Clinic/Hospital number:** ☐☐☐☐☐☐☐☐☐

A: Oxygen therapy initiation

i. Time:	ii. SpO_2 (%):	iii. Intervention	iv. Method of delivery **(low flow only)**	v. Flow rate (l/min)	vi. FiO_2 (%)
H H : M M	☐☐☐	Opti / Low / No	Prongs / Catheter / Mask / Non-rebreather mask (NR)	☐☐ . ☐	☐☐

B: Change in oxygen therapy initiation

	**This column must be completed 15 minutes post-initiation**				
**Date:**	DD/MMM/YY	DD/MMM/YY	DD/MMM/YY	DD/MMM/YY	DD/MMM/YY
**Time:**	HH:MM	HH:MM	HH:MM	HH:MM	HH:MM
i. SpO_2 now (%):	☐☐☐	☐☐☐	☐☐☐	☐☐☐	☐☐☐
ii. Intervention type:	Opti / Low / No	Opti / Low / No	Opti / Low / No	Opti / Low / No	Opti / Low / No
iii. Method of delivery **(low-flow only)**:	Prongs / Cath / Mask / NR	Prongs / Cath / Mask / NR	Prongs / Cath / Mask / NR	Prongs / Cath / Mask / NR	Prongs / Cath / Mask / NR
iv. Flow rate (l/min) (No O_2 = 0):	☐☐☐ . ☐	☐☐☐ . ☐	☐☐☐ . ☐	☐☐☐ . ☐	☐☐☐ . ☐
v. O_2% delivered (In air = 21%):	☐☐	☐☐	☐☐	☐☐	☐☐
vi. Airway - suctioning required:	Yes / No	Yes / No	Yes / No	Yes / No	Yes / No
vii. Action taken:	☐	☐	☐	☐	☐
viii. If 6, indicate reason (1-5):	☐	☐	☐	☐	☐
Initials:					

0 - No change

1 - Change in the flow rate or amount of % O_2 given (no change in intervention type)

2 - Change in intervention type: SpO_2 <80%: start control to low flow (COAST B only)

3 - Restarting O_2 therapy if SpO_2 <92%: Optiflow and low flow only (not control)

4 - Change at 48 hours if SpO_2 <92%: Optiflow to low flow, control to low flow

5 - Trial of weaning from O_2 therapy over 15 minutes - if fails, check no changes made

6 - Problems with giving O_2 therapy: 1) Power cut 2) O_2 run out 3) Child unable to tolerate 4) Nasal trauma or facial trauma 5) On nebulization (>15 minutes off O_2 therapy)

COAST	Form 6: Oxygen therapy administration continued	PLACE STICKER HERE<br>23-March-2017 v.1.2
**Centre:** ☐ Kilifi ☐ Mombasa ☐ Mulago ☐ Soroti ☐ Mbale	**COAST ID:** ☐☐☐☐☐☐ **Child's initials:** ☐☐☐	**Clinic/Hospital number:** ☐☐☐☐☐☐☐☐☐

**Date:**	DD/MMM/YY	DD/MMM/YY	DD/MMM/YY	DD/MMM/YY	DD/MMM/YY
**Time:**	HH:MM	HH:MM	HH:MM	HH:MM	HH:MM
i. SpO_2 now (%):	☐☐☐	☐☐☐	☐☐☐	☐☐☐	☐☐☐
ii. Intervention type:	Opti / Low / No	Opti / Low / No	Opti / Low / No	Opti / Low / No	Opti / Low / No
iii. Method of delivery **(low-flow only)**:	Prongs / Cath / Mask / NR	Prongs / Cath / Mask / NR	Prongs / Cath / Mask / NR	Prongs / Cath / Mask / NR	Prongs / Cath / Mask / NR
iv. Flow rate (l/min) (No O_2 = 0):	☐☐.☐	☐☐.☐	☐☐.☐	☐☐.☐	☐☐.☐
v. O_2% delivered (In air = 21%):	☐☐	☐☐	☐☐	☐☐	☐☐
vi. Airway - suctioning required:	Yes / No	Yes / No	Yes / No	Yes / No	Yes / No
vii. Action taken:	☐	☐	☐	☐	☐
viii. If 6, indicate reason (1-5):	☐	☐	☐	☐	☐
Initials:					

**Date:**	DD/MMM/YY	DD/MMM/YY	DD/MMM/YY	DD/MMM/YY	DD/MMM/YY
**Time:**	HH:MM	HH:MM	HH:MM	HH:MM	HH:MM
i. SpO_2 now (%):	☐☐☐	☐☐☐	☐☐☐	☐☐☐	☐☐☐
ii. Intervention type:	Opti / Low / No	Opti / Low / No	Opti / Low / No	Opti / Low / No	Opti / Low / No
iii. Method of delivery **(low-flow only)**:	Prongs / Cath / Mask / NR	Prongs / Cath / Mask / NR	Prongs / Cath / Mask / NR	Prongs / Cath / Mask / NR	Prongs / Cath / Mask / NR
iv. Flow rate (l/min) (No O_2 = 0):	☐☐.☐	☐☐.☐	☐☐.☐	☐☐.☐	☐☐.☐
v. O_2% delivered (In air = 21%):	☐☐	☐☐	☐☐	☐☐	☐☐
vi. Airway - suctioning required:	Yes / No	Yes / No	Yes / No	Yes / No	Yes / No
vii. Action taken:	☐	☐	☐	☐	☐
viii. If 6, indicate reason (1-5):	☐	☐	☐	☐	☐
Initials:					

COAST

Form 7: Required observations

PLACE STICKER HERE
8-Feb-2017 v1.0

Centre: ☐ Kilifi ☐ Mombasa ☐ Mulago ☐ Soroti ☐ Mbale

COAST ID: ☐☐☐☐☐☐ **Child's initials:** ☐☐☐ **Clinic/Hospital number:** ☐☐☐☐☐☐☐☐☐

At:	**Hour 1**	**Hour 2**	**Hour 4**	**Hour 8**	**Hour 12**	**Hour 24**	**Hour 36**	**Hour 48**
**Date:**	DD/MMM/YY	DD/MMM/YY	DD/MMM/YY	DD/MMM/YY	DD/MMM/YY	DD/MMM/YY	DD/MMM/YY	DD/MMM/YY
**Time:**	HH:MM	HH:MM	HH:MM	HH:MM	HH:MM	HH:MM	HH:MM	HH:MM
i. Axillary temperature (°C):	☐☐.☐	☐☐.☐	☐☐.☐	☐☐.☐	☐☐.☐	☐☐.☐	☐☐.☐	☐☐.☐
ii. Heart rate (bpm):	☐☐☐	☐☐☐	☐☐☐	☐☐☐	☐☐☐	☐☐☐	☐☐☐	☐☐☐
iii. Systolic blood pressure (mmHg):	☐☐☐	☐☐☐	☐☐☐	☐☐☐	☐☐☐	☐☐☐	☐☐☐	☐☐☐
iv. Diastolic blood pressure: (mmHg):	☐☐☐	☐☐☐	☐☐☐	☐☐☐	☐☐☐	☐☐☐	☐☐☐	☐☐☐
v. Respiratory rate (brpm):	☐☐	☐☐	☐☐	☐☐	☐☐	☐☐	☐☐	☐☐
vi. Respiratory distress:	☐ Yes ☐ No	☐ Yes ☐ No	☐ Yes ☐ No	☐ Yes ☐ No	☐ Yes ☐ No	☐ Yes ☐ No	☐ Yes ☐ No	☐ Yes ☐ No
vii. Consciousness level:	A V P U	A V P U	A V P U	A V P U	A V P U	A V P U	A V P U	A V P U
viii. Oxygen saturation (%):	☐☐☐	☐☐☐	☐☐☐	☐☐☐	☐☐☐	☐☐☐	☐☐☐	☐☐☐
ix. Audible wheeze (0-3):<br>0: None, 1: Mild, 2: Moderate (in or out), 3: Severe (in and out)	☐	☐	☐	☐	☐	☐	☐	☐
x. On O_2 therapy	☐ Yes ☐ No	☐ Yes ☐ No	☐ Yes ☐ No	☐ Yes ☐ No	☐ Yes ☐ No	☐ Yes ☐ No	☐ Yes ☐ No	☐ Yes ☐ No
xi. Ability of vocalise (0-3):<br>0: Normal, 1: Some difficulty, 2: Severe difficulty, 3: Unable	☐	☐	☐	☐	☐	☐	☐	☐
xii. Ability to feed (0-3):<br>0: Normal, 1: Some difficulty, 2: Severe difficulty, 3: Unable	☐	☐	☐	☐	☐	☐	☐	☐
Blood:	Optional			Required	Optional			Required
i. Glucose (mmol/L):	☐☐.☐	☐☐.☐	☐☐.☐	☐☐.☐	☐☐.☐	☐☐.☐	☐☐.☐	☐☐.☐
ii. Lactate (mmol/L):				☐☐.☐				☐☐.☐
iii. Hb (g/L):				☐☐.☐				☐☐.☐
Initials:								

COAST (logo)

Form 8: Additional observations

PLACE STICKER HERE
8-Feb-2017 v1.0

Centre: ☐ Kilifi ☐ Mombasa ☐ Mulago ☐ Soroti ☐ Mbale

COAST ID: ☐☐☐☐☐☐ **Child's initials:** ☐☐☐ **Clinic/Hospital number:** ☐☐☐☐☐☐☐☐☐

**Date:**	DD/MMM/YY	DD/MMM/YY	DD/MMM/YY	DD/MMM/YY	DD/MMM/YY	DD/MMM/YY	DD/MMM/YY	DD/MMM/YY
**Time:**	HH:MM	HH:MM	HH:MM	HH:MM	HH:MM	HH:MM	HH:MM	HH:MM
i. Axillary temperature (°C):	☐☐.☐	☐☐.☐	☐☐.☐	☐☐.☐	☐☐.☐	☐☐.☐	☐☐.☐	☐☐.☐
ii. Heart rate (bpm):	☐☐☐	☐☐☐	☐☐☐	☐☐☐	☐☐☐	☐☐☐	☐☐☐	☐☐☐
iii. Systolic blood pressure (mmHg):	☐☐☐	☐☐☐	☐☐☐	☐☐☐	☐☐☐	☐☐☐	☐☐☐	☐☐☐
iv. Diastolic blood pressure: (mmHg):	☐☐☐	☐☐☐	☐☐☐	☐☐☐	☐☐☐	☐☐☐	☐☐☐	☐☐☐
v. Respiratory rate (brpm):	☐☐	☐☐	☐☐	☐☐	☐☐	☐☐	☐☐	☐☐
vi. Respiratory distress:	☐ Yes ☐ No	☐ Yes ☐ No	☐ Yes ☐ No	☐ Yes ☐ No	☐ Yes ☐ No	☐ Yes ☐ No	☐ Yes ☐ No	☐ Yes ☐ No
vii. Consciousness level:	A V P U	A V P U	A V P U	A V P U	A V P U	A V P U	A V P U	A V P U
viii. Oxygen saturation (%):	☐☐☐	☐☐☐	☐☐☐	☐☐☐	☐☐☐	☐☐☐	☐☐☐	☐☐☐
ix. Audible wheeze (0-3):<br>0: None, 1: Mild, 2: Moderate (in or out), 3: Severe (in and out)	☐	☐	☐	☐	☐	☐	☐	☐
x. On O_2 therapy	☐ Yes ☐ No	☐ Yes ☐ No	☐ Yes ☐ No	☐ Yes ☐ No	☐ Yes ☐ No	☐ Yes ☐ No	☐ Yes ☐ No	☐ Yes ☐ No
xi. Ability of vocalise (0-3):<br>0: Normal, 1: Some difficulty, 2: Severe difficulty, 3: Unable	☐	☐	☐	☐	☐	☐	☐	☐
xii. Ability to feed (0-3):<br>0: Normal, 1: Some difficulty, 2: Severe difficulty, 3: Unable	☐	☐	☐	☐	☐	☐	☐	☐
Blood:								
i. Glucose (mmol/L):	☐☐.☐	☐☐.☐	☐☐.☐	☐☐.☐	☐☐.☐	☐☐.☐	☐☐.☐	☐☐.☐
ii. Lactate (mmol/L):	☐☐.☐	☐☐.☐	☐☐.☐	☐☐.☐	☐☐.☐	☐☐.☐	☐☐.☐	☐☐.☐
iii. Hb (g/L):	☐☐.☐	☐☐.☐	☐☐.☐	☐☐.☐	☐☐.☐	☐☐.☐	☐☐.☐	☐☐.☐
Initials:								

COAST	# Form 9: Discharge from hospital	PLACE STICKER HERE<br>8-Feb-2017 v.1.0

COAST ID: ☐☐☐☐☐☐ **Child's initials:** ☐☐☐ **Clinic/Hospital number:** ☐☐☐☐☐☐☐☐☐

Centre: ☐ Kilifi ☐ Mombasa ☐ Mulago ☐ Soroti ☐ Mbale

A: Status at discharge from hospital

i.	Status at discharge:	☐ Alive ☐ Dead ☐ Absconded
ii.	If alive, date/time of discharge:	D D / M M M / 2 0 Y Y ☐☐ : ☐☐
iii.	If dead, date/time of death:	D D / M M M / 2 0 Y Y ☐☐ : ☐☐
iv.	If absconded, date/time last seen:	D D / M M M / 2 0 Y Y ☐☐ : ☐☐

If absconded child cannot be contacted by phone / home address and does not attend follow-up appointment, please complete Form 14: Lost to follow-up and withdrawal

B: Final diagnosis - *tick all that apply*

☐ LRTI - any	☐ Tuberculosis - any	☐ Urinary tract infection
☐ URTI - any	☐ Hepatitis - any	☐ Recurrent haemoglobinuria
☐ Asthma	☐ Meningitis - any	☐ Dark urine syndrome
☐ Other chest syndrome	☐ HIV / AIDS	☐ Severe anaemia (Hb <6g/dL)
☐ Severe malaria - any	☐ Developmental delay / cerebral palsy	☐ Malnutrition—any
☐ Sepsis/septicaemia	☐ Encephalopathy	☐ Pyrexia of unknown origin
☐ Sickle cell crisis	☐ Osteomylitis / Pyogenic arthritis	☐ Gastroenteritis

☐ Other, please state: ______________________________

Completed by: Name	Signature	Date
		D D / M M M / 2 0 Y Y

COAST	Form 10: Medications, fluids and blood	8-Feb-2017<br>v.1.0
**Centre:** ☐ Kilifi ☐ Mombasa ☐ Mulago ☐ Soroti ☐ Mbale	**COAST ID:** _ _ _ _ _ _ **Child's initials:** _ _ _	**Clinic/hospital number:** _ _ _ _ _ _ _ _ _

Drugs

Record all medications prescribed during admission

Drug	Dose (including units)	Frequency	Start date	Number of days received
			D D M M M Y Y Y Y	
			D D M M M Y Y Y Y	
			D D M M M Y Y Y Y	
			D D M M M Y Y Y Y	
			D D M M M Y Y Y Y	
			D D M M M Y Y Y Y	
			D D M M M Y Y Y Y	
			D D M M M Y Y Y Y	
			D D M M M Y Y Y Y	
			D D M M M Y Y Y Y	
			D D M M M Y Y Y Y	
			D D M M M Y Y Y Y	
			D D M M M Y Y Y Y	
			D D M M M Y Y Y Y	
			D D M M M Y Y Y Y	
			D D M M M Y Y Y Y	
			D D M M M Y Y Y Y	
			D D M M M Y Y Y Y	
			D D M M M Y Y Y Y	
			D D M M M Y Y Y Y	

Completed by: Name	Signature	Date
		D D / M M / 2 0 Y Y

COAST	Form 10: Medications, fluids and blood	8-Feb-2017<br>v.1.0
	COAST ID: ☐☐☐☐☐☐ Child's initials: ☐☐☐ Clinic/hospital number: ☐☐☐☐☐☐☐☐☐	

Fluids (including blood)

Fluid	Amount (ml)	Start date/time		End date/time	
		D D M M M Y Y Y Y	H H M M	D D M M M Y Y Y Y	H H M M
		D D M M M Y Y Y Y	H H M M	D D M M M Y Y Y Y	H H M M
		D D M M M Y Y Y Y	H H M M	D D M M M Y Y Y Y	H H M M
		D D M M M Y Y Y Y	H H M M	D D M M M Y Y Y Y	H H M M
		D D M M M Y Y Y Y	H H M M	D D M M M Y Y Y Y	H H M M
		D D M M M Y Y Y Y	H H M M	D D M M M Y Y Y Y	H H M M
		D D M M M Y Y Y Y	H H M M	D D M M M Y Y Y Y	H H M M
		D D M M M Y Y Y Y	H H M M	D D M M M Y Y Y Y	H H M M
		D D M M M Y Y Y Y	H H M M	D D M M M Y Y Y Y	H H M M
		D D M M M Y Y Y Y	H H M M	D D M M M Y Y Y Y	H H M M
		D D M M M Y Y Y Y	H H M M	D D M M M Y Y Y Y	H H M M
		D D M M M Y Y Y Y	H H M M	D D M M M Y Y Y Y	H H M M
		D D M M M Y Y Y Y	H H M M	D D M M M Y Y Y Y	H H M M
		D D M M M Y Y Y Y	H H M M	D D M M M Y Y Y Y	H H M M
		D D M M M Y Y Y Y	H H M M	D D M M M Y Y Y Y	H H M M
		D D M M M Y Y Y Y	H H M M	D D M M M Y Y Y Y	H H M M
		D D M M M Y Y Y Y	H H M M	D D M M M Y Y Y Y	H H M M
		D D M M M Y Y Y Y	H H M M	D D M M M Y Y Y Y	H H M M
		D D M M M Y Y Y Y	H H M M	D D M M M Y Y Y Y	H H M M
		D D M M M Y Y Y Y	H H M M	D D M M M Y Y Y Y	H H M M
		D D M M M Y Y Y Y	H H M M	D D M M M Y Y Y Y	H H M M

Completed by: Name	Signature	Date
		D D / M M / 2 0 Y Y

COAST	Form 11: Blood results	2-March-2017<br>v.1.0

COAST ID: ☐☐☐☐☐☐ **Child's initials:** ☐☐☐ **Clinic/Hospital number:** ☐☐☐☐☐☐☐☐☐

Centre: ☐ Kilifi ☐ Mombasa ☐ Mulago ☐ Soroti ☐ Mbale **Visit:** ☐ Admission ☐ Day 28 ☐ Day 90 ☐ Other

Date of form: D D / M M M / 2 0 Y Y

A: Haemotology - full blood count

Test	Result	Unit	Not done	Comments
i. WBC	☐☐☐☐.☐	☐ $10^3/\mu L$ ☐ $10^9/L$	☐	
ii. RBC	☐☐☐☐.☐	☐ $10^6/\mu L$ ☐ $10^{12}/L$	☐	
iii. Hb (from FBC) (g/dL):	☐☐☐☐.☐		☐	
iv. Haemocrit (%):	☐☐☐☐.☐		☐	
v. MCV (fL):	☐☐☐☐.☐		☐	
vi. MCH (pg):	☐☐☐☐.☐		☐	
vii. MCHC (g/dL):	☐☐☐☐.☐		☐	
viii. Platelets	☐☐☐☐.☐	☐ $10^3/\mu L$ ☐ $10^9/L$	☐	
ix. Lymphocytes	☐☐☐☐.☐	☐ $10^3/\mu L$ ☐ %	☐	
x. Neutrophils	☐☐☐☐.☐	☐ $10^3/\mu L$ ☐ %	☐	
xi. Granulocytes	☐☐☐☐.☐	☐ $10^3/\mu L$ ☐ %	☐	
xii. Monocytes	☐☐☐☐.☐	☐ $10^3/\mu L$ ☐ %	☐	

B: Malaria test

Test	Results	Not done	Comments
i. Malaria RDT:	☐ Positive ☐ Negative ☐ Invalid	☐	
ii. Malaria blood film:	☐ Positive ☐ Negative ☐ Invalid	☐	
iii. If the slide is positive:	Parasite count ☐☐☐☐	☐ Per 200 WBC<br>☐ Per 500 RBC	

C: Biochemistry

Test	Result	Unit	Not done	Comments
i. Sodium (mmol/L):	☐☐☐		☐	
ii. Potassium (mmol/L):	☐☐☐☐.☐		☐	
iii. Urea/BUN:	☐☐☐.☐	☐ mg/dL ☐ mmol/L	☐	
iv. Creatinine:	☐☐☐☐.☐	☐ µmol/L ☐ mg/dl	☐	

COAST	Form 11: Blood results	2-March-2017 v.1.0

COAST ID: ☐☐☐☐☐☐ **Child's initials:** ☐☐☐ **Clinic/Hospital number:** ☐☐☐☐☐☐☐☐☐

Centre: ☐ Kilifi ☐ Mombasa ☐ Mulago ☐ Soroti ☐ Mbale **Visit:** ☐ Admission ☐ Day 28 ☐ Day 90 ☐ Other

Date of form: D D / M M M / 2 0 Y Y

D: Microbiology

i. Pathogen isolated	☐ Yes ☐ No	
ii. If yes, provide details:		
iii. Results completed		**Date:** D D / M M M / 2 0 Y Y
iv. Signature:		

Completed by: Name	Signature	Date
		D D / M M M / 2 0 Y Y

COAST

Form 12: Follow-up

8-Feb-2017
v.1.0

COAST ID:		Child's initals:		Clinic/hospital ID:	

Centre: ☐ Kilifi ☐ Mombasa ☐ Mulago ☐ Soroti ☐ Mbale | Visit: ☐ 28 Days ☐ 90 Days ☐ Other

If patient does not attend as scheduled, please contact the carer and rearrange the visit, DO NOT COMPLETE this form at this point. If rearranging a visit is not possible, a telephone or home follow-up should be completed.

Complete this form on the rearranged visit date or when a telephone or home follow-up is conducted.

If the child does not attend follow-up appointment and cannot be contacted by phone / home address, please complete Form 14: Lost to follow-up and withdrawal

i. Type of follow-up: ☐ Clinic ☐ Home ☐ Telephone

ii. Date follow-up form completed: D D / M M M / 2 0 Y Y

A: Physical examination at follow-up

i. Weight (kg): _ _ . _	iii. MUAC (cm): _ _ . _	v. ☐ Not assessed—telephone
ii. Height (cm): _ _ _ . _	iv. Temperature (°C): _ _ . _	

B: Neurodevelopmental assessment (Day 28 or Day 90 only)

Symptom	Yes	No	Symptom	Yes	No
i. Abnormal vision:	☐	☐	iv. Abnormal behaviour:	☐	☐
ii. Abnormal hearing:	☐	☐	v. Abnormal movement / motor function:	☐	☐
iii. Abnormal speech production:	☐	☐	vi. Feeding difficulty:	☐	☐
vii. Abnormal comprehension	☐	☐	viii. Complete below from Source Document E:		

Gross motor: ☐ Pass ☐ Fail Fine motor: ☐ Pass ☐ Fail Communication: ☐ Pass ☐ Fail

ix. Any other symptoms: ____________________

x. Parental report/ worries: ____________________

C: Symptoms at follow-up

Symptom	Yes	No	Symptom	Yes	No
i. Fever:	☐	☐	viii. Abdominal aching /pain:	☐	☐
ii. Weight loss:	☐	☐	ix. Poor appetite:	☐	☐
iii. Severe pallor:	☐	☐	x. Sore mouth/throat /ulcers / thrush:	☐	☐
iv. Cough:	☐	☐	xi. Diarrhoea:	☐	☐
v. If yes, for more than 14 days:	☐	☐	xii. Other (specify): ____________		
vi. Difficulty breathing:	☐	☐	xiii. Other (specify): ____________		
vii. Bone or hand/foot pain:	☐	☐	xiv. Other (specify): ____________		

Completed by: Name	Signature	Date
		D D / M M M / 2 0 Y Y

COAST	# Form 12: Follow-up	8-Feb-2017 v.1.0

COAST ID: ☐☐☐☐☐☐ **Child's initals:** ☐☐☐ **Clinic/hospital ID:** ☐☐☐☐☐☐☐☐☐

Centre: ☐ Kilifi ☐ Mombasa ☐ Mulago ☐ Soroti ☐ Mbale | Visit: ☐ 28 Days ☐ 90 Days ☐ Other

D: Events since discharge / day 28 follow-up

Has the child had any of the following since discharge or Day 28 follow up:
(Do not complete at other visits, unless Day 28 follow-up was missed / delayed)

i. Admission to hospital: ☐ Yes ☐ No
If yes, add details below and complete **Form 13: SAE report**

Event name:	Event code: ☐☐.☐☐	Grade: ☐
Admission: D D / M M M / 2 0 Y Y	Discharge: D D / M M M / 2 0 Y Y	
Event name:	Event code: ☐☐.☐☐	Grade: ☐
Admission: D D / M M M / 2 0 Y Y	Discharge: D D / M M M / 2 0 Y Y	

ii. Blood transfusion: ☐ Yes ☐ No iii. Total number of units transfused: ☐☐☐☐

iv. Diagnosed with TB: ☐ Yes ☐ No v. Symptoms started: D D / M M M / 2 0 Y Y

vi. ☐ Presumptive ☐ Definitive vii. Treatment started: D D / M M M / 2 0 Y Y

viii. Acute febrile disease requiring medical intervention: ☐ Yes ☐ No

ix. Anti-malarials: ☐ Yes ☐ No x. Antibiotics: ☐ Yes ☐ No

xi. Any other illnesses requiring medical intervention: ____________________

xii. Visit to a local clinic or healthcare worker: ☐ Yes ☐ No xiii. Number of visits: ☐☐

xiv. Visit to a traditional healer: ☐ Yes ☐ No xv. Number of visits: ☐☐

xvi. Too ill to attend school: ☐ Yes ☐ No ☐ N/A xvii. Number of absences: ☐☐

xviii. Too ill to play or eat: ☐ Yes ☐ No xix. Number of occasions: ☐☐

E: Samples at follow-up

	Amount	Purpose	Taken
Lithium heparin (green top):	1 x 4ml	Plasma Storage	☐ Yes ☐ No
Additional samples taken:	☐ Yes ☐ No		
If yes, samples taken:	☐ Haemoglobin only ☐ FBC ☐ Malaria slide ☐ Malaria RDT		

Completed by: Name	Signature	Date
		D D / M M M / 2 0 Y Y

Form 13: SAE report

8-Feb-2017

v.1.0

COAST ID: ☐☐☐☐☐☐ **Child's initials:** ☐☐☐ **Clinic/Hospital number:** ☐☐☐☐☐☐☐☐☐

Centre: ☐ Kilifi ☐ Mombasa ☐ Mulago ☐ Soroti ☐ Mbale **Gender:** ☐ Male ☐ Female

Age: Y Y M M **Study arm:** ☐ COAST A ☐ COAST B **Intervention:** ☐ Opti-flow ☐ Low flow ☐ No O_2

A: General information

i.	Type of report: ☐ Serious Adverse Event ☐ Death
ii.	If SAE, stage of report: ☐ First ☐ Ongoing ☐ Final
iii.	If ongoing or final, date of first report: D D / M M M / 2 0 Y Y

B: Serious adverse event details

i. Why was the event serious (tick all that apply):

☐ Death	☐ Life-threatening (actual risk of death at the time of event)
☐ Persistent or significant disability or	☐ Caused or prolonged hospitalisation (excluding elective

☐ Other important medical condition (a real, not hypothetical, risk of one of the outcomes listed above, or intervention

Event Name	Code	Start Date	End Date	Ongoing	Grade
i.	☐☐.☐☐	D D M M M Y Y Y Y	D D M M M Y Y Y Y	☐	☐
ii.	☐☐.☐☐	D D M M M Y Y Y Y	D D M M M Y Y Y Y	☐	☐
iii.	☐☐.☐☐	D D M M M Y Y Y Y	D D M M M Y Y Y Y	☐	☐

C: Death

i. Location of death: ☐ Hospital ☐ Home ☐ Other: ____________________

ii. Date / time of death: D D / M M M / 2 0 Y Y H H : M M

iii. Underlying cause of death: ____________________

____________________ Code: ☐☐.☐☐

iv. Relationship of death to hypoxia (if unlikely or unrelated, please give reasons):

____

____

D: Assessment

Relationship to event:

i. Supplemental O_2	☐ Definitely	☐ Probably	☐ Possibly	☐ Unlikely	☐ Unrelated	☐ Can't assess	☐ N/A
ii. Delivery method	☐ Definitely	☐ Probably	☐ Possibly	☐ Unlikely	☐ Unrelated	☐ Can't assess	☐ N/A

Oxygen received: ☐ N/A - no O_2 received

i. Start date / time: D D / M M M / 2 0 Y Y H H : M M

ii. End date / time: D D / M M M / 2 0 Y Y H H : M M

	# Form 13: SAE report	8-Feb-2017<br>v.1.0

COAST ID: ☐☐☐☐☐☐ **Child's initials:** ☐☐☐ **Clinic/Hospital number:** ☐☐☐☐☐☐☐☐☐

E: Medication

Concomitant medication prior to the onset of the adverse event:

		Relationship of Adverse Event to medication		
**Concomitant Medication**	**Taking at event onset?**	**Definitely/Probably**	**Possibly**	**Unrelated/ Unlikely**
i. Steroids	☐ Yes ☐ No	☐	☐	☐
ii. Penicilin (Benzyl or Ampicillin)	☐ Yes ☐ No	☐	☐	☐
iii. Chloramphenical	☐ Yes ☐ No	☐	☐	☐
iv. Gentamicin	☐ Yes ☐ No	☐	☐	☐
iv. Ceftriaxone	☐ Yes ☐ No	☐	☐	☐
v. Artsunate	☐ Yes ☐ No	☐	☐	☐
vi. Salbutamol nebulisers / inhalers	☐ Yes ☐ No	☐	☐	☐
vii. Fluid bolus / rehydration	☐ Yes ☐ No	☐	☐	☐
viii. Transfusion	☐ Yes ☐ No	☐	☐	☐
ix. Other: ____________	☐ Yes ☐ No	☐	☐	☐
x. Other: ____________	☐ Yes ☐ No	☐	☐	☐

F: Description of SAE

i. Working diagnosis: ______________________________

ii. Clinical history, symptoms and signs: ______________________________

____

____

iii. Clinical examination: ______________________________

____

____

____

____

iv. Investigations: Malaria / RDT ________ Hb________ WBC ________ Lactate________ HIV status________

Other: ______________________________

v. Management: ______________________________

____

____

____

vi. Clinical Findings precipitating the event: ______________________________

____

Please continue to Source Document B: Additional Information as required

	Name	Signature	Date
**Form completed by** (Dr)			
**Local RA notified by /on**	☐ N/A		
To be completed by COAST Trial Coordinating Centre, Kilifi:			
**Clinically reviewed by**			

COAST	# Form 14: Lost to follow-up and withdrawal	PLACE STICKER HERE<br>8-Feb-2017 v.1.0

COAST ID: ☐☐☐☐☐☐ **Child's initials:** ☐☐☐ **Clinic/Hospital number:** ☐☐☐☐☐☐☐☐☐

Centre: ☐ Kilifi ☐ Mombasa ☐ Mulago ☐ Soroti ☐ Mbale

A: Lost to follow-up

i. Date patient last seen: D D / M M M / 2 0 Y Y

ii. Was this (tick below):

☐ Primary admission (absconded) ☐ At discharge ☐ 28 Days ☐ Other ☐☐☐ Days / Months

iii. Date of last contact with COAST staff, if different: D D / M M M / 2 0 Y Y ☐ Telephone ☐ Other

B: Home visit

i. Date of this home visit: D D / M M M / 2 0 Y Y ☐ N/A - no home visit

ii. Contact made with the patient during visit: ☐ Yes ☐ No

iii. If no, contact made with anyone with information on the patient: ☐ Yes ☐ No

iv. If known, reason patient absconded / has not attended follow up (tick all that apply):

☐ Moved to area with no trial clinic	☐ Caring for other family member	☐ No longer interested
☐ Living with another relative / carer	☐ Religious grounds	☐ Transport problems
☐ Died - complete Form 13	☐ Work commitments of carer	☐ Too ill to travel

☐ Other: ____________________

C: Withdrawal of consent

If a participant (carer or child) wishes to withdraw participation, they are able to do so without withdrawing consent for future contact and additional medical records to be used for COAST.
In the case of withdrawal, a Withdrawal of Consent Form should be completed.

i. Date withdrawal of consent form signed: D D / M M M / 2 0 Y Y

ii. Consent withdrawn for (tick all that apply):

☐ Continuing in COAST trial

☐ Access to medical records

iii. Reason for withdrawal of consent (tick all that apply):

☐ Moved to area with no trial clinic	☐ Caring for other family member	☐ No longer interested
☐ Living with another relative / carer	☐ Religious grounds	☐ Transport problems
☐ Work commitments of carer	☐ Too ill to travel	

☐ Other:

Completed by: Name	Signature	Date
		D D / M M M / 2 0 Y Y
