# Supplementary material for: Children’s Oxygen Administration Strategies Trial (COAST): A randomised controlled trial of high flow versus oxygen versus control in African children with severe pneumonia
Source: Wellcome Open Res. 2018 Jan 9;2:100. Originally published 2017 Oct 11. [Version 2] doi: 10.12688/wellcomeopenres.12747.2 (PMC5771148; doi:10.12688/wellcomeopenres.12747.2)
Supplement: Supplementary file 4 [file wellcomeopenres-2-14819-s0003.tgz › 89c277ad-e588-4b17-811b-45977d32cd92.pdf]

Data Monitoring Committee Charter

**Title of Study**	Children's Oxygen Administration Strategies Trial
**Sponsor**	Imperial College London
**Sponsor reference**	P46493
**Funder**	Joint Global Health Trials scheme:<br>Medical Research Council<br>Department for International Development<br>Wellcome Trust
**Funder reference**	MR/L004364/1
**ICREC reference**	15IC3100
**ISRCTN number**	ISRCTN15622505
**Chief Investigator**	Professor Kathryn Maitland
**Head Statistician**	Dr David Harrison

1. Introduction

The purpose of this document is to describe the roles and responsibilities of the Data Monitoring Committee (DMC) for COAST ('the trial'), including the relationship to other committees, frequency and format of meetings, methods of providing information to and from the DMC and decision making processes.

2. Roles and Responsibilities

2.1 Aims of the committee

Act as an independent advisory group to safeguard the interests of the trial's participants, investigators and sponsor. Further, to monitor the safety and efficacy of the trial's interventions and to monitor the trial's overall conduct to protect its validity and credibility.

2.2 Terms of reference

i) to determine if additional interim analyses of trial data should be undertaken
ii) to consider the unblinded data from interim analyses, plus any additional safety issues for the trial and relevant information from other sources
iii) after considering interim analyses and ensuring that the safety, rights and wellbeing of the trial participants are paramount, to report to the TSC and to recommend on the continuation of the trial
iv) to consider any requests for release of interim trial data and to recommend to the TSC on the advisability of this
v) in the event of further funding being required, to provide to the TSC, Sponsor and Funder appropriate information and advice on the data gathered to date that will not jeopardise the integrity of the study

2.3 Roles of the DMC

The DMC will have an independent chair with the authority to recommend early termination of the trial in the event of safety concerns or futility.

Together, the responsibilities of the committee are:

- to monitor the trial data and review any analysis as outlined in the Statistical Analysis Plan (SAP) or as requested by the TSC
- to monitor unblinded data and make recommendations to the TSC as to whether the trial is operating as expected, or if there are any ethical or safety reasons why the trial should not continue
- to consider data emerging from other related studies and its potential impact on the trial
- to monitor compliance with previous DMC recommendations

3. Before or early in the trial

All DMC members should have sight of the protocol before agreeing to join the committee. The trial has undergone review by the sponsor and scrutiny by other trial committees and local ethics committees. Therefore, if a potential DMC member has major reservations about the trial, they should report these to the Chief Investigator (CI) and the TSC and may decide not to accept the invitation to join. DMC member are independent and should be constructively critical whilst supportive of the aims and methods of the trial.

The DMC will meet in the first year of trial recruitment after the first interim analysis is complete. The purpose of the first meeting will be to:

- discuss the trial
- discuss the SAP
- allow opportunity for the DMC to clarify any aspects of the trial with the CI
- agree to the DMC Charter

The first report will contain tables and graphs based on real data where possible. 'Dummy' tables will be provided for data not yet available. This will act to familiarise DMC members with the format of reports and provide a forum to discuss changes and additions.

4. Composition

4.1 Membership
The DMC will be comprised of a Chair and at least three independent members, including at least one clinician and one statistician, all chosen because of their experience in trials and/or paediatric illness.

Members should not serve on DMCs of similar, concurrently active trials as this could compromise the independence and confidentiality of the individual trials. Any competing interests, real or potential, should be declared (see Appendix 1 for competing interest form).

The members of the DMC for COAST are:

Chair
Professor Tim Peto, Oxford University Hospitals, Oxford, UK

Other members
Dr Calum Semple (*clinician)*, Alder Hey Children's Hospital, Liverpool, UK

Professor Philippa Musoke (*clinician)*, Makerere University, Kampala, Uganda

Professor Fred Were *(clinician)*, University of Nairobi, Kenya

Dr Jim Todd *(statistician)*, National Institute of Medical Research, Mwanza, Tanzania

4.2 Role of the DMC Chair
The chair was chosen by the CI, and approved by the TMG and TSC. Their role is to facilitate and summarise discussions, and encourage consensus.

4.3 Role of the DMC Statistician
To provide independent statistical expertise and to further guide DMC members through the report. The DMC statistician is not expected to prepare the report.

4.4 Role of the Trial Statistician
The senior statistician, Dr David Harrison, and the trial statistical assistant, Zohra Zenasni, will be the main CTU representatives. They are responsible for the production of the SAP and the report to the DMC. They will participate in DMC meetings, guiding the DMC through the report and taking closed session notes.

4.5 Role of the Trial Coordination Team
The UK CTU trial coordinator, Daisy Wiley, will help produce the report for the open meeting. The trial coordinator may attend and take notes for open sessions of the meeting.

4.6 Role of the CI and other members of the TMG
The CI, Professor Kathryn Maitland, may be asked, and should be available to attend, open sessions of the DMC meeting. The other TMG members will not usually be expected but may attend open sessions when necessary.

5. Relationships

5.1 Relationship to other committees
The responsibilities of other committees are documented in the protocol. Figure 2 presents the relationship of the DMC to other trial committees. The DMC are an advisory body, making recommendations to the TSC who have executive oversight of the trial.

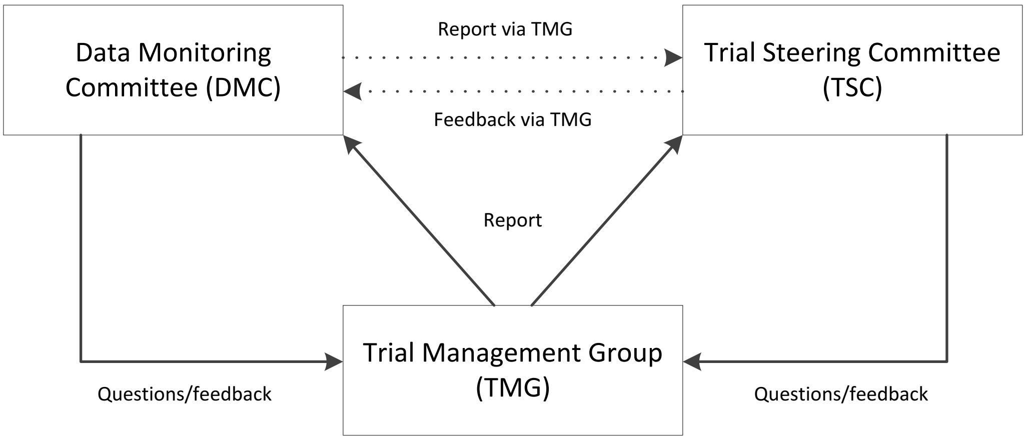

Figure 1. Relationship of DMC to other trial committees

5.2 Payments to the DMC
Members will be reimbursed for travel where required. No other payments or rewards will be given.

6. Meetings

The DMC will meet annually or as required. Due to the geographical distribution of DMC members, meetings will be held by teleconference. An unplanned DMC meeting may be called by the Chair or requested by the TMG if there are concerns for the safety of participants.

Proposed format
1. Open session: Introduction and 'open' report
2. Closed session: DMC discussion of 'closed' report

And, if necessary,
3. Open session: Discussion with other attendees on any matters arising from closed session
4. Closed session

7. Trial documentation and procedures to ensure confidentiality and proper communication

7.1 Intended content of material for open sessions
Accumulating information relating to recruitment and data quality will be presented. This will include number of patients eligible, number of patients randomised and completeness of validated outcome data. This information will be reported overall and by centre.

7.2 Intended content of material for closed sessions
The closed session material will include efficacy and safety data by treatment group.

7.3 Blinding of DMC
The DMC will not be blinded and will review outcome data according to treatment group.

Interim data and analyses, and the deliberations of the DMC, should be available only to those present in the closed sessions. The closed session DMC report is confidential and will only be seen by the DMC and the trial statistician(s). The DMC will not share this information with anyone outside of the committee, including the CI.

7.4 External evidence
The identification and circulation of external evidence is not the responsibility of the DMC. The CI and TMG will collate any such information for presentation in the open session.

7.5 Communication of DMC recommendations
The DMC will report its recommendations in writing to the TSC, via the trial statistician. If the trial is to continue unchanged, it is useful for the report from the DMC to contain a summary paragraph to this effect (see Appendix 2 for sample).

In its communications, the DMC should be careful not to relay any unnecessary information to the TSC, which may contain members of the TMG. The DMC should take care to shield interim trial results from the CI.

7.6 Reports to the DMC
It is anticipated that the DMC will receive the report at least two weeks before any meetings. DMC members should store the papers safely before and after each meeting. After the trial is reported, all interim reports should be destroyed.

8. Decision making

8.1 Recommendations open to the DMC
Possible recommendations include:
- No action needed, trial continues as planned
- Early stopping due to clear benefit /harm/futility/external evidence
- Modification of the trial design
- Early stopping of recruitment within a treatment arm/subgroup
- Extension of recruitment or follow up

8.2 Role of formal statistical analysis
Three interim analyses are planned following recruitment and follow-up (to 48 hours) of 350, 1575 and 2625 infants and children. Guidelines to recommend early termination will be based on a Peto-Haybittle stopping rule (P<0.001). A recommendation to discontinue recruitment, in all participants or in selected subgroups, will be made only if the results are likely to convince the general clinical

community and participants in COAST. Due to the stringent stopping criteria recommended, no adjustment will be made to the P value for the final analysis

8.3 Making recommendations

Effort should be made for all members to attend. The CTU will work to ensure a data is chosen it achieve this. The DMC will be deemed quorate if three members are present (to include the statistician and a clinician).

If the DMC is considering recommending a major action after the meeting, the Chair should talk with absent members as soon as possible after the meeting. If the member does not agree with proposed changes, a further teleconference should be arranged.

The DMC should endeavour to reach unanimous decisions. Where this is not possible, a vote may be held with the Chair holding the casting vote. Caution should be used when reporting the outcome of a vote to ensure information about trial data is not revealed.

9. Reporting

9.1 Reporting of recommendations

The DMC should report in writing to the TSC, usually within three weeks of the meeting. This should be sent via the trial statistician. Unless the DMC is recommending a change to the trial protocol, the letter should not usually reveal any confidential information. The letter should be copied to the CI, within any confidential information removed, and stored in the Trial Master File.

9.2 Minutes

A summary of main points with clear action points will be sufficient. Separate minutes reporting the open and closed session should be made. All members of the DMC will have opportunity to see and comment upon minutes, with the DMC chair responsible for sign-off.

9.3 Disagreement between DMC and TSC

If the DMC has serious problems or concerns with the TSC decision, a meeting of these groups should be held. The information to be shown depends upon the action proposed and the DMC's concerns. Depending on the reason for the disagreement, confidential data may have to be revealed to all those attending. The meeting should, therefore, be chaired by a senior member of the CTU or an external expert who is not directly involved with the trial.

10. After the trial

The CI has responsibility that trial results will be published in a correct and timely manner, the TSC will oversee this process.

After the trial, it is suggested that a meeting with DMC members and the CI is convened to give advice about data interpretation and provide opportunity to read and comment on publications before submission.

DMC members will be named and their affiliations listed in the main report, unless explicitly requested not to. A brief summary of timings and conclusions of DMC meetings will be included in the body of the report

The DMC may discuss issues from their involvement in the trial after the primary trial results have been published.

Appendix 1 - Agreement and potential competing interests form

Please initial:

I have read, understood and will work in accordance with the COAST DMC Charter	
I accept my responsibilities as a member of the COAST DMC	
I will ensure that all information made available to me and ensuing discussions will be treated in strict confidence	

The avoidance of any perception that members of the DMC may be biased in some fashion is important for the credibility of the decisions made by the DMC and for the integrity of the trial.

Possible competing interest should be disclosed via the ICNARC CTU. In many cases simple disclosure up front should be sufficient. Otherwise, the potential DMC member should remove the conflict or stop participating in the DMC. Table 1 lists potential competing interests, but is not exhaustive.

**No,** I have no competing interests to declare	
**Yes,** I have competing interests to declare (please detail below)	

Please provide details of any competing interests:

____

____

____

Name: ____

Signed: ____________________________ ________________ Date: ______________________________

Table 1: Potential competing interests

• Consulting arrangements with the Sponsor
• Frequent speaking engagements on behalf of the intervention
• Career tied up in a product or technique assessed by trial
• Hands-on participation in the trial
• Involvement in the running of the trial
• Emotional involvement in the trial
• Intellectual conflict e.g. strong prior belief in the trial's experimental arm
• Involvement in regulatory issues relevant to the trial procedures

Appendix 2 – Suggested report from DMC to TSC where no recommendations are being made

[Insert date]

To: Chair of Trial Steering Committee
Via: Trial statistician or Trial co-ordinator

Dear *[Chair of Trial Steering Committee]*

The Data Monitoring Committee (DMC) for the COAST met on *[XX/XX/XXXX]* to review its progress and interim accumulating data. *[List members]* attended the meeting and reviewed the report.

The DMC are satisfied with the running of the trial and its recruitment, data quality and follow-up. The trial question remains important and, on the basis of the data reviewed at this stage, we recommend continuation of the trial according to the current version of the protocol *[specify protocol version number and date]* with no changes.

We shall next review the progress and data *[provide approximate timing]*

Yours sincerely,

On behalf of the DMC (all members listed below)

DMC members:
(1)
(2)
(3)

Appendix 3 – Amendments to the charter

Amendment No.	Author(s) of changes	Details of changes made
