# Supplementary material for: Children’s Oxygen Administration Strategies Trial (COAST):  A randomised controlled trial of high flow versus oxygen versus control in African children with severe pneumonia
Source: Wellcome Open Res. 2018 Jan 9;2:100. Originally published 2017 Oct 11. [Version 2] doi: 10.12688/wellcomeopenres.12747.2 (PMC5771148; doi:10.12688/wellcomeopenres.12747.2)
Supplement: Peer review history

# Peer review: Referee response for version 2

**DOI:** 10.21956/wellcomeopenres.14819.r29614

**Published:** 2018-01-16

**Authors:** Steve Cunningham

**Affiliations:** 1 Department of Child Life and Health, University of Edinburgh, Edinburgh, UK

Thanks to Professor Maitland for the review feedback and clarification. I hope the Manual of Operations (with details of oxygen control strategies) will be made publically available in due course. This is a tremendous trial.

I have read this submission. I believe that I have an appropriate level of expertise to confirm that it is of an acceptable scientific standard.

# Peer review: Referee response for version 1

**DOI:** 10.21956/wellcomeopenres.13812.r26891

**Published:** 2017-10-30

**Authors:** Daniel S. Martin

**Affiliations:** 1 Centre for Altitude Space and Extreme Environment Medicine, Institute of Sport and Exercise Health, University College London, London, UK; 2 Royal Free Hospital, Pond St, Hampstead, NW3 2QG, UK

This superbly written methodology article describes a well thought out, carefully planned and excellently described study from a group of internationally recognised investigators. The research question is one of substantial importance to the delivery of healthcare in the developing world, that of the use of oxygen in acutely unwell children. The study has been carefully designed, based upon experiences gained from previous work carried out by this investigator group in a similar setting. The study is ambitious, but with their track record, this group are highly likely to complete this crucial piece of work and definitively answer the question they have raised. 

 The article itself is clear, concise and detailed; it contains a complete description of the background and study protocols that a readers requires. There appear to be no deficits in how the study has been outlined.

 One comment is that throughout the article the terms hypoxia and hypoxaemia have been used interchangeably. Hypoxia refers to a lack of oxygen at a cellular level. This, however, is a study of patients with hypoxaemia, a lack of oxygen in the blood. The term hypoxaemia should be used throughout, unless the authors are specifically referring to a cellular oxygen deficit. 

 There are one or two minor typographical errors in the manuscript. 

 I see absolutely no reason why this article should not be indexed and wish the investigators the very best of luck with this exciting piece of research. 

I have read this submission. I believe that I have an appropriate level of expertise to confirm that it is of an acceptable scientific standard.

# Response

**Published:** 2017-11-12

**Authors:** Kathryn Maitland

**Affiliations:** KEMRI-Wellcome Trust Research Programme, Kenya

Thank you for a very supportive review. I will edit the protocol once I have received the final reviewers comments to ensure all references to hypoxia are replaced by hypoxaemia and ensure all typos are also corrected. 

Kath Maitland on behalf of the COAST investigator group.

# Peer review: Referee response for version 1

**DOI:** 10.21956/wellcomeopenres.13812.r26893

**Published:** 2017-10-23

**Authors:** Steve Cunningham

**Affiliations:** 1 Department of Child Life and Health, University of Edinburgh, Edinburgh, UK

This is a well presented and detailed study protocol of a pivotal trial. The data underpinning the trial are considerable. The trial should significant enrich the quality of data for the management of hypoxaemic LRTI for children in developing healthcare settings.

 As a de novo reader of the protocol I have the following comments:

 Study Rationale:

 Justification for oxygen saturation target of ≤92%.

 Current WHO standard is a 90% oxygen saturation target. In applying a 92% target for oxygen supplementation the group directly challenge what is current practice in developing healthcare. Justification for this change is very limited within the protocol report; whilst 92% is current practice in a number of developed healthcare settings, it is without trial evidence (with some evidence as quoted that a 90% target has been trialled successfully in those under 12 months of age with bronchiolitis). There are two effects of this that it would be helpful to address within the protocol introduction (1) Maintaining supplemental oxygen above 90% is a significant healthcare resource (we estimated the difference in hospital stay to be 22 hours for a 94% rather than 90% target, so possible c10 hours per patient for 92%) (2) Recruitment to COAST B will be skewed to those with a higher baseline SpO _2_, by the distribution of SpO _2_ in acute respiratory disease; it might be estimated that 40% of all COAST B participants will arrive with a baseline SpO2 in the 90-92% range (Hooli 2016).  It would be very helpful if the protocol could discuss this probable skewed recruitment pattern to COAST B, in the context of the ability of the study to provide helpful data within an 81-89% SpO _2_ to enable a conclusion to be drawn incorporating current WHO guidelines. The number of children with SpO _2_ at baseline 90-91% who died in figures 3 and 4 are few and so the justification is important.

 Justification for limiting one arm of the study (high flow oxygen) to 48 hours regardless of clinical status at that time.

 In practice, from a clinical perspective this would be difficult to justify and it is most likely that high flow devices would not be withdrawn from children who continued to be critically ill at 48 hours. Please provide justification and method of assessment for how this might this influence outcomes post 48 hours in those who had high flow removed?

 Overemphasis on ‘equipoise’ for harms of supplemental oxygen

 The introduction provides significant weight to the potential harms of supplemental oxygen. The studies generally quoted consider extremely high concentration oxygen (>90%), something that will not be achieved within either study arm. There is no evidence of harm from low or high flow oxygen delivery systems, whilst there is clinical trial and pathophysiological theory for benefit. The introduction should be more balanced in this assessment. Whilst it is appropriate to consider potential adverse effects of an intervention (supplemental oxygen), this could not be considered equipoise for this intervention.

 Study Design

 Details of standard care

 What is the standard of care for children with lower respiratory tract infection and low oxygen saturation in the hospitals taking part? Is supplemental oxygen by nasal cannula/mask etc a standard of care. If so it would be helpful to state this. In the hospitals taking part, does standard of care include an ability to provide high flow oxygen, CPAP or intensive care.

 Data management appears appropriate. I would not consider myself appropriately qualified to comment on the statistical methodology.  

 Methodology details

 Detail of interventions

 It would be helped if the interventions could be more clearly outlined, i.e. what have local clinicians been told to apply, how and when. What is low flow oxygen, ?1 or 2 litres per minute of nasal cannula oxygen ?6 litres by mask, what is the maximum flow to be applied? How is the AIRVO2 high flow oxygen applied, how many litres per minute of flow and to what oxygen concentration. Is there a titration policy for starting, increasing and weaning oxygen supplementation and flow? Are clinicians responsive to SpO _2_ measurements. Is it possible within the trial for crossover, i.e. if at 30 hours SpO _2_ in permissive arm is 78% what would happen, if a child on low flow oxygen were deteriorating at 16 hours could they be transferred to high flow or to a CPAP device where available?

 10.5 Who will decide whether unexpected AEs are a result of the patients medical condition or standard hospital treatment. How will this be standardised across sites?

 11.6 Endpoint review committee. To whom will they report and with what frequency?

 The datasets are clear

 Additional point: Interchangeable use of the words hypoxia and hypoxaemia should be corrected. In almost all instances the word hypoxaemia is appropriate, i.e. low blood oxygen, as opposed to hypoxia which is critically low oxygen at tissue level. The challenge in healthcare is to understand what lower limits of hypoxaemia may be reliably tolerated without placing patients at risk of hypoxia and consequent hypoxic injury.

I have read this submission. I believe that I have an appropriate level of expertise to confirm that it is of an acceptable scientific standard, however I have significant reservations, as outlined above.

# Response

**Published:** 2017-11-15

**Authors:** Kathryn Maitland

**Affiliations:** KEMRI-Wellcome Trust Research Programme, Kenya

We thank Dr Cunningham for his very careful and thoughtful review. Before we address some of the concerns he raised we believe it is worthwhile to summarise the context of pneumonia management in most African hospitals.

Firstly, the burden of disease is substantial, with few hospitals able to sustainably deliver oxygen to most children requiring oxygen (Page 3). As oxygen cylinders are expensive to maintain (and frequently faulty) as the source of oxygen, the WHO’s preferred option is oxygen concentrators. The WHO conducted a survey of 231 health facilities in 12 African countries between 2007 and 2009. Only 44% reported uninterrupted oxygen supplies, 24% possessed a functioning oxygen concentrator and 35% had reliable power (Belle et al 2010; Page 5). Furthermore, most hospitals do not use pulse oximetry to target oxygen therapy, relying on clinical signs of pneumonia – which are very non-specific and thus only identify few children with hypoxaemia (Page 4). Thus, usual care is poorly targeted oxygen therapy with unreliable supplies of oxygen. The vast majority of hospitals have no access to intensive care outside of private health facilities and very few have access to CPAP or high flow/AirVO _2_ (these are largely donor driven). This includes the hospitals involved in the COAST trial.

A WHO ‘Evidence for technical update of pocket book recommendations’ was conducted in 2012 and recommended that oxygen therapy should be given to all children with signs of severe pneumonia or hypoxaemia (oxygen saturations < 90%) rated as a *Strong recommendation, very low quality evidence *(Pages 5-6).  The technical advisory group identified the following research gaps: ‘clinical studies comparing outcomes when oxygen is given at different thresholds’ and ‘large-scale effectiveness trials of improved oxygen systems on outcomes from pneumonia’. The COAST trial was designed to directly address these questions in children in Africa, including consideration of both lower and higher thresholds than the current recommendation.

With regard to the balance between presenting the potential benefits and harms of oxygen therapy, there are no relevant studies/trials to support the current recommendation and ‘oxygen toxicity’ is a relatively novel concept in Africa – and required substantial justification (on request during ethics review). We do not believe that we have provided an imbalanced case for the need for a trial.  A substantial section of the introduction provides substantial evidence of poor implementation and challenges faced by health services. However, even if these challenges were addressed with oxygen systems being prioritised there remains a scientific question about the role of oxygen as a life-saving drug. The data provided from the FEAST trial (control arm) and KEMRI Wellcome Trust Programme Kilifi Admission Cohort (all unselected admissions) who were all had access to oxygen and routine medications to treat underlying infections (provided by external funding) provided the data on which our sample size was calculated. These underpinned the need for a trial – even in context of a clinical trial (with very close monitoring), outcome in children with hypoxaemia was unsatisfactory (Pages 7-9).    

Selecting a higher threshold for investigation (<92% rather than <90%) enables the trial to investigate whether, if oxygen therapy is beneficial, the WHO threshold should be revised to include targeting a higher threshold (which is compatible with international recommendations). Logically, following on from this argument, if we had targeted < 90% and the COAST trial confirms that oxygen is life-saving then a future trial to examine whether it should be targeted to children with saturations 90-91% would require a massive trial, with little hope of securing funding. The COAST trial includes a health economic evaluation (from a health services perspective) so we will be able to provide future policy makers with data on both the clinical and cost-effectiveness of alternative oxygen strategies, taking into account baseline oxygen saturation.

Dr Cunningham questions limiting high flow/AirVO _2_ therapy to 48 hours (the primary endpoint). All children irrespective of arm after 48 hours will continue with use of low flow oxygen if needed. The 48-hour endpoint was selected since it captures ~ 85% of the mortality of the COAST participants and therefore strategies to improve outcome should focus on the high risk period; where the majority of impact of a life-saving therapy is likely to occur. Currently, the standard of care is not to give high flow/AirVO _2_ therapy and, pragmatically, using high flow/AirVO _2_ over days or weeks following admission would limit the availability of the machine, and therefore the numbers that could be recruited, with little potential for added improvement in mortality. If the COAST trial were to show that AirVO _2_ therapy improved outcome at 48 hours, we would also have captured important data on the 28 day outcome of all children, which may lead to refinements in guidelines in future implementation trials (balancing efficacy over cost-effectiveness). The latter consideration for policy makers is extremely important in resource-limited health services.  

Dr Cunningham points out that the protocol does not include information about the flow rates, oxygen/air blending titration and weaning protocols. However, these are covered in great detail in the Manual of Operations, that ‘operationalises’ the trial protocol which the clinical teams have been taught to follow. This is a live document that from time to time is revised to address evolving challenges. This is a very standard method for ensuring compliance and flexibility of the implementation of the trial – and reduces the number of times that the protocol needs to be returned to all three ethics committees (Imperial, Kenya and Uganda) and regulatory bodies for minor adjustments in the SOPs.

There is no cross-over permitted between arms. Providing oxygen to the permissive hypoxaemia arm if saturations ever fall below 80% is part of the randomised strategy for this arm so this is not cross-over. High flow/AirVO _2 _is the experimental therapy, by including a cross-over to this arm for children not responding to their alternative oxygenation strategy (low flow or permissive) suggests that we already know in the context in which the trial is being conducted that this is a superior strategy. There are no data to support this. The COAST trial will generate these data, and the effectiveness of high flow/AirVO _2_ cannot be assumed until demonstrated in an adequately powered trial.

All SAE reports are prepared by the clinicians and finalised by the site Principal Investigator, who attribute causality as per a detailed SOP for SAE reporting, for which there was an extensive training during the site initiation visit.  These are sent to the SAE reviewer in Kilifi (a clinician) and copies sent to the national ethical review committees. The SAE reviewer is responsible for checking completeness of the report and active dialogue with the responsible clinician/PI to ensure all are reported according to the SAE SOP. The SAE reviewer sends unblinded copies to the DMC for review during DMC meetings. The SAE reviewer also sends copies blinded to trial arm to the Endpoint Review Committee (chaired by Dr Jennifer Evans) who adjudicate on the mode of death and whether this was causally related to trial interventions. This is the method of SAE reporting we used in the FEAST trial and currently in our transfusion trial (TRACT).
